# Nascent Proteome Remodeling following Homeostatic Scaling at Hippocampal Synapses

**DOI:** 10.1016/j.neuron.2016.09.058

**Published:** 2016-10-19

**Authors:** Christoph T. Schanzenbächer, Sivakumar Sambandan, Julian D. Langer, Erin M. Schuman

**Affiliations:** 1 Max Planck Institute for Brain Research, Max von Laue Strasse 4, 60438 Frankfurt am Main, Germany; 2 Max Planck Institute for Biophysics, Max von Laue Strasse 3, 60438 Frankfurt am Main, Germany

**Keywords:** proteomics, homeostatic scaling, transcriptomics, protein synthesis, synaptic plasticity, BONCAT

## Abstract

Homeostatic scaling adjusts the strength of synaptic connections up or down in response to large changes in input. To identify the landscape of proteomic changes that contribute to opposing forms of homeostatic plasticity, we examined the plasticity-induced changes in the newly synthesized proteome. Cultured rat hippocampal neurons underwent homeostatic up-scaling or down-scaling. We used BONCAT (bio-orthogonal non-canonical amino acid tagging) to metabolically label, capture, and identify newly synthesized proteins, detecting and analyzing 5,940 newly synthesized proteins using mass spectrometry and label-free quantitation. Neither up- nor down-scaling produced changes in the number of different proteins translated. Rather, up- and down-scaling elicited opposing translational regulation of several molecular pathways, producing targeted adjustments in the proteome. We discovered ∼300 differentially regulated proteins involved in neurite outgrowth, axon guidance, filopodia assembly, excitatory synapses, and glutamate receptor complexes. We also identified differentially regulated proteins that are associated with multiple diseases, including schizophrenia, epilepsy, and Parkinson’s disease.

## Highlights

• Newly synthesized proteins were metabolically labeled during homeostatic scaling
• Approximately 6,000 newly synthesized proteins were detected using mass spectrometry
• Over 300 proteins were differentially regulated by synaptic up- and down-scaling
• Important synaptic proteins and disease-related proteins were regulated

## Teaser

Schanzenbächer et al. discover the newly synthesized proteome in response to the global activity manipulations that bring about homeostatic synaptic scaling. The synthesis of over 300 proteins was significantly differentially regulated by the induction of scaling, including neurotransmitter receptors, scaffolding, and signaling molecules.

## Introduction

Neuronal networks are subject to fluctuations in both the magnitude and frequency of inputs, requiring plasticity mechanisms to stabilize network activity. Homeostatic synaptic scaling adjusts the strength of neuronal connections up or down in response to changes in input (Davis, 2013, Turrigiano, 2012). At the *Drosophila* neuromuscular junction, genetic manipulations that alter postsynaptic receptor function result in compensatory changes in presynaptic neurotransmitter release (see Davis, 2013 for review). At mammalian synapses, blocking action potential-mediated activity either pharmacologically (O’Brien et al., 1998, Turrigiano et al., 1998) by occluding sensory input (Desai et al., 2002) or by suppressing excitability (Burrone et al., 2002) can lead to a global up-scaling of synaptic responses. Conversely, a global stimulation of activity, effected by blocking GABA_A_ receptor-mediated inhibition, leads to a significant down-scaling of synaptic responses (O’Brien et al., 1998, Turrigiano et al., 1998).

What is the nature of the molecular changes that bring about the synaptic scaling at mammalian synapses? Several groups have postulated that fluctuations in intracellular Ca^2+^ levels initially “sense” changes in activity (Ibata et al., 2008, Thiagarajan et al., 2005) that may be read out by changes in the activation of CaMKIV resulting in changes in gene expression (Ibata et al., 2008). In addition, the expression of scaling up is mediated by the accumulation of GluR2-containing receptors (Gainey et al., 2009, Gainey et al., 2015), likely driven by alterations with the glutamate receptor-interacting protein-1, GRIP-1 (Gainey et al., 2015), and protein-interacting-with C Kinase 1, PICK1 (Anggono et al., 2011). Other molecules implicated in various aspects of synaptic scaling include the neurotrophin brain-derived neurotrophic factor (BDNF) (Rutherford et al., 1998), the immediate early gene Arc (Shepherd et al., 2006), the cytokine TNFα (Steinmetz and Turrigiano, 2010, Stellwagen and Malenka, 2006), the immune molecule MHC1 (Goddard et al., 2007), β3 integrins (Cingolani et al., 2008), and the scaffold proteins PSD-95 and PSD-93 (Sun and Turrigiano, 2011). All of the above molecules were discovered using a candidate-based approach, making good “guesses” about the players that might be important, leaving open the possibility that many important molecules have not yet been discovered.

The long time course (∼24 hr) required for most forms of synaptic homeostasis to be established (O’Brien et al., 1998, Turrigiano et al., 1998) suggests a requirement for changes in gene expression, either by regulated transcription or translation, or both. Indeed, upscaling elicited by a 24 hr TTX treatment is blocked by a transcription inhibitor (Ibata et al., 2008). In a form of homeostatic depression elicited by ChR2-stimulation of individual neurons, a requirement for both transcription and translation has been reported (Goold and Nicoll, 2010). Whether the classic homeostatic plasticity, elicited pharmacologically, requires changes in new protein translation has, surprisingly, not been addressed.

What is known about the transcriptomic and proteomic alterations that underlie homeostatic plasticity? A recent study studied a few selected transcripts with qPCR and showed that homeostatic upscaling resulted in a significant increase in the *Gria1*, *Tet1*, and *Gadd 45b* transcripts (Meadows et al., 2015). While alterations in transcripts can lead to changes in protein abundance, several studies have shown that in complex systems the correlation between the transcriptome and the proteome can be low (Gygi et al., 1999, Komatsu and Hossain, 2013, Jovanovic et al., 2015). As proteins are the primary and most common effectors in all cellular processes, it is important to determine the proteome directly to understand how plasticity fundamentally alters the neuronal and synaptic landscape.

Recent advances in proteomic technology have led to unprecedented coverage and depth in the identification of proteins in cells and tissues. A recent study, for example, identified over 8,000 proteins in each of several different brain areas, including the hippocampus (Sharma et al., 2015). In addition, over 2,000 proteins reliably associated with the postsynaptic density have also been identified (Distler et al., 2014, Peng et al., 2004, Pielot et al., 2012, Cajigas et al., 2012). Quantitative proteomic analyses that focus on the entire (unlabeled) proteome, however, do not allow one to identify proteins synthesized in response to plasticity. For example, if one observes an increase in protein levels, it is impossible to determine whether the increase is due to enhanced protein synthesis or decreased protein degradation. In order to isolate the analysis of the newly synthesized proteome associated with plasticity, metabolic labeling is required. Bio-orthogonal non-canonical amino acid tagging (BONCAT) enables pulsed metabolic labeling of newly synthesized proteins using non-canonical amino acids like azidohomoalanine (AHA) together with click-chemistry followed by mass spectrometry (Dieterich et al., 2006, Dieterich et al., 2007). Using this method, alterations in newly synthesized proteins can be directly determined. This approach has been applied to identify newly synthesized proteins in many systems (Landgraf et al., 2015), including in the hippocampus following treatment with dopamine (Hodas et al., 2012) or BDNF (Bowling et al., 2016).

The opposite phenotypes elicited during synaptic up-scaling and down-scaling represent a unique opportunity to examine the underlying proteomic plasticity and identify pathways that are bi-directionally or coordinately regulated. Here we detect an unprecedented number of newly synthesized proteins and provide a comprehensive map of the newly synthesized proteome following both up- and down-scaling.

## Results

To ascertain, first, whether pharmacologically elicited synaptic scaling requires protein synthesis, we conducted experiments in which protein synthesis was inhibited during the scaling manipulation (Figure S1). In order to elicit up-scaling, cultured hippocampal neurons were treated with the Na^+^ channel antagonist tetrodotoxin (1 μM) for 24 hr to elicit an increase in miniature excitatory postsynaptic current (mEPSC) amplitude (Figure S1A). The co-application of the protein synthesis inhibitor anisomycin (40 μM) prevented the mEPSC amplitude increase (Figure S1B). In order to elicit down-scaling, cultured hippocampal neurons (DIV 21) were treated with the GABA_A_ receptor antagonist bicuculline (40 μM) for 24 hr to elicit a decrease in mEPSC amplitude (Figure S1A). The co-application of the protein synthesis inhibitor anisomycin (40 μM) prevented the mEPSC amplitude decrease, indicating a requirement for new protein synthesis (Figure S1C). Taken together, these data demonstrate that protein synthesis is required for both up- and down-scaling elicited pharmacologically in cultured neurons.

**Figure 1:**
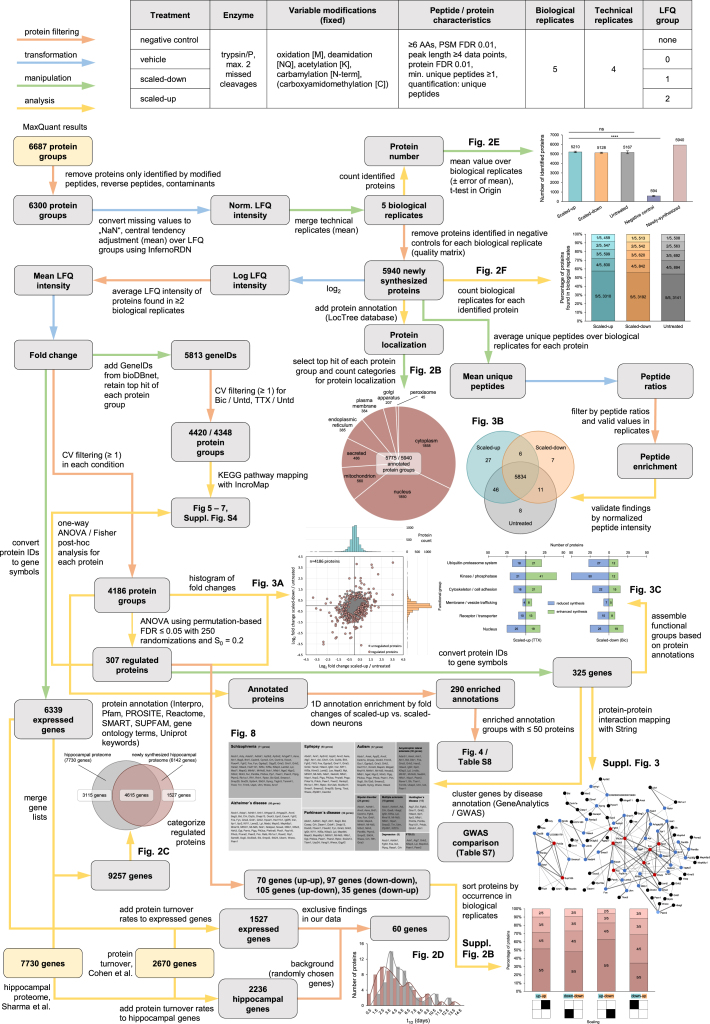
Overall Workflow Including Sample Structure, Preparation, Analyses and Data Processing, and Evaluation Flow chart explaining the datasets used, how the data were filtered, and how we calculated the numbers of proteins in each figure as well as the conversion to genes, when appropriate.

**Figure 2:**
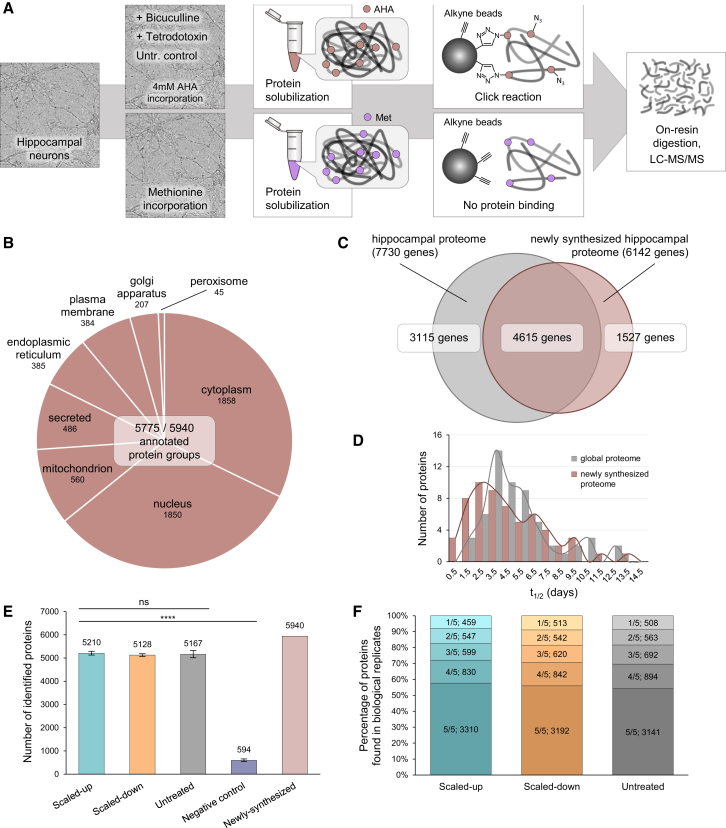
Workflow and Characterization of the Newly Synthesized Proteome (A) Workflow of experiments. Cultured hippocampal neurons (DIV 21) were treated with AHA (4 mM) and either vehicle, TTX (1 μM), or bicucculine (20 μM) for 24 hr. Control cultured neurons were exposed to methionine (4 mM) for 24 hr. Neurons were then harvested, and proteins were solubilized and clicked to alkyne-bearing beads. An on-resin digestion was performed, and peptides were then identified via liquid chromatography-mass spectrometry (LC-MS/MS). Proteins were identified and quantified using MaxQuant and other tools (see Experimental Procedures). (B) Representation of protein groups in different cellular compartments. (C) Venn diagram of the global hippocampal proteome (identified in Sharma et al., 2015) compared to the newly synthesized proteome (identified by 24 hr of AHA labeling). (D) Plot of protein half-lives for the global versus newly synthesized proteomes. Available data from Cohen et al. (2013) for 60 AHA-labeled and global proteome proteins are shown. There is a trend for shorter half-lives of the AHA-identified proteins. (E) Graph indicating the number of identified proteins across biological replicates for each group. Homeostatic scaling does not change the size of the nascent proteome. A significantly smaller number of proteins were identified in the negative control (methionine-treated) group (p < 0.0001), but none of the other groups differed significantly from one another. Error bars represent ± SEM. (F) Reproducibility of protein identifications across biological replicates within each group.

In order to identify the specific proteins synthesized following global manipulations of neuronal activity, we used BONCAT (Figures 1 and 2; Dieterich et al., 2006) together with the paradigm of homeostatic scaling. For the duration of the scaling induction (24 hr of either bicuculline or TTX, as above) cultured hippocampal neurons (DIV 21) were treated with the non-canonical amino acid azidohomoalanine (AHA; 4 mM) to label the newly synthesized proteins associated with homeostatic scaling. A control group was exposed to AHA for 24 hr in the absence of either bicuculline or TTX. An additional negative control group was treated with methionine, the amino acid from which AHA is derived (Met; 4 mM) (Figure 2A). Following scaling induction, neuronal lysates were prepared and BONCAT (see Experimental Procedures) was performed to capture, purify, and identify the newly synthesized proteins (Figure 2A). For each set of experiments, we conducted five independent biological replicates that were each injected in four technical replicate LC-MS/MS runs (see Experimental Procedures; Figure 1 and S2). To identify proteins, we required a minimum of one tryptic peptide per protein (see Experimental Procedures; Table S9). With a few exceptions (see Experimental Procedures), any protein detected in a methionine control experiment was subtracted from our experimental dataset, and only proteins present in more than two biological replicates were retained for subsequent analysis.

In the above experiments, we obtained an unprecedented coverage and depth of the newly synthesized proteome. Our master dataset, comprising the union of the untreated, scaled-up, and scaled-down groups, includes 5,940 unique protein groups representing all key cellular processes, including metabolic enzymes, cell signaling, and structural proteins (Figures 1 and 2B; Table S1). We were able to detect most of the important neuronal membrane proteins including ion channels and a majority of the subunits of the excitatory and inhibitory neurotransmitter receptors, including AMPA and NMDA receptor subunits as well as GABA_A_ and GABA_B_ receptors (Figures 5 and S5; Tables S1 and S3). In addition, we identified many membrane proteins associated with presynaptic membranes and vesicles, including both vesicular and plasma membrane transporters (Figure 6; Tables S1 and S3). These data are described in much greater detail below.

In order to assess the depth and breadth of our newly synthesized proteome, we compared the newly synthesized proteome of control (not subjected to scaling) AHA-treated neurons to a global hippocampal proteome dataset (Sharma et al., 2015) (see Experimental Procedures; Figure 1). We found that there was substantial overlap between the pulse (24 hr)-labeled nascent hippocampal proteome and the global hippocampal proteome (Figure 2C). The AHA-labeled proteome also captured many proteins not identified in the global proteome, likely owing to the specific enrichment and reduced sample complexity of the AHA-proteome and the resulting expanded dynamic range. We considered the possibility that some of the AHA-exclusive proteins represent short-lived proteins that are less likely to be detected in the large global proteome sample. To examine this possibility, we compared the reported protein half-lives for the AHA-exclusive proteins to a comparable number of proteins from the global hippocampal proteome. Using data from Cohen et al. (2013), we extracted all of the half-life data for AHA-exclusive proteins analyzed in their dataset (n = 60) and compared the distribution of half-lives to the same number of proteins sub-sampled from the global hippocampal proteome. We observed a shift toward a shorter half-life in the distribution for AHA-labeled proteins (Figure 2D), suggesting that one feature of the AHA-exclusive proteins may be a faster turnover, making the proteins more difficult to capture and detect in global proteomic studies.

We next analyzed the newly synthesized proteome in control (untreated), scaled-up, and scaled-down samples. First we examined the total nascent proteome size and discovered that homeostatic scaling does not significantly alter the number of unique newly synthesized proteins in the total proteome. The total nascent proteome size in each group (control, scaled-up, and scaled-down) was between 5,100 and 5,200 proteins (Figures 1 and 2E; Table S1). In the negative control methionine-treated samples, there were ∼594 proteins detected. Notably, in each AHA-treated group, over ∼80% of the proteins were identified in 3/5 biological replicates and over ∼90% of the proteins were identified in 2/5 biological replicates (Figure 2F). As there was no significant difference in the newly synthesized proteome size between groups, these data indicate that homeostatic scaling is associated with a balanced stimulation and inhibition of synthesis of protein groups.

**Figure 3:**
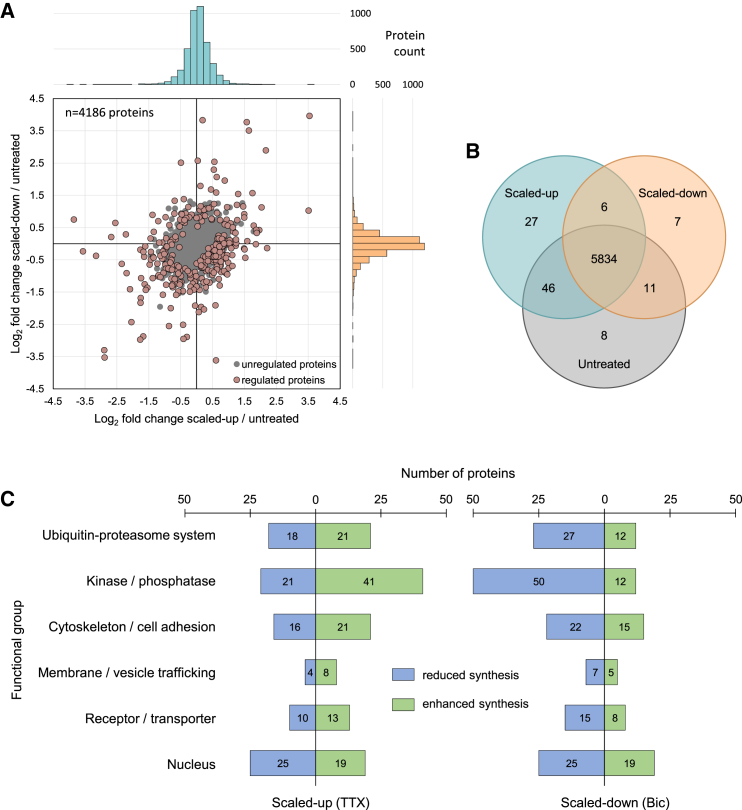
The Synthesis of ∼300 Proteins Is Differentially Regulated by Homeostatic Scaling (A) Scatterplot indicating every analyzed protein (n = 4,186). Proteins that were significantly regulated by homeostatic plasticity are indicated as mauve dots; all other proteins are gray. Histograms on the horizontal (teal) and vertical (orange) axes indicate the number of proteins that exhibited a given fold-change on the x (teal) and y (orange) axes. (B) Venn diagram depicting the total number and low-abundant, newly synthesized proteins identified in each of the following groups: scaled-up (TTX-treated), scaled-down (bicuculline-treated), or untreated (control). Proteins were manually extracted as outlined in Experimental Procedures. (C) Functional groups of significantly regulated proteins observed following up- or down-scaling.

We hypothesized that the dominant class of proteomic change during scaling would comprise changes in the abundance of proteins that perform essential cellular and synaptic functions. To analyze this, we compared protein abundances in the up-scaled or down-scaled nascent proteomes to control nascent proteomes using a label-free quantitation (LFQ)-based analysis (see Experimental Procedures; Figure 1). Altogether, we discovered over 300 proteins with significantly regulated expression levels following homeostatic plasticity (Figures 3A and S4; Table S2). This group comprises proteins that are co-regulated or singly regulated by either up- or down-scaling. Figure 3A depicts all proteins analyzed, and the four quadrants in the graph highlight four basic categories of significantly regulated newly synthesized proteins. Two quadrants depict proteins that exhibit coordinate regulation: both up- and down-scaling result in either an enhancement (upper right quadrant) or a reduction (lower left quadrant) in the synthesis of particular proteins. When compared to control samples, there were 70 proteins that exhibited enhanced protein synthesis associated with both up-scaling and down-scaling (Figure 3A, upper right quadrant). Some of the protein families that were overrepresented include the following: transcriptional activation, mRNA transport and splicing, and tyrosine phosphatases and kinases (Table S3). On the other hand, when compared to control samples, there were 97 proteins that exhibited reduced protein synthesis during both up-scaling and down-scaling (Figure 3A, lower left quadrant). Functional annotation analysis reveals a significant enrichment of protein families mediating inositol-1,4,5-triphosphate-sensitive calcium release, sphingomyelin degradation, and cell-cell adhesion essential for dendritic spine morphogenesis as well as other cellular functions (Table S3). Taken together, these two groups of proteins exhibit consistent enhanced or reduced synthesis, regardless of the sign of the plasticity, and thus might be considered as “general” scaling proteins that respond to global changes in neuronal activity.

**Figure 4:**
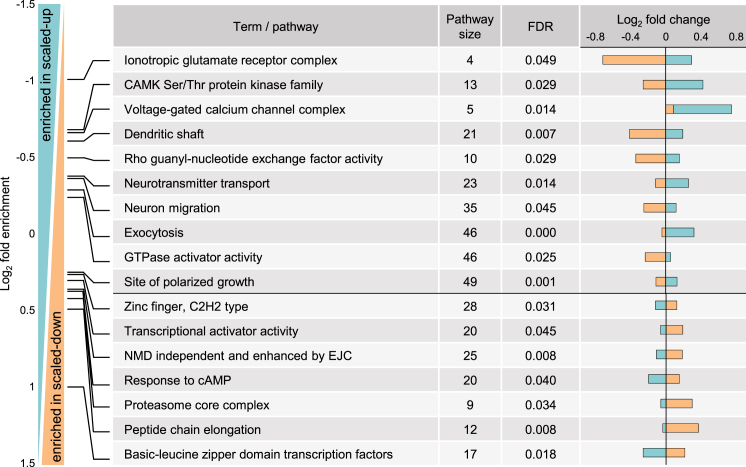
Differential Enrichment of Up- and Down-scaled Nascent Proteins Selected significantly regulated protein groups or pathways that exhibit opposite or differential regulation by up- and down-scaling (teal and orange bars, respectively). FDR = false discovery rate.

The remaining categories are represented by proteins significantly altered in a bi-directional manner: for these proteins, enhanced synthesis is associated with one form of plasticity (either up- or down-scaling) while reduced synthesis is associated with the other form (Figures 3A and 4; Table S2). When compared to control samples, there were 105 proteins that exhibited enhanced protein synthesis associated with up-scaling and reduced protein synthesis during down-scaling (Figure 3A, lower right quadrant). GO analysis of this group indicates a significant enrichment for proteins largely involved in synaptic function, including, for example, the family of Ca^2+^-calmodulin-dependent protein kinases (CamK) and proteins involved in neurotransmitter transport and exocytosis (Figure 4; Table S3). Indeed, the most differentially regulated group was the ionotropic glutamate receptor complex, the target of many mechanistic studies of homeostasis (Table S5) (Turrigiano, 2012). When compared to control samples, there were 35 proteins that exhibited enhanced protein synthesis during down-scaling and reduced protein synthesis during up-scaling (Figure 3A, upper left quadrant; Figure 4). Functional annotation analysis of this group indicates a significant enrichment of calcium-dependent protein serine/threonine phosphatases, splicing factors, transcription factors, calcineurin regulators, and synaptotagmins. Additional enriched proteins in this group are implicated in mRNA deadenylation, ubiquitin activation, cell growth, and other categories (Table S3). The above proteins should thus be considered as “specific” scaling proteins that respond with a specific signature to either homeostatic up- or down-scaling.

We also considered the entire population (∼307) of regulated nascent proteins as a single group and examined, using a String analysis, the networks of regulated proteins and the interactions between them (Figure S3). We identified one large network of regulated proteins containing 95 network members with the number of interaction partners ranging from 1 to greater than 7. Protein hubs in this network were represented by several protein kinases including the Fyn kinase, Lyn kinase, Map kinase 3, and Protein kinase C beta. In addition, other proteins such as the FGF receptor were linked to several other significantly regulated proteins. The remaining networks comprising significantly regulated proteins were small, ranging from 2 to 6 proteins, and the remaining regulated proteins were not linked to networks.

Finally, we considered the possibility that elements of the homeostatic proteomic response could be newly synthesized proteins that were uniquely and exclusively associated with either the control or scaled groups. We found no highly abundant (> 10 peptides) proteins exclusively in scaled-up or scaled-down neurons. As regulatory proteins may be low abundant or only yield a low number of detectable peptides, we manually inspected low-abundance proteins in our dataset based on reproducibility, spectral counting, and normalized peptide intensity ratios (for a detailed description, see Experimental Procedures). Acknowledging that this approach yields only trends rather than precise quantitative values, we identified 27 and 7 proteins with higher abundance in scaled-up neurons or scaled-down neurons, respectively (Figures 1 and 3B; Tables S1 and S2). Using these “relaxed” criteria, newly synthesized proteins enriched in scaled-up neurons were implicated in glutamatergic synapses and excitability (Adcy2, Grin2a), calcium transport (Atp2b3), nerve growth factor signaling (Lmtk2), and other cellular functions (Table S2). The newly synthesized proteins enriched in scaled-down neurons included proteins involved in synapse formation (Arhgap) and modulation of dendritic spines (Dgkz) (Table S2). An additional six proteins were found to be enriched in both scaled groups (Table S2), including proteins involved in modulation of dendritic spine shape (Synpo), regulation of the number of excitatory synapses (C1ql3), and axonal branching (Fgf2).

**Figure 5:**
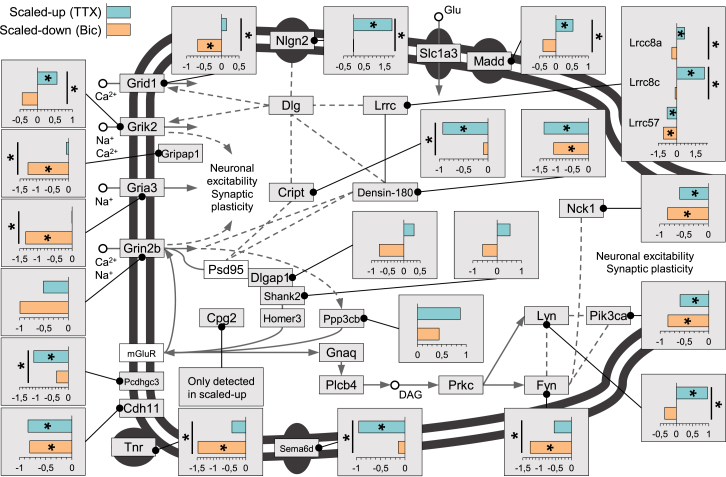
Proteins Associated with Postsynaptic Function at Excitatory Synapses that Are Regulated by Homeostatic Scaling The glutamatergic synapse. Proteins in white or gray boxes were detected in this study. Call-out boxes display intensity values (see Experimental Procedures). The bars, teal and ochre, represent the regulation of the protein in up- and down-scaling, respectively. Asterisks within the colored bar indicate that the protein exhibited significant (ANOVA FDR < 0.05 and Fisher LSD post hoc p < 0.05) regulation relative to control; asterisks associated with the black lines indicate the protein exhibited significant (ANOVA FDR < 0.05 and Fisher LSD post hoc p < 0.05) regulation between up- and down-scaling.

We next determined the changes that occurred in different cell types, compartments, and signaling pathways. Within the postsynaptic compartment (Figure 5; Table S4), we found several examples where the opposite phenotypic endpoints of homeostatic scaling are associated with opposing effects on glutamate receptors. For example, some subunits of AMPA-type (Gria3, Grid1) and a kainate-type (Grik2) receptor showed a pattern of upregulation during scaling-up accompanied in some cases (Grid1, Grik2) by downregulation during scaling-down, as expected if the receptors are modulated to increase or decrease sensitivity to neurotransmitter (Figure 5; Table S4). (Unfortunately, we did not obtain a sufficient number of unique peptides for Gria1 and 2 to conduct an analysis.) The NMDA receptor subunit Grin2b exhibited a trend for downregulated synthesis in both up- and down-scaling, although to a much greater extent during down-scaling (Figure 5). Other postsynaptic components that underwent regulation include Cript (Niethammer et al., 1998) and Lrcc7 (Densin-180). Map kinase 3 (Mapk3), a kinase implicated in long-term potentiation (LTP; Mazzucchelli et al., 2002) and implicated in autism spectrum disorders (Kumar et al., 2008), was significantly differentially regulated: enhanced in scaled-up conditions and reduced in scaled-down conditions (Tables S2 and S4). Several adhesion molecules, Tnr, Cadh11, Pcdhgc3, and Sema6d, were also significantly regulated with a clear trend for downregulation in both forms of scaling (Figure 5; Tables S2 and S4), reminiscent of earlier studies showing that downregulation of adhesion is required for structural plasticity at synapses (Mayford et al., 1992, Schuster et al., 1996). Nlgn2, in contrast, was significantly upregulated by homeostatic up-scaling (Figure 5; Tables S2 and S4).

**Figure 6:**
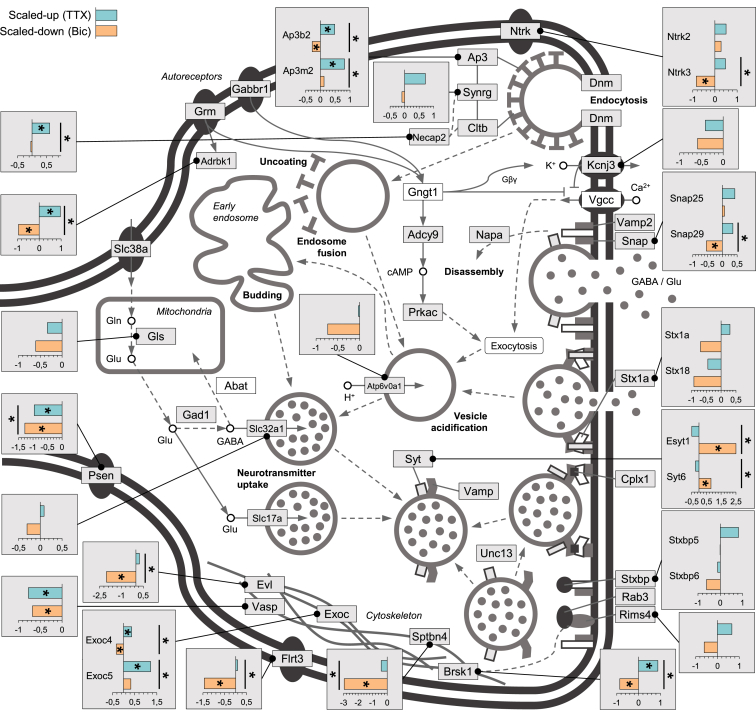
Proteins Associated with Presynaptic Function which Are Regulated by Homeostatic Scaling The presynaptic terminal. Proteins in white or gray boxes were detected in our samples; call-out boxes display intensity values (see Experimental Procedures). The bars, teal and ochre, represent the regulation of the protein in up- and down-scaling, respectively. Asterisks within the colored bar indicate that the protein exhibited significant (ANOVA FDR < 0.05 and Fisher LSD post hoc p < 0.05) regulation relative to control; asterisks associated with the black lines indicate the protein exhibited significant (ANOVA FDR < 0.05 and Fisher LSD post hoc p < 0.05) regulation between up- and down-scaling.

Within the functional area of vesicular trafficking and neurotransmitter release, we observed many significantly regulated proteins (Figure 6; Table S4). Some vesicle-associated proteins like members of the syntaxin family (Syt6 and Esyt1) exhibited enhanced synthesis following down-scaling, whereas Snap29, a protein involved in multiple membrane trafficking steps, and Brsk1, a protein implicated in neurotransmitter release, were significantly differentially regulated by homeostatic scaling and reduced following down-scaling (Figures 4 and 6). Some proteins in the Ap-3 family (Ap3b2 and Ap3m2), a heterotetrameric vesicle-coat protein complex that is important for protein sorting and clathrin recruitment, exhibited significantly different expression with up- and down-scaling and were significantly upregulated with up-scaling (Figures 4 and 6; Tables S2 and S4). Related to the actin cytoskeleton, several proteins (Evl, Sptbn4, and Vasp) showed extremely robust and significant regulation consistently with down-scaling, but also with up-scaling (Figure 6; Tables S2 and S4).

As homeostatic plasticity is known to involve regulation of inhibitory transmission (Hartman et al., 2006, Kilman et al., 2002, Maffei et al., 2004), we next examined the protein components of GABAergic synaptic transmission that exhibit altered protein synthesis (Figure S5). The family of GABA_A_ receptors was significantly regulated and exhibited, in general, opposite changes for up- and down-scaling (Figure S5). The GABA receptor scaffolding molecule Gephyrin showed a similar trend (Figure S5). Neuroligin 2 (Nlgn2), a protein implicated in the clustering of GABA receptors, and Kirrel3, a protein implicated in GABAergic synapse formation, both exhibited significant upregulation following up-scaling (Figure S5). Synthesis of the vesicular GABA transporter, Slc32a1, was reduced in down-scaled neurons and slightly enhanced in up-scaled neurons (Figure S5). Interestingly, Kif5a, a microtubule-dependent motor essential for the transport of GABA_A_ receptors (Nakajima et al., 2012) was significantly differentially regulated and upregulated in scaled-up neurons (Figure S5).

Since AHA-labeled newly synthesized proteins can originate from either the glial or neuronal cells present in our hippocampal cultures, we used bioinformatics (GeneAnalytics; see Experimental Procedures) to separately analyze the proteins associated with glial function. For the 5,940 proteins in the global AHA-labeled proteome, there are 6,339 associated genes; of these, 929 genes (14.7%) are associated with glial function (Table S6). For the 307 significantly regulated proteins in the AHA-proteome, there are 325 associated genes; of these, 41 genes (12.6%) are associated with glial function (Table S6). It is important to note that the GeneAnalytics tool we used identifies glial-associated genes, but does not identify glial-exclusive genes. As such, some of the regulated genes are shared with neurons, and the cell type where the protein is significantly altered—glia, neurons, or both—is not certain. Cell-type-specific labeling techniques (see Discussion) will certainly be useful to address this issue in the future.

**Figure 7:**
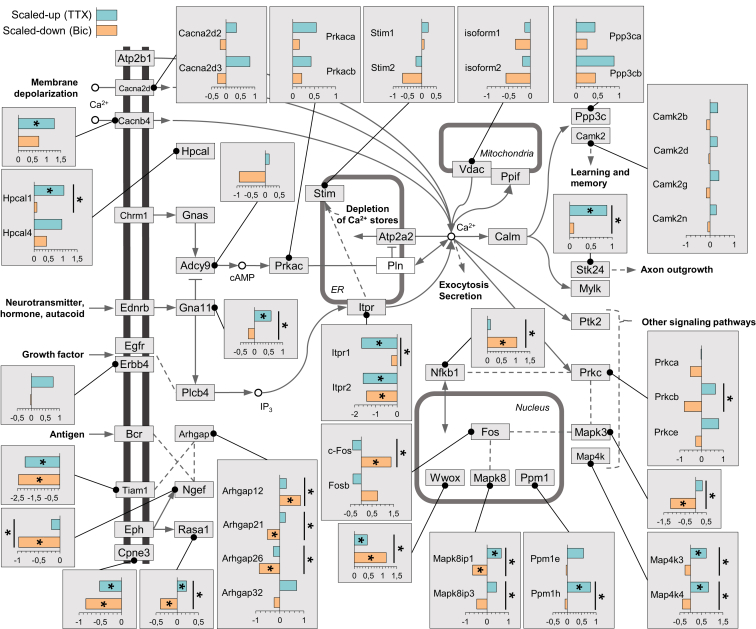
Ca^2+^ Signaling Pathway Proteins that Are Regulated by Homeostatic Scaling Intracellular signaling pathways. Proteins in white or gray boxes were detected in our samples; call-out boxes display intensity values (see Experimental Procedures). The bars, teal and ochre, represent the regulation of the protein in up- and down-scaling, respectively. Asterisks within the colored bar indicate that the protein exhibited significant (ANOVA FDR < 0.05 and Fisher LSD post hoc p < 0.05) regulation relative to control; asterisks associated with the black lines indicate the protein exhibited significant (ANOVA FDR < 0.05 and Fisher LSD post hoc p < 0.05) regulation between up- and down-scaling.

As Ca^2+^-regulated and synapse-to-nucleus signaling play a prominent role in all forms of plasticity, including homeostatic plasticity, we also searched for altered protein synthesis in these pathways (Figure 7; Table S4). The CamKII family of subunits, for example, showed a consistent trend of upregulated synthesis following scaling-up and downregulated synthesis following scaling-down (Figure 7; Table S2). A similar pattern was observed for the beta subunit of Protein kinase C (Prkcb) and several of the Map kinases (Mapk3, Map4k, and Mapk8) (Figure 7). The inositol trisphosphate receptor 2 (ITPR2) was also potently and significantly downregulated with both forms of plasticity, whereas the ITPR1 molecule was significantly downregulated exclusively with scaling-up (Figure 7). The family of Arhgaps, negative regulators of Rho GTPases, together with the positive regulator of Rho GTPases and dendritic morphology regulator Tiam1 (Um et al., 2014), represent a hotspot for significant regulation during homeostatic scaling.

**Figure 8:**
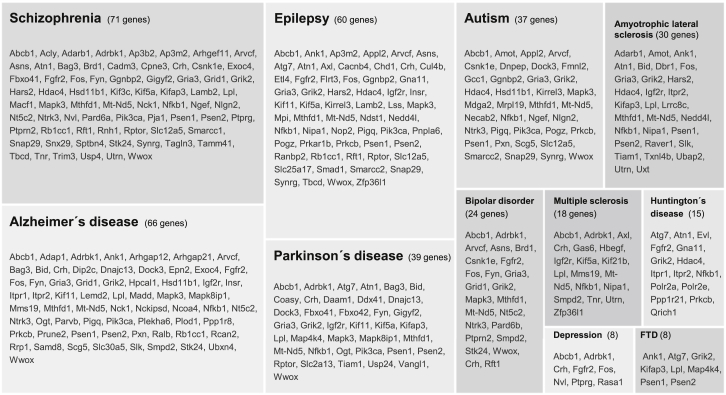
Neurological Disease-Associated Proteins Regulated by Homeostatic Scaling Shown are genes of significantly regulated proteins associated with each of the indicated diseases. Of the 325 genes regulated by scaling induction, 166 are associated with one of the diseases shown. Some genes are listed for multiple diseases.

In the above sections, we highlighted some proteins that are altered during synaptic scaling. As defects in synaptic scaling have been proposed to contribute to diseases such as Alzheimer’s disease (Pratt et al., 2011) and epilepsy (Trasande and Ramirez, 2007), we asked whether any of the regulated proteins identified in this study are associated with neurodevelopmental, psychiatric, or neurodegenerative disorders. Forty-five percent of the proteins regulated during homeostatic scaling had an association with a neural disease. As shown in Figure 8, we queried regulated proteins associated with Alzheimer’s disease, Parkinson’s disease, Huntington’s disease, schizophrenia, epilepsy, autism, amyotrophic lateral sclerosis, multiple sclerosis, bipolar disorder, depression, and frontotemporal dementia (see also Table S7) and found over 166 proteins that were associated with at least one of the above diseases. Within this set of disease-related regulated proteins, we identified that, for example, Presenilin-1 (Psen), previously reported to be essential for homeostatic scaling (Pratt et al., 2011), is significantly regulated in both up- and down-scaling (Figures 6 and 8; Table S2). We additionally examined the association of our regulated proteins with disease-related single-nucleotide polymorphisms identified in genome-wide association studies (GWAS) (Table S7). Here we again found that a large number (∼95) of the proteins regulated by synaptic homeostasis are associated with neurological dysfunction.

## Discussion

In this study, we examined the proteomic changes that underlie homeostatic scaling in primary hippocampal neurons. We demonstrate for the first time that both up- and down-scaling elicited by pharmacological manipulation of neuronal activity require new protein synthesis. Using BONCAT (Dieterich et al., 2006), we selectively labeled, purified, and analyzed the newly synthesized neuronal proteomes. Here, BONCAT enabled us to track and visualize proteome adaptations during homeostatic scaling that are usually extremely challenging to examine in tissue samples due to both the dynamic range of the proteome and the relatively small degree of differential regulation. We identified over 5,000 proteins in each experimental group as well as the control group. Homeostatic scaling did not alter the absolute number of distinct newly synthesized proteins, but rather resulted in differences in which proteins were synthesized and to what degree. We detected ∼300 proteins that exhibited significantly altered protein synthesis as a result of the global activity manipulations that induce homeostatic scaling. Recently, other groups have combined BONCAT with SILAC in order to include an additional validation step for new protein synthesis and to enable isotope-based, robust quantification (Bagert et al., 2014, Eichelbaum and Krijgsveld, 2014). Here we circumvented the need for SILAC, applying stringent control experiments and analyses and a highly stable LC-MS/MS setup, producing the largest dataset for the newly synthesized neuronal proteomes to date. We also investigated “low-abundance” proteins that are notoriously challenging to quantify due to the limited number of detectable peptides or low peptide intensities. We employed a combination of reproducibility, spectral counting, and normalized peptide intensities to manually evaluate a subset of low-abundance proteins, and as such these evaluations are “semi-quantitative”; the derived lists represent “trends” that may be of interest.

The significantly regulated newly synthesized proteins we identified span many functional categories and reside in different cellular compartments. Because of the time course of the plasticity and the duration of the metabolic labeling (24 hr), these proteins can represent regulated “sensors,” which respond to the activity manipulation, or “effectors,” which drive the implementation of the homeostatic responses. In addition to these proteins, we explicitly evaluated the previously published data documenting roles for various proteins in homeostatic scaling (Table S5). Of the 29 scaling-related proteins we identified in the literature, we detected 20 as newly synthesized in our dataset (∼69%). Of these 20 proteins, we detected either a trend for regulation or significant regulation associated with scaling for 65% percent. We also queried the extent to which our significantly regulated proteins have been associated with various neurological diseases and found that a large fraction (166 genes, ∼51%) have been implicated in one or more neurological diseases. An emerging view that incorporates the profound cumulative understanding that many neurological diseases are associated with mutations in hundreds to thousands of proteins is the notion that a loss of homeostasis at the level of neural circuits is causal (Ramocki and Zoghbi, 2008). The number of proteins identified and regulated by homeostatic scaling here provides a starting point to examine how dysregulation of these “homeostatic” proteins contributes to a variety of neuronal disorders.

Are the two forms of plasticity, homeostatic up-scaling and down-scaling, on a single mechanistic continuum, or are orthogonal mechanisms used? Most mechanistic studies have focused on up-scaling, examining the role of molecules that are associated with AMPA receptor trafficking or stabilization. A general question is whether the same mechanisms run in reverse for down-scaling. We observed multiple protein families and regulatory pathways that exhibited opposite regulation depending on the sign of the scaling (Figures 4 and S3), suggesting that these two forms of plasticity can be mediated by differential regulation of a common molecular-protein continuum. These protein families include, for example, the ionotropic glutamate receptor complex, proteins associated with neurotransmitter transport and exocytosis, and transcription factors. These data support the idea that at least some of the opposite phenotypes elicited by up- and down-scaling are driven mechanistically by an overlapping cast of molecular players, regulated in different directions. If the global manipulations of activity used here and in other studies possess a common sensor (e.g., Ca^2+^), then the downstream mechanisms that bring about scaling-up or scaling-down could activate Ca^2+^ sensors with different sensitivities (e.g., Sutton et al., 2007) to bring about a concerted protein synthesis response.

A central question for all forms of cellular plasticity, of course, is the degree to which phenotypic changes are driven by changes in transcription and/or translation. A previous study indicated a requirement for transcription in homeostatic up-scaling (Ibata et al., 2008). Here we show for the first time that inhibition of protein synthesis during the initial 12 hr of scaling induction blocks both scaling-up and scaling-down. The period of protein synthesis inhibition was brief relative to the half-life of most neuronal/synaptic proteins (median = ∼3.5 days; Cohen et al., 2013), and there was no effect on baseline mEPSC frequency or amplitude. We thus conclude that the observed inhibition of scaling reflects a specific requirement for newly synthesized proteins in the expression of homeostatic scaling. Whether the observed changes in protein synthesis are due to changes in translation specifically or accompanied by changes in transcript level is an interesting question. In our data, there are several examples where multiple elements of a common signaling cascade are regulated (e.g., Figure 7). An open issue concerns the transcriptional or translational regulatory mechanisms that coordinate the changes in neuronal compartments so that elements in the same signaling pathway are consistently regulated to bring about change at the end-point, e.g., a positive or negative modulation of synaptic strength. In addition, although it has not been explicitly addressed here, it is obvious that the regulated degradation of proteins must also play a role in sculpting the proteome during homeostatic scaling. Indeed, we observed many proteins in the ubiquitin proteasome pathway that were regulated by scaling (Figure 3C; Table S3).

In addition to the proteomic changes described here, protein degradation, protein modifications, and/or transient protein complex formation play critical roles in adjusting synaptic strength. Indeed, Ehlers (2003) identified a cohort of synaptic proteins that were differentially regulated by activity manipulations and noted that inhibition of the ubiquitin proteasome pathway blocked the differential regulation. In addition, fast adaptions, such as short-term potentiation or depression, often rely heavily on modifications of existing proteins. In support of this idea, “kinases/phosphatases” represent the largest differentially regulated group in our dataset (Figure 3C; Table S3). For example, we found multiple MAP kinases and associated proteins (Table S3) that play a central role in multiple cellular processes. Also, our String analysis revealed that the tyrosine-protein kinases Lyn and Fyn represent central interaction “hubs” of the differentially regulated proteins in our dataset. Accordingly, future studies have to focus on obtaining complementary data on post-translational modifications and transient complex formation during homeostatic scaling.

We used BONCAT in hippocampal cultures to discover the nascent proteomic response to both up- and down-scaling. As the non-canonical amino acid AHA can be charged by the wild-type MetRS, all cells present in the culture can, in theory, contribute to the newly synthesized proteins we analyzed. Using bioinformatics, we were able to approximate attributions of some proteins to glia or interneurons, but this type of analysis is clearly limited. For example, it has been shown that homeostatic scaling can also be elicited at inhibitory synapses (Hartman et al., 2006). As such, common proteins in excitatory and inhibitory neurons that exhibit regulation in opposite directions might “cancel each other out” and hence not be detected as significantly regulated in our study. To gain selective access to particular cell classes, a recent development in which a mutant MetRS enables cell-type-specific labeling could be used in future experiments. This has already been employed in both *C. elegans* (Yuet et al., 2015) and *Drosophila* (Erdmann et al., 2015) to monitor protein synthesis in specific cell types. Applying this technology to homeostatic scaling and other forms of plasticity will allow us to discern the coordinated proteomic response in the different cell types that make up neuronal circuits.

## Experimental Procedures

### Preparation and Maintenance of Cultured Neurons

Dissociated hippocampal neurons were prepared and maintained as previously described (Aakalu et al., 2001). Briefly, hippocampi from postnatal day 0–2 rat pups (strain Sprague-Dawley) were dissected out and dissociated by either trypsin or papain and plated at a density of 40,000 cells/cm^2^ onto poly-D-lysine-coated glass-bottom Petri dishes (MatTek). Cultures were maintained in Neurobasal A medium containing B-27 and Glutamax supplements (Invitrogen) at 37°C for 21 days before experiments commenced. All experiments were carried out with the approval of the German animal experiment authorities.

### Electrophysiology

Whole-cell recordings were performed in hippocampal culture neurons (DIV 21–27, 40 K cell density) held at −70 mV in voltage clamp mode. To induce homeostatic plasticity, neurons were pretreated with either bicuculline (40 μM) or TTX (2 μM) for 24 hr. To block protein synthesis, neurons were incubated in anisomycin (40 mM) for the first 12 hr of the treatment. mEPSCs were recorded for at least 10 min in extracellular solution containing 140 mM NaCl, 3 mM KCl, 10 mM HEPES, 2 mM CaCl_2_, 1 mM MgSO_4_, 15 mM glucose, 2 μM TTX, and 40 μM bicuculline (pH 7.4). Recording pipettes (resistances 3–8 MΩ) contained 120 mM potassium gluconate, 20 mM KCl, 10 mM HEPES, 2 mM MgCl_2_, 0.1 mM EGTA, 2 mM Na_2_-ATP, and 0.4 mM Na_2_-GTP (300 mOsm/L, pH 7.2). Data were analyzed offline in MATLAB using a custom-made template-matching algorithm. Selected mEPSC events were individually screened with an amplitude threshold of > 5 pA and an exponential decay. Statistics were conducted using unpaired t tests.

### Sample Preparation and MS Analyses

Dissociated hippocampal neurons (DIV 21, two dishes per condition, ∼800,000 cells in total) were incubated with 4 mM AHA (or methionine for the control group) and treated with bicuculline (20 μM) or tetrodotoxin (1 μM) for 24 hr. After incubation, the cells were washed with cold DPBS containing protease inhibitor cocktails (cOmplete EDTA-free, Roche), harvested, snap-frozen in liquid nitrogen, and stored at −80°C until purification.

For cell lysis, the samples (cell pellets) were centrifuged briefly, the pellets resuspended in lysis buffer (100 μL, 8 M urea, 200 mM Tris [pH 8.4], 4% CHAPS, 1 M NaCl, cOmplete EDTA-free protease inhibitor), and lysed using a pistil. After sonication (4 × 30 s at 4°C), benzonase (1 μL of a ≥ 250 units/μL stock solution) was added to the solubilized samples, incubated for 5 min, and centrifuged for 5 min at 10,000× *g*. The supernatants (∼200 μL) were subsequently incubated with freshly prepared catalyst solution (250 μL) and Alkyne-Sepharose slurry (50 μL; see manufacturer’s protocol, Thermo Fisher Scientific) and incubated for 19 hr in the dark under gentle agitation.

After a short centrifugation (2 min, 1,000× *g*), the beads were washed twice (900 μL each) and incubated with SDS wash buffer (100 mM Tris [pH 8], 1% SDS, 250 mM NaCl, 5 mM EDTA) containing 10 mM TCEP (250 μL). After incubation for 45 min at 55°C under gentle agitation, the samples were centrifuged for 5 min at 1,000× *g* and the supernatants discarded. Each sample was then incubated with 250 μL SDS wash buffer containing a final concentration of 95 mM iodoacetamide for 30 min at room temperature in the dark under gentle agitation.

The samples were then transferred into pre-washed (400 μL H_2_O, LC-MS/MS-ChromaSolv; 400 μL SDS wash buffer) Pierce Spin columns and washed with 20 mL SDS wash buffer, 20 mL 8 M urea in 100 mM Tris (pH 8), and 20 mL 20% (v/v) acetonitrile/water.

After washing, 250 μL of digestion buffer (100 mM Tris, 2 mM CaCl_2_, 10% acetonitrile) was added to each column and the resin transferred to an Eppendorf tube. After one washing step with digestion buffer, ∼0.65 μg of EndoLysC was added to each tube and incubated overnight (∼22 hr) at 37°C with constant agitation. For the second digestion step, each sample was incubated with 0.65 μg trypsin at 37°C overnight (∼22 hr) with constant agitation.

The proteolytic peptides were extracted by incubating the resin with 500 μL H_2_O (0.1% TFA), centrifuging for 5 min at 1,000× *g*, and collecting the supernatant. This step was repeated, and the combined supernatants were desalted using *c*_*18*_-SepPak columns (Waters Corp.). The SepPak resin was washed with 2 mL of 50% acetonitrile/water (5% acetic acid) and equilibrated using 2 mL water (0.1% TFA). Each sample (∼1 mL) was loaded onto a column and washed with 2 mL of water (0.1% TFA) and 200 μL of water (0.5% acetic acid). Peptides were eluted using 0.5 mL of 50% acetonitrile/water (0.5% acetic acid), dried using a Speed-Vac, and stored at −80°C until LC-MS/MS analysis.

For LC-MS/MS injection, the dried peptide fractions were dissolved in 5% acetonitrile with 0.1% formic acid, and subsequently loaded using a nano-HPLC (Dionex U3000 RSLCnano) on reverse-phase columns (trapping column: particle size 3 μm, C18, L = 20 mm; analytical column: particle size < 2 μm, C18, L = 50 cm; PepMap, Dionex/Thermo Fisher Scientific). Peptides were eluted in gradients of water (buffer A: water with 5% [v/v] acetonitrile and 0.1% formic acid) and acetonitrile (buffer B: 20% [v/v] water, 80% [v/v] acetonitrile, and 0.1% formic acid). All LC-MS/MS-grade solvents were purchased from Fluka. Gradients were ramped from 4% to 48% B in 178 min at flowrates of 300 nL/min. Peptides eluting from the column were ionized online using a Thermo nanoFlex ESI source and analyzed in a Thermo “Q Exactive Plus” mass spectrometer. Mass spectra were acquired over the mass range 350–1,400 m/z, and sequence information was acquired by computer-controlled, data-dependent automated switching to MS/MS mode using collision energies based on mass and charge state of the candidate ions (TOP12, MS resolution 70 k, MS^2^ resolution 35 k, injection time: 120 ms, full parameter set in Table S9). All samples were measured in quadruplicate LC-MS/MS runs.

### Bioinformatic Processing and Criteria

Raw data were loaded into MaxQuant (version 1.5.3.8; Cox and Mann, 2008) and analyzed with a customized Andromeda LFQ parameter set (see Table S9). In brief, spectra were matched to a *Rattus norvegicus* database downloaded from uniprot.org (34,170 entries, reviewed and non-reviewed) and a contaminant and decoy database. Precursor mass tolerance was set to 20 ppm, fragment ion tolerance to 4.5 ppm, with fixed modification of Cys residues (carboxyamidomethylation +57.021) and variable modifications of Met residues (Ox +15.995), Lys residues (Acetyl +42.011), Asn and Gln residues (Deamidation +0.984), and N termini (carbamylation +43.006). Peptide identifications were calculated with FDR = 0.01, and proteins with one peptide per protein were included for subsequent analyses.

Five independent biological replicates were processed for each group (methionine control, untreated cells, bicuculline-treated cells, tetrodotoxin-treated cells). After subtraction of background proteins, protein lists for each group were compiled containing proteins identified in at least 2 out of 5 replicates. The methionine content of our AHA-labeled proteins was not different compared to a global proteome (*Rattus norvegicus*, Uniprot database) as, on average, 2.38 and 2.40 met residues are present per 100 amino acids, respectively.

For full details on the data processing, please see Supplemental Experimental Procedures.

## Author Contributions

C.T.S., J.D.L., and E.M.S. designed experiments; C.T.S., S.S., and J.D.L. conducted and analyzed experiments. E.M.S. wrote the manuscript; all authors edited and revised the manuscript.

## Accession Numbers

The proteomics data associated with this manuscript have been uploaded to the PRIDE online repository: PXD004889 (Vizcaíno et al., 2016).

## Supplemental Information

Document S1. Supplemental Experimental Procedures, Figures S1–S5, and Tables S2 (Part B) and S8

Table S1. Table of All Proteins Identified in Each Group

Table S2. Part A, Containing All 307 Significantly Regulated Proteins

Table S3. Identified and Significantly Regulated Proteins According to Functional Groups

Table S4. All Proteins Shown in Figures 5, 6, and 7, with Corresponding Analysis

Table S5. List of Previously Implicated Homeostasis Molecules and Their Detection and Regulation in This Study

Table S6. Newly Synthesized Significantly Regulated Proteins of Potential Glial Origin

Table S7. Newly Synthesized Significantly Regulated Proteins Associated with Some Neurodegenerative, Neuropsychiatric and Neurodevelopmental Disorders

Table S9. Full List of LC-MS/MS and MaxQuant Parameters Used in This Study

Document S2. Article plus Supplemental Information
