# Supplementary material for: Nascent Proteome Remodeling following Homeostatic Scaling at Hippocampal Synapses
Source: Neuron. 2016 Oct 19;92(2):358–71. doi: 10.1016/j.neuron.2016.09.058 (PMC5078608; doi:10.1016/j.neuron.2016.09.058)
Supplement: Document S1. Supplemental Experimental Procedures, Figures S1–S5, and Tables S2 (Part B) and S8 [file mmc1.pdf]

Neuron, Volume *92*

Supplemental Information

Nascent Proteome Remodeling following Homeostatic Scaling at Hippocampal Synapses

Christoph T. Schanzenbächer, Sivakumar Sambandan, Julian D. Langer, and Erin M. Schuman

Schanzenbaecher et al., Supplemental Materials.

Contents:

Supplemental Figures 1-5.
Supplemental Figures 1-5 legends.
Supplemental Table legends.
Supplemental Table 2b.
Supplemental Table 8.
Supplemental Experimental Procedures.

Additional separate files:

Supplemental Table 1.
Supplemental Table 2a.
Supplemental Table 3.
Supplemental Table 4.
Supplemental Table 5.
Supplemental Table 6.
Supplemental Table 7.
Supplemental Table 9.

Supplemental Fig. 1

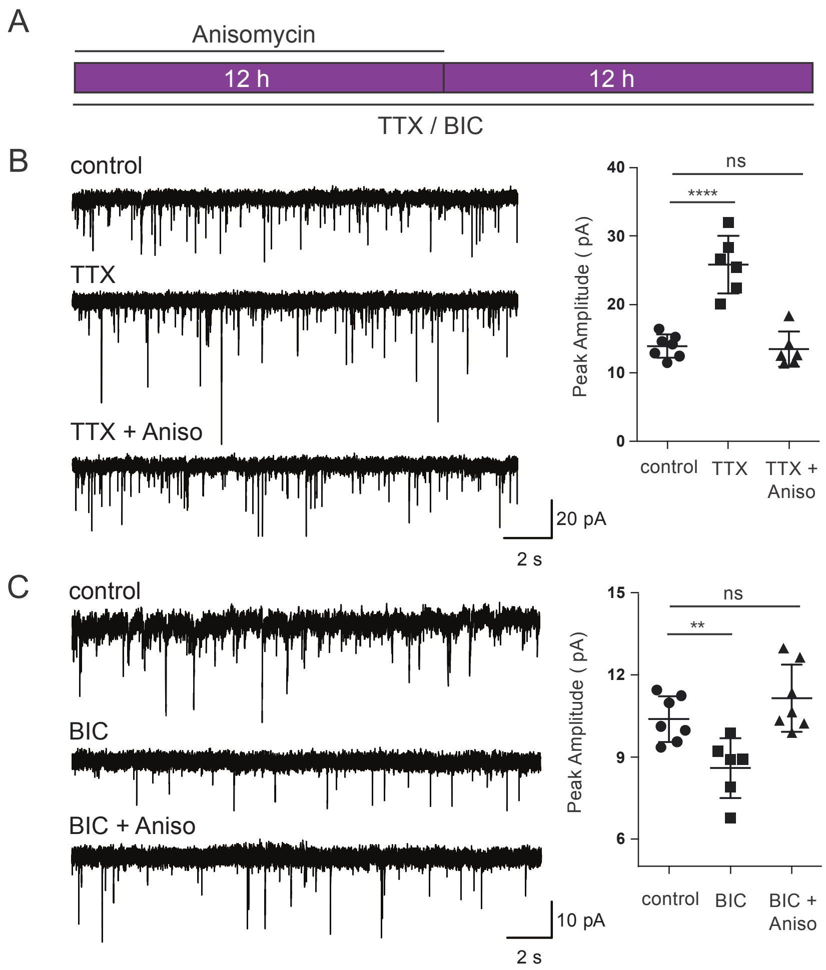

Supplemental Fig. 2

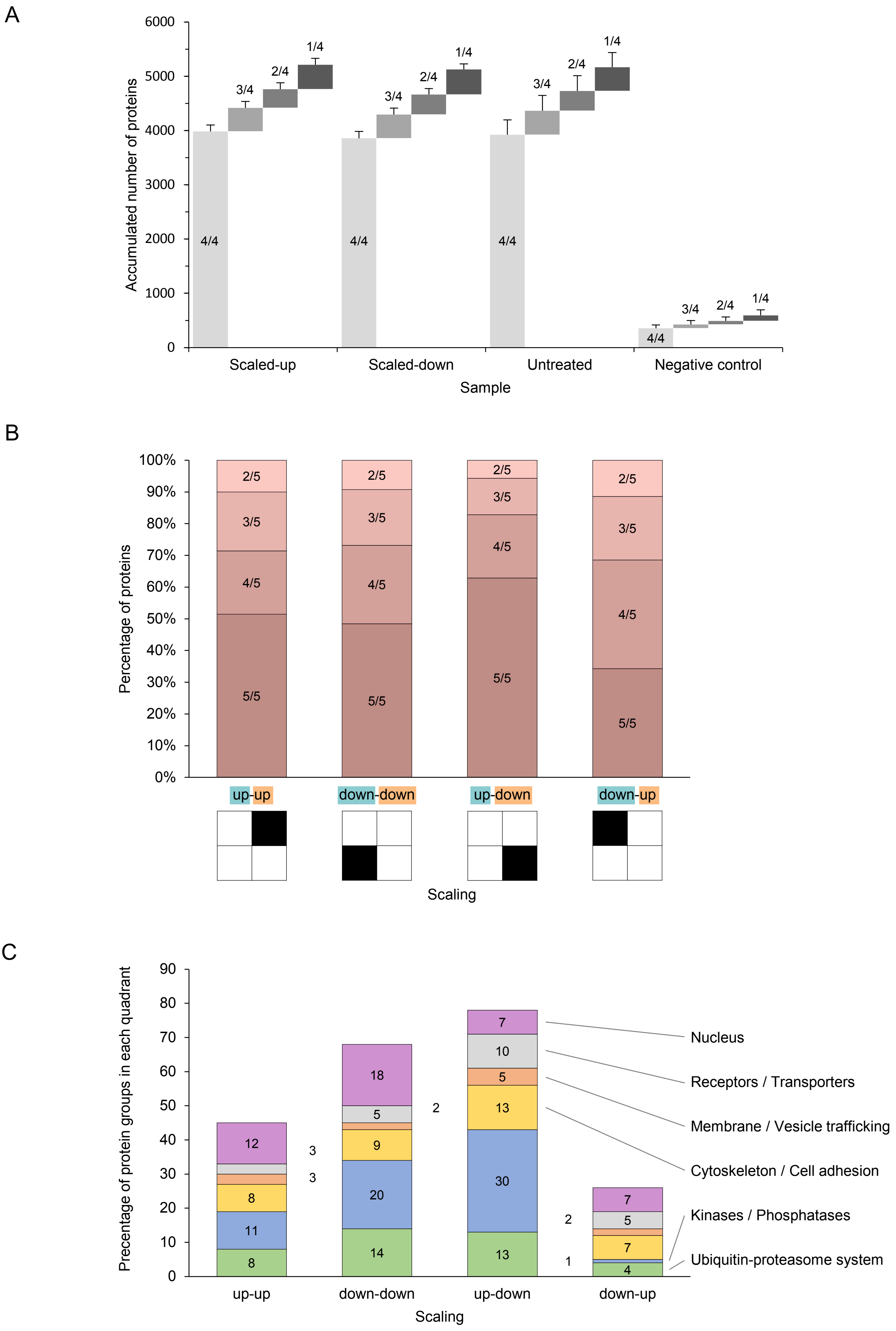

Supplemental Fig. 3

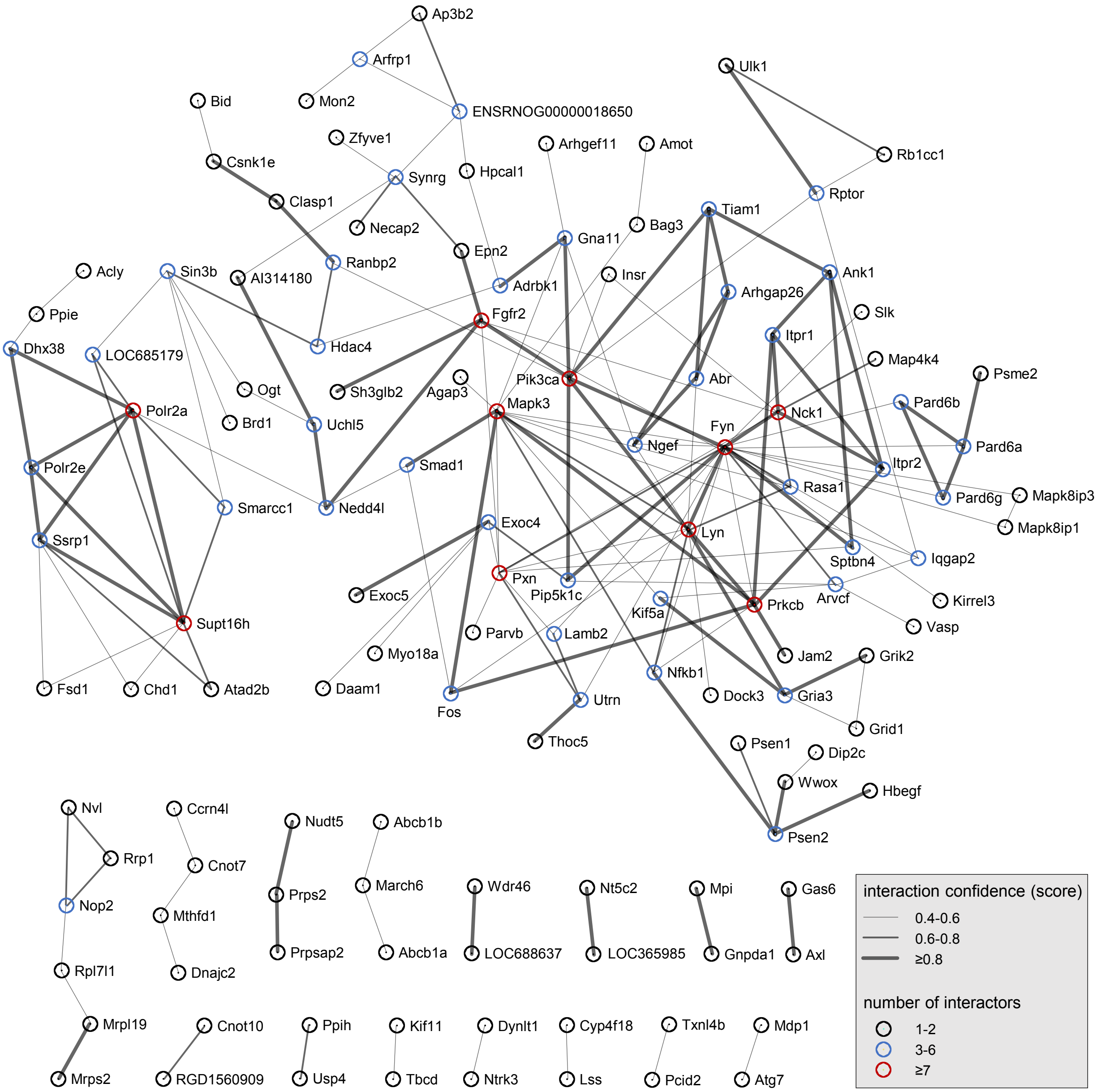

Adap1, Adarb1, Appl2, Arhgap12, Arhgap21, Asns, Atg2b, Atn1, Atpif1, Bcl7a, Birc6, Brsk1, C1ql3, Cacnb4, Cadm3, Caskin1, Ccdc51, Cdh11, Clasrp, Clcn4, Coasy, Commd3, Coro7, Cpne3, Crh, Cript, Ctbs, Cul4b, Dbr1, Ddhd2, Ddx41, Dnajc11, Dnajc13, Dnpep, Dos, Elfn2, ENSRNOG00000049462, Esrra, Esyt1, Etl4, Evl, Fam102a, Fam136a, Fam21c, Fbxl16, Fbxo41, Fbxo42, Fem1a, Flrt3, Fv1, Galnt1, Gcc1, Gdpd1, Ggnbp2, Gripap1, Hars2, Hid1, Hmgb3, Hsd11b1, Igf2r, Kif21b, Kif3c, Kifap3, Lactb, Lemd2, Lpl, Lrig1, Lrrc57, Lrrc7, Lrrc8a, Lrrc8c, Lyplal1, Macf1, Madd, Map4k3, Map7d2, Mdga2, Mms19l, Mospd1, Mtmr1, Nanp, Nckipsd, Ncoa4, ND5, Ndst1, Necab2, Nipa1, Nlgn2, Nol9, Npdc1, Phrf1, Phyhip, Pibf1, Pigq, Pinx1, Pja1, Plekha6, Plod1, Pnpla6, Pogz, Ppm1h, Ppp1r8, Prkar1b, Prune2, Ptpn9, Ptprg, Ptprn2, Qrich1, R3hdm1, Ralb, Rapgef2, Raver1, Rcan2, Rft1, RGD1559896, RGD1560248, Rnh1, Sall2, Samd8, Sbf1, Scai, Scg5, Secisbp2l, Sema6d, Setd3, Slc12a5, Slc25a17, Slc2a13, Slc30a5, Slc39a14, Smpd2, Snap29, Snx29, Sowahc, Srpk1, Stk24, Sugp2, Sympk, Syt6, Taf9b, Tagln3, Tamm41, Tanc2, Tbcel, Tmed5, Tnks1bp1, Tnr, Trabd, Trappc8, Trim3, Tsr2, Tsta3, U2surp, Ubap2, Ubxn4, Ufc1, Urgcp, Usp24, Uxt, Vangl1, Vezt, Wdr47, Wwc3, Wwp2, Zcchc12, Zer1, Zfp131, Zfp36l1, Zfp498, Zfp655

Supplemental Fig. 4

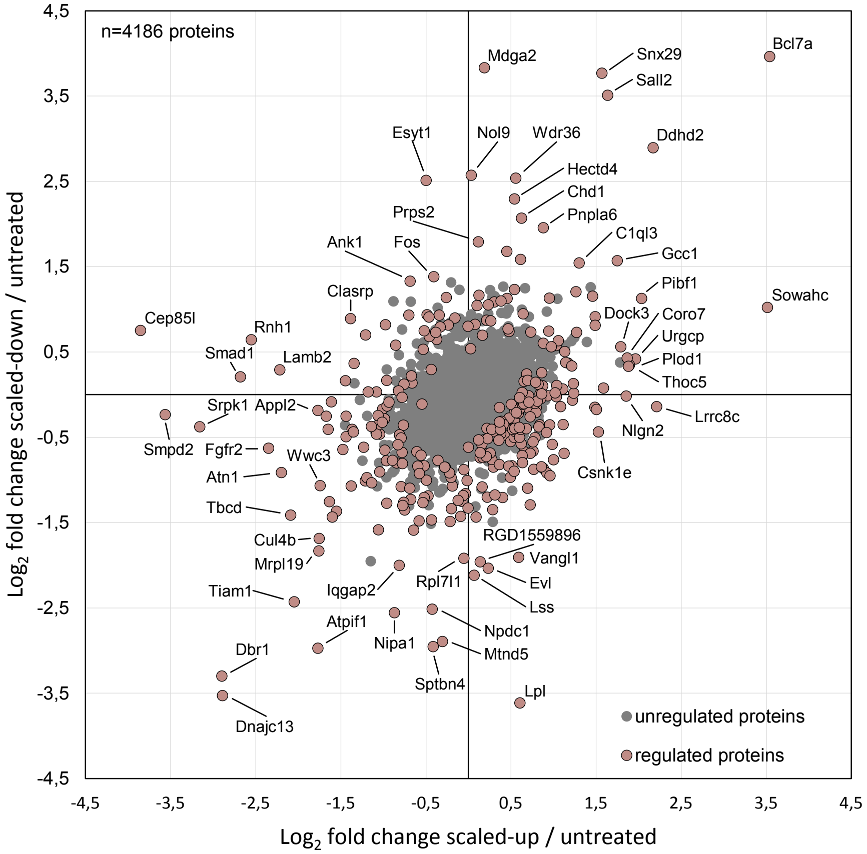

Supplemental Fig. 5

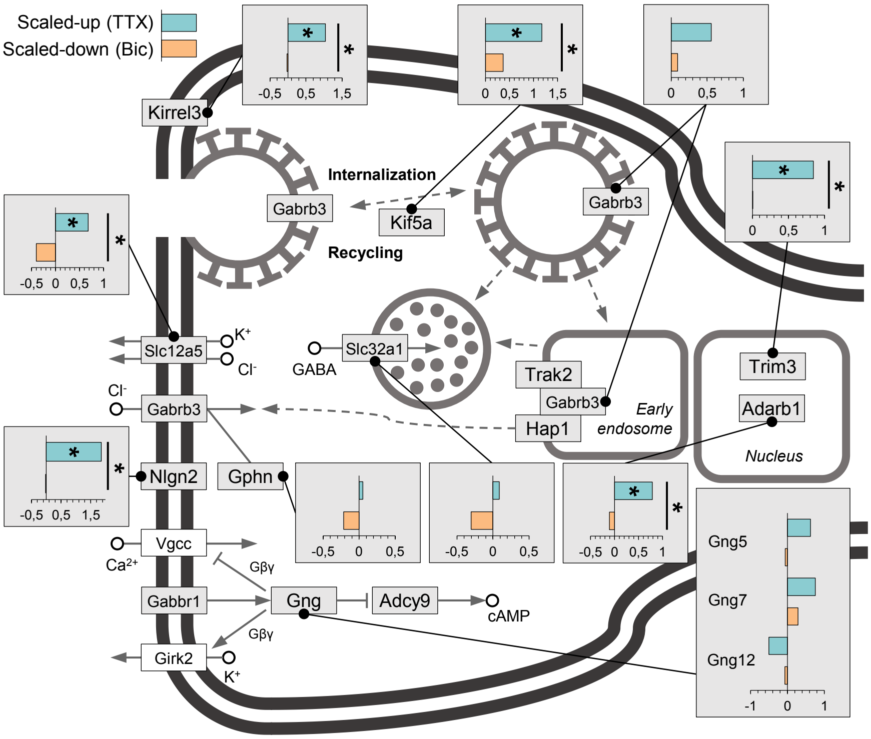

Schanzebaecher et al.,

Supplemental Figures.

Supplemental Figure 1, related to Figure 2. Protein synthesis is required for homeostatic up- and down-scaling.

(A) Scheme indicating experimental work-flow. The protein synthesis inhibitor anisomycin (40 μM) was applied for the first 12 hrs of scaling induction (24 hrs) by either TTX (1 μM) or bicuculline (40 μM).

(B) Representative electrophysiological recordings of miniature excitatory postsynaptic currents (mEPSCs) from control, TTX and TTX + anisomycin experiments. Analysis of mEPSC amplitude for groups, as indicated. TTX-treated neurons exhibited significantly larger mEPSCs than either control ($p < 0.0001$) or TTX + aniso-treated neurons ($p < 0.001$), which did not differ significantly from one another. There was no change in mEPSC frequency produced by TTX treatment (data not shown).

(C) Representative electrophysiological recordings of miniature excitatory postsynaptic currents (mEPSCs) from control, bicuculline and bicuculline + anisomycin experiments.Analysis of mEPSC amplitude for groups, as indicated. Bicuculline-treated neurons exhibited significantly smaller mEPSCs than either control ($p < 0.0066$) or Bicuculline + aniso-treated neurons ($p < 0.0023$), which did not differ significantly from one another. There was no change in mEPSC frequency produced by Bicuculline treatment (data not shown).

Supplemental Figure 2, related to Figures 2 and 3. Reproducibility of the dataset.

(A) Technical replicates for the global sample showing the average reproducibility of the 4 technical replicates that make up each of the 5 biological replicates.

(B) Biological replicate reproducibility of significantly regulated proteins in each of the quadrants of Figure 4A. Quadrants are as indicated and colored labels indicate the condition, scaling-up (teal), scaling-down (yellow) and whether the quadrant represents enhanced (“up”) or reduced (“down”) synthesis.

(C) Protein groups and numbers of proteins represented in the 4 quadrants of Figure 4A.

Supplemental Figure 3, related to Figure 4. Network analysis of all differentially regulated proteins. String pathway analysis showing that many (95) of the regulated proteins can be assembled into a single, large interactive network.

Supplemental Figure 4, related to Figure 4. Scatterplot of significantly regulated proteins showing quadrant localization with labels on the proteins that exhibit high levels of regulation.

Supplemental Figure 5, related to Figures 5 and 6. Proteins associated with inhibitory synaptic transmission regulated by homeostatic scaling. Scheme of an inhibitory synapse. Proteins in grey boxes were detected in our samples, call-out boxes are included for all proteins which fulfilled the criteria for the display of intensity values (see methods). The bars, teal and ochre, represent the regulation of the indicate protein in up- and down-scaling, respectively. Asterisks within the colored bar indicate that the protein exhibited significant (ANOVA FDR < 0.05 and Fisher LSD post-hoc $p < 0.05$) regulation relative to control; asterisks associated with the black lines indicate the protein exhibited significant (ANOVA FDR < 0.05 and Fisher LSD post-hoc $p < 0.05$) regulation between up- and down-scaling.

Supplemental Tables.

Supplemental Table 1 (separate file), related to Figure 2. Table of all proteins identified in each group.

Supplemental Table 2a, (separate file), related to Figure 3. Contains all 307 significantly regulated proteins.

Supplementary Table 2b, related to Figure 3. Low-abundance proteins with trends for enrichment conditions.

Supplemental Table 3, (separate file), related to Figure 3. Identified and significantly regulated proteins according to functional groups.

On different pages (tabs) we list the following protein groups:

a) Ion channels and ion transport

b) Cytoskeletal, cell adhesion and motor proteins

c) Synapse and cell junction

d) Kinases and phosphatases

e) Neurotransmitters and Exocytosis

f) Gene transcription

g) mRNA regulation, protein translation and protein degradation

Supplemental Table 4, (separate file), related to Figures 5-7. All proteins shown in Figures 5-7 with corresponding analysis.

Supplemental Table 5, (separate file), related to Figures 3 and 4. List of previously implicated homeostasis molecules and their detection and regulation in this study.

Supplemental Table 6, (separate file), related to Figures 5-7. Newly synthesized significantly regulated proteins of potential glial origin.

Supplemental Table 7, (separate file), related to Figure 8. Newly synthesized significantly regulated proteins associated with some neurodegenerative, neuropsychiatric and neurodevelopmental disorders.

Supplemental Table 8, related to Figure 3. Full list of protein groups (<50 proteins) found using 1D annotation enrichment (see Materials and Methods). Proteins are sorted by first column (mean log_2 fold change Bic/TTX), table also includes Median log2 fold change (Bic/TTX), group annotation name, group identifier, the number of proteins in the group, P value for 1D enrichment, Benjamini-Hochberg FDR and annotation database the group term derives from (GOCC = Gene Ontology Cellular Component, GOMF = Gene Ontology Molecular Function, GOBP = Gene Ontology Biological Processes, EMBL-EBI databases Pfam (Protein Families) and InterPro).

Supplemental Table 9, (separate file), related to Figure 1. Full list of LC-MS and MaxQuant parameters used in this study.

Supplemental Table S2b

Scaled up (TTX)

Protein ID(s)	Gene symbol(s)	Peptide ratio Bic/Untd	Peptide ratio TTX/Untd	Peptide ratio Bic/TTX	Log2 fold change Bic-Untd (±pooled standard error)	Log2 fold change TTX-Untd (±pooled standard error)	Log2 fold change Bic-TTX (±pooled standard error)	Bic: number of biological replicates	TTX: number of biological replicates	Untd: number of biological replicates	Bic: mean unique peptides	TTX: mean unique peptides	Untd: mean unique peptides	Bic: SD unique peptides	TTX: SD unique peptides	Untd: SD unique peptides	Bic: mean Log2 LFQ	TTX: mean Log2 LFQ	Untd: mean Log2 LFQ	Bic: coefficient of variation	TTX: coefficient of variation	Untd: coefficient of variation	Number of proteins	Mol. weight [kDa]	Sequence length	Score
P51400, G3V649	Adarb1	0.52	0.63	0.39	-0.106±0.252	0.786±0.204	-0.892±0.206	5	5	5	2.6	4.1	2.4	1.1	1.5	0.9	23.483	24.375	23.589	0.32	0.171	0.316	2	77.924	711	47.953
F1LV12, P26769	Adcy2	0.36	0.39	0.47	-	-	-	1	3	1	1	1.1	1.8	-	0.2	-	-	-	-	-	-	-	9	96.385	852	5.1394
P26817, F1LMA4	Adrbk1	0.42	0.64	0.29	-0.955±0.119	0.964±0.138	-1.919±0.175	5	5	5	1.2	3	1.7	0.4	1.1	0.8	21.923	23.842	22.878	0.193	0.229	0.058	3	79.784	689	6.9782
M0R7U1	Ak5	0.44	0.61	0.34	-0.848±0.369	0.861±0.455	-1.709±0.352	5	5	5	1.3	2.6	1.7	0.4	1.2	1.1	22.281	23.989	23.128	0.181	0.583	0.621	1	63.155	562	9.2167
F1MAK3, B5DEI5	Arhgap32, Rics	0.52	0.63	0.39	-0.237±0.494	0.699±0.463	-0.936±0.425	5	5	5	3.7	5.7	3.4	1.4	1	1.4	24.087	25.023	24.324	0.653	0.53	0.783	2	229.58	2086	56.028
B4F7F3	Arvcf	0.49	0.62	0.37	0.631±0.162	1.062±0.177	-0.43±0.16	4	5	4	1.5	2.6	1.6	0.4	1.4	0.4	21.801	22.231	21.169	0.146	0.203	0.196	1	105.49	973	22.025
Q64568-10, Q64568-	Atp2b3	0.5	0.5	0.5	-	-	-	1	3	1	1	1	1	-	0	-	-	22.662	-	-	0.265	-	6	126.82	1154	275.64
F1LXF1	Bcr	0.5	0.63	0.38	-	-	-	1	3	3	1	1.7	1	-	0.6	0	-	-	-	-	-	-	1	95.699	842	3.191
F1LSR8, Q8CFG5	Cacna2d3	0.47	0.6	0.38	-0.3±0.626	0.827±0.532	-1.127±0.51	2	4	3	1.1	1.9	1.3	0.2	1	0.4	21.558	22.685	21.858	0.35	0.55	0.72	2	121.94	1082	4.3118
F1LQD2, P97756	Camkk1	0.34	0.61	0.24	-	1.363±0.512	-	4	5	4	1.1	3.4	2.1	0.2	2	0.9	-	24.205	22.842	-	0.988	0.167	2	55.897	505	16.501
M0R7N7, M0R577, I	Caprin2	0.5	0.63	0.38	-	-	-	1	3	2	1	1.7	1	-	1.2	0	-	24.466	-	-	0.241	-	3	34.922	300	3.2209
D3ZG85, E2E1S0	Cdkl5	0.5	0.61	0.39	-0.361±0.572	0.43±0.674	-0.791±0.65	5	5	5	2.5	3.8	2.5	1.6	2.7	1.6	23.221	24.012	23.582	0.866	1.207	0.721	2	105.21	934	35.472
P97836, G3V849	Dlgap1	0.57	0.67	0.39	-0.757±0.566	0.316±0.421	-1.073±0.497	5	5	5	2.1	3.3	1.6	0.5	1.4	0.5	24.298	25.371	25.055	0.927	0.498	0.606	2	110.18	992	91.774
F1M3D2, M0RDT6	Dmwd	-	1	0	-	-	-	0	3	0	0	1.1	0	-	0.2	-	-	-	-	-	-	-	2	55.868	522	9.6649
F1M4N6	Dock3	0.48	0.64	0.34	0.557±0.639	1.793±0.432	-1.236±0.381	4	5	3	1	2	1.1	0	0.6	0.1	21.22	22.456	20.663	0.459	0.318	0.649	1	233.35	2030	22.554
G3V7D4, A5HTT2	Efnb3	0.48	0.6	0.38	0.038±0.597	0.854±0.554	-0.816±0.383	5	5	5	4.3	7.1	4.7	2.2	1.9	2.6	25.622	26.438	25.584	0.626	0.406	1.198	2	35.792	340	55.294
D3ZH36	Elfn2	0.46	0.62	0.35	-0.299±0.159	0.546±0.224	-0.845±0.148	5	4	4	1.6	3.1	1.9	0.4	1	0.5	23.125	23.969	23.423	0.093	0.233	0.258	1	89.846	820	38.94
F1MAJ0, B2B9B0	Ephb2	0.45	0.62	0.34	0.668±0.577	0.871±0.644	-0.203±0.639	5	5	5	1.5	3	1.9	0.5	2.3	1.2	23.828	24.031	23.16	0.082	1.097	0.952	2	107.47	963	31.288
D4A3K7	Frmpd4	0.47	0.61	0.36	-	-	-0.108±0.773	2	3	2	1.3	2.3	1.5	0.5	0.9	0.2	22.427	22.535	-	0.344	0.989	-	1	187.09	1705	18.053
G3V9C5, Q00959	Grin2a	0	0.42	0	-	-	-	0	4	1	0	1.3	1.8	-	0.4	-	-	22.826	-	-	0.279	-	7	165.48	1464	5.0079
E9PTD7, Q09GS6	Kirrel3	0.55	0.67	0.38	-0.026±0.244	1.037±0.288	-1.064±0.308	5	5	5	2	3.3	1.6	1	1.9	1.3	22.883	23.947	22.91	0.339	0.458	0.271	2	79.355	725	30.003
D3ZBH5	Lmtk2	0.5	0.55	0.45	-	-	-	1	3	1	1	1.2	1	-	0.4	-	-	21.9	-	-	0.144	-	1	165.33	1531	5.1098
D4A1J8, D4A4L4, W	Map7d2	0.47	0.61	0.37	-0.88±0.176	0.773±0.181	-1.653±0.171	5	5	5	1.5	2.5	1.6	0.4	1	0.8	23.012	24.664	23.892	0.201	0.212	0.197	3	66.185	594	17.317
D3ZN55	Ncor1	0.5	0.63	0.38	-	-	-	1	3	3	1	1.7	1	-	0.3	0	-	21.856	-	-	0.218	-	3	144.96	1336	2.3076
P70600-3, P70600	Ptk2b	0.4	0.64	0.27	-0.259±0.58	1.283±0.796	-1.542±0.77	5	5	5	2.1	5.6	3.2	1.7	3.5	2.4	23.02	24.562	23.279	0.815	1.814	0.96	3	111.09	967	49.921
Q9WV48-3, Q9WV4	Shank1	0.48	0.63	0.35	0.642±0.667	1.77±0.686	-1.128±0.623	4	5	4	2	3.7	2.2	2	3	2.1	23.152	24.281	22.511	0.525	0.903	1.016	6	225.47	2158	79.274
G3V8N8, Q8CIV0	Slc7a6os	0.52	0.63	0.39	-	-	-	5	3	3	1.1	1.7	1	0.2	0.9	0	-	22.923	-	-	0.305	-	2	35.032	305	19.073
F1LTE0	Tanc2	0.42	0.64	0.3	-0.587±0.499	0.989±0.467	-1.576±0.445	5	5	5	3.2	7.6	4.3	1.4	3	2.4	24.176	25.752	24.763	0.689	0.565	0.767	1	219.81	1992	36.474
D3ZTN1	Tesc	0.54	0.66	0.37	-0.44±0.353	0.834±0.375	-1.274±0.337	2	3	3	1.5	2.6	1.3	0.3	1.1	0.6	22.919	24.193	23.359	0.064	0.364	0.385	1	24.599	214	30.564
A0A096MK46, Q9R	Timm10b	0.5	0.6	0.39	-0.337±0.311	0.52±0.292	-0.856±0.302	5	4	5	1.1	1.6	1.1	0.1	0.3	0.1	23.146	24.002	23.483	0.331	0.249	0.35	3	11.657	100	6.4449
F1M0V0	Zfp280d	0.55	0.67	0.37	-	-	-	4	3	2	1.4	2.3	1.1	0.5	0.6	0.2	22.857	-	-	0.269	-	-	2	107.28	969	23.902

Scaled down (Bic)

Protein ID(s)	Gene symbol(s)	Peptide ratio Bic/Untd	Peptide ratio TTX/Untd	Peptide ratio Bic/TTX	Log2 fold change Bic-Untd (±pooled standard error)	Log2 fold change TTX-Untd (±pooled standard error)	Log2 fold change Bic-TTX (±pooled standard error)	Bic: number of biological replicates	TTX: number of biological replicates	Untd: number of biological replicates	Bic: mean unique peptides	TTX: mean unique peptides	Untd: mean unique peptides	Bic: SD unique peptides	TTX: SD unique peptides	Untd: SD unique peptides	Bic: mean Log2 LFQ	TTX: mean Log2 LFQ	Untd: mean Log2 LFQ	Bic: coefficient of variation	TTX: coefficient of variation	Untd: coefficient of variation	Number of proteins	Mol. weight [kDa]	Sequence length	Score
F1M400	Ankrd28	1	-	1	-	-	-	3	0	0	1	0	0	0	-	-	-	-	-	-	-	-	1	112.96	1053	3.3067
D4A9G6	Arhgap33	0.56	0.5	0.56	-	-	-	4	1	1	1.3	1	1	0.5	-	-	22.994	-	-	0.204	-	-	1	137.39	1284	3.1981
O08560	Dgkz	0.57	0.6	0.47	-	-	-	3	1	1	1.3	1.5	1	0.6	-	-	22.667	-	-	0.199	-	-	1	104.01	929	2.0479
P12841, Q63752, Q5	Fos, c-fos	0.63	0.45	0.67	1.378±0.365	-0.401±0.319	1.779±0.348	5	5	5	2.6	1.2	1.5	1.6	0.3	0.6	24.387	22.608	23.009	0.535	0.389	0.447	4	40.926	380	109.69
Q9EQH1, F1LSR2	Gab2	0.6	0.5	0.6	0.288±0.287	0.201±0.28	0.087±0.302	4	4	5	1.5	1	1	0.4	0	0	21.722	21.634	21.434	0.31	0.132	0.277	2	73.327	665	3.4289
D3ZKF5, A0A0A0M	Htra4	0.68	0.46	0.71	1.628±1.264	-0.316±0.667	1.944±0.996	5	4	3	5.1	2.1	2.4	3.3	0.9	1.8	24.947	23.003	23.319	2.791	0.377	1.395	2	52.192	488	151.84
D3ZVG3, P70581-4,	Nupl1	0.63	0.52	0.62	0.179±0.228	-0.083±0.184	0.262±0.22	5	5	5	2.3	1.4	1.3	1.7	0.3	0.2	23.558	23.297	23.38	0.302	0.183	0.211	5	51.317	507	19.44
D3ZK25	RGD1309748	1	-	1	-	-	-	3	0	0	1.1	0	0	0.1	-	-	-	-	-	-	-	-	2	30.939	275	1.6071
Q6AZ64, Q8VIB1, E	Xrcc6	0.63	0.5	0.63	0.281±0.255	0.04±0.395	0.241±0.347	4	4	5	1.7	1	1	1.2	0	0	24.01	23.769	23.728	0.157	0.477	0.31	4	69.486	608	5.286

Scaled down (Bic) + Scaled up (TTX)

Protein ID(s)	Gene symbol(s)	Peptide ratio Bic/Untd	Peptide ratio TTX/Untd	Peptide ratio Bic/TTX	Log2 fold change Bic-Untd (±pooled standard error)	Log2 fold change TTX-Untd (±pooled standard error)	Log2 fold change Bic-TTX (±pooled standard error)	Bic: number of biological replicates	TTX: number of biological replicates	Untd: number of biological replicates	Bic: mean unique peptides	TTX: mean unique peptides	Untd: mean unique peptides	Bic: SD unique peptides	TTX: SD unique peptides	Untd: SD unique peptides	Bic: mean Log2 LFQ	TTX: mean Log2 LFQ	Untd: mean Log2 LFQ	Bic: coefficient of variation	TTX: coefficient of variation	Untd: coefficient of variation	Number of proteins	Mol. weight [kDa]	Sequence length	Score
O88881-2, O88881	Begain	0.63	0.6	0.53	0.254±0.282	-0.046±0.313	0.301±0.326	4	4	4	2.5	2.3	1.5	1.8	1.8	1	24.744	24.443	24.49	0.299	0.338	0.024	2	66.161	605	46.057
Q9ET45	Bnip3	0.6	0.63	0.47	0.489±0.549	0.595±0.467	-0.106±0.607	5	5	4	1.6	1.8	1.1	0.6	1.1	0.1	22.887	22.993	22.398	0.993	0.777	0.379	2	20.978	187	9.5344
D4A3T5	C1ql3	0.64	0.66	0.48	1.54±0.439	1.306±0.316	0.233±0.402	4	3	3	2.4	2.6	1.3	1.2	0.9	0.6	24.698	24.464	23.158	0.5	0.34	0.039	1	26.687	255	62.802
P13109, A0A2U1	Fgf2	0.5	0.5	0.5	-	-	-	3	4	1	1	1	1	0	0	-	-	-	-	-	-	-	2	17.138	154	1.8796
Q80YG1, E9PSV5, C	Psat1	0.65	0.6	0.55	0.49±1.019	0.572±0.199	-0.082±0.91	5	5	5	3.2	2.6	1.7	3.2	1.1	0.5	23.287	23.369	22.797	3.008	0.284	0.132	3	40.588	370	23.396
B1VKB4, Q9Z327-3,	Synpo	0.6	0.65	0.45	1.218±0.686	1.146±0.734	0.073±0.651	5	5	5	4.6	5.7	3.1	3.3	3.3	2.2	25.475	25.402	24.256	0.648	1.176	1.296	4	96.717	905	109.71

Untd

Protein ID(s)	Gene symbol(s)	Peptide ratio Bic/Untd	Peptide ratio TTX/Untd	Peptide ratio Bic/TTX	Log2 fold change Bic-Untd (±pooled standard error)	Log2 fold change TTX-Untd (±pooled standard error)	Log2 fold change Bic-TTX (±pooled standard error)	Bic: number of biological replicates	TTX: number of biological replicates	Untd: number of biological replicates	Bic: mean unique peptides	TTX: mean unique peptides	Untd: mean unique peptides	Bic: SD unique peptides	TTX: SD unique peptides	Untd: SD unique peptides	Bic: mean Log2 LFQ	TTX: mean Log2 LFQ	Untd: mean Log2 LFQ	Bic: coefficient of variation	TTX: coefficient of variation	Untd: coefficient of variation	Number of proteins	Mol. weight [kDa]	Sequence length	Score
Q5FVP8	Dgat2	0	0	-	-	-	-	0	0	3	0	0	1.2	-	-	0.4	-	-	-	-	-	-	1	43.794	388	4.6894
Q4V890	Fem1a	0.35	0.37	0.48	-0.772±0.228	-0.887±0.188	0.115±0.191	5	5	4	1.1	1.1	1.9	0.1	0.3	1.1	21.077	20.962	21.85	0.253	0.147	0.247	1	72.117	654	18.05
B2GV98	Lrch1	0.36	0.37	0.5	-	-	-	5	3	4	1.1	1.1	1.9	0.1	0.1	0.6	-	-	23.534	-	-	0.158	1	75.211	677	6.3225
F1LPM4, P19643	Maob	0.39	0.36	0.54	-0.547±0.5	-	-	4	3	5	1.3	1.1	2	0.4	0.1	1.5	23.877	-	24.424	0.313	-	0.545	3	56.94	504	18.22
F1M587, F1M1F1	March6	0.38	0.39	0.49	-0.908±0.332	-1.04±0.181	0.132±0.283	5	5	4	1.1	1.1	1.8	0.1	0.2	1.2	22.165	22.033	23.073	0.501	0.174	0.243	2	99.256	883	8.5965
F1M5N4	Me3	0.35	0.4	0.44	-	-	-	2	3	3	1	1.3	1.9	0	0.3	0.8	-	-	23.143	-	-	0.117	1	67.208	605	20.174
C6JUM5	Pcdhgc5	0.4	0.37	0.53	-	-0.661±0.431	-	5	5	5	1.9	1.7	2.8	1	0.8	2	-	22.887	23.548	-	0.477	0.481	1	102.1	944	112.73
F1LQM9	Xpo7	0.39	0.36	0.53	-0.374±0.207	-0.622±0.188	0.248±0.19	5	4	3	1.2	1.1	1.9	0.3	0.1	0.2	21.032	20.784	21.407	0.194	0.005	0.191	2	124.18	1090	15.854

Scaled up (TTX) + Untd

Protein ID(s)	Gene symbol(s)	Peptide ratio Bic/Untd	Peptide ratio TTX/Untd	Peptide ratio Bic/TTX	Log2 fold change Bic-Untd (±pooled standard error)	Log2 fold change TTX-Untd (±pooled standard error)	Log2 fold change Bic-TTX (±pooled standard error)	Bic: number of biological replicates	TTX: number of biological replicates	Untd: number of biological replicates	Bic: mean unique peptides	TTX: mean unique peptides	Untd: mean unique peptides	Bic: SD unique peptides	TTX: SD unique peptides	Untd: SD unique peptides	Bic: mean Log2 LFQ	TTX: mean Log2 LFQ	Untd: mean Log2 LFQ	Bic: coefficient of variation	TTX: coefficient of variation	Untd: coefficient of variation	Number of proteins	Mol. weight [kDa]	Sequence length	Score
Q8CF82, M0RA83, (	Abca5	0	0.5	0	-	0.627±0.231	-	0	3	3	0	1.3	1.3	-	0.6	0.6	-	21.852	21.225	-	0.238	0.036	3	185.81	1642	7.6809
D3ZRJ0	Adck1	0	0.5	0	-	-0.153±0.284	-	0	4	3	0	1	1	-	0	0	-	20.29	20.444	-	0.24	0.194	1	51.71	455	1.5998
Q5BJQ0	Adck3	0.38	0.58	0.31	-0.562±0.363	0.448±0.406	-1.01±0.516	5	5	4	1.1	2.4	1.8	0.2	1.1	0.4	22.525	23.535	23.087	0.109	0.608	0.391	1	72.225	649	19.662
A0A096MJB0	Adcy9	0.38	0.54	0.34	-0.916±0.401	0.139±0.45	-1.055±0.467	4	4	3	1.2	2.3	1.9	0.2	1.3	0.6	22.255	23.31	23.171	0.53	0.63	0.275	2	151	1354	16.431
Q8CGU4	Agap2	0.33	0.54	0.29	-0.193±0.553	0.301±0.524	-0.493±0.593	2	4	3	1.2	2.9	2.4	0.2	2.5	2.5	23.085	23.578	23.278	0.473	0.65	0.546	1	124.44	1186	27.334
F1M446	AI314180	0.4	0.53	0.38	-0.709±0.126	0.227±0.101	-0.936±0.122	5	5	5	3.9	6.5	5.9	0.6	1.3	2.7	22.847	23.784	23.556	0.169	0.11	0.124	2	203.66	1838	39.529
Q9WUS0	Ak4	0.4	0.52	0.38	-0.59±0.475	0.592±0.443	-1.181±0.274	5	5	5	2	3.3	3.1	1.3	1.1	1.5	24.214	25.396	24.804	0.425	0.265	0.905	1	25.203	223	64.079
D4A205	Apc2	0.35	0.47	0.38	-	-0.09±0.885	-	2	5	3	1.2	1.9	2.2	0.2	1.6	2	-	23.396	23.486	-	0.982	1.47	1	243.93	2281	14.403
B2DD29	Brsk1	0.4	0.54	0.36	-0.866±0.248	0.886±0.172	-1.753±0.208	4	4	3	1.7	3	2.6	0.7	0.9	1.1	21.723	23.475	22.589	0.272	0.154	0.19	1	85.182	778	80.707
Q9ERS3, F7F134, D3	Cacna2d1	0.35	0.54	0.31	-0.59±0.523	-0.004±0.857	-0.586±0.739	5	5	4	2	4.4	3.6	1.4	3	3.2	23.701	24.287	24.29	0.19	1.717	1.022	6	123.47	1091	77.115
D3ZLT0, P55215-2, I	Casp2	0	0.5	0	-	-	-	0	3	3	0	1	1	-	0	0	-	-	-	-	-	-	3	38.043	343	1.5126
G3V8P3, Q9QYP2	Celsr2	0.4	0.53	0.37	-0.719±0.272	0.178±0.308	-0.897±0.205	5	5	5	2.5	4.2	3.7	1.6	2.7	2.1	23.282	24.179	24.001	0.172	0.317	0.478	2	317.12	2919	78.803
Q9QX19, E9PSL7	Cit	0.36	0.54	0.32	-0.637±0.618	0.41±0.62	-1.047±0.334	4	4	3	2.8	5.9	5	1.5	2.4	3.7	23.579	24.626	24.215	0.383	0.391	1.21	2	185.92	1618	67.634
H1UBM7, F1M1L9	Cpne2	0.4	0.52	0.38	-0.145±0.303	0.479±0.215	-0.624±0.184	4	4	4	1.1	1.9	1.7	0.3	0.2	0.6	22.821	23.444	22.966	0.286	0.07	0.336	2	61.121	548	18.708
F1LN46	Cpt1c	0.31	0.44	0.37	-1.135±0.844	0.106±0.634	-1.24±0.409	4	5	4	1.3	2.2	2.8	0.4	1.4	2.2	21.579	22.819	22.714	0.556	0.431	1.487	2	90.169	801	64.585
Q45NC2, E9PT92	Csmd1	0.36	0.47	0.38	-	0.217±0.235	-	4	5	3	1.1	1.8	2	0.3	0.9	0.6	-	22.366	22.149	-	0.228	0.289	4	387.61	3564	14.824
F1LQE1, Q6P730	Dab2ip	0.39	0.54	0.35	-0.241±0.211	0.04±0.152	-0.281±0.158	5	5	5	1.1	2.1	1.8	0.1	0.6	0.3	23.429	23.71	23.67	0.266	0.069	0.254	2	126.76	1148	36.718
D3ZT20	Fbxo41	0.39	0.53	0.36	-1.101±0.564	0.7±0.469	-1.801±0.317	4	4	3	1.1	1.9	1.7	0.1	0.9	1.2	20.887	22.688	21.988	0.015	0.34	0.738	1	93.854	870	20.498
B1H234	Flrt3	0.31	0.51	0.3	-1.435±0.17	0.094±0.134	-1.529±0.192	5	4	4	1.2	2.6	2.6	0.2	1.3	0.8	22.705	24.235	24.141	0.239	0.171	0.104	2	72.91	649	5.8944
F1LPQ7, Q5U4E6	Golga4	0.29	0.41	0.37	-1.536±0.729	-0.806±0.458	-0.73±0.644	5	5	5	1.3	2.2	3.2	0.3	0.8	2.2	22.185	22.916	23.721	1.318	0.347	0.753	3	260.27	2259	56.563
G3V6A8, Q63714	Golgb1	0.24	0.46	0.27	-1.66±1.043	0.171±1.149	-1.83±0.792	5	5	5	1.8	4.9	5.7	0.6	6.1	6.5	22.053	23.884	23.713	0.15	1.931	3.138	2	364.25	3187	165.31
D3ZAP9	Gpd1l	0.35	0.52	0.32	-0.484±0.292	0.465±0.23	-0.949±0.253	4	4	4	2.3	4.7	4.3	0.7	0.8	0.4	25.245	26.194	25.729	0.355	0.191	0.306	1	38.066	351	69.156
A0A0A0MY13, D4A	Gpr158	0.24	0.43	0.3	-1.47±0.772	-0.29±0.915	-1.18±0.457	4	5	3	1.3	3.2	4.1	0.5	2.2	3.8	23.204	24.384	24.674	0.206	0.831	1.956	2	134.83	1206	76.474
G3V746, Q00960	Grin2b	0.33	0.5	0.33	-0.989±0.582	-0.509±0.572	-0.48±0.468	4	5	3	1.3	2.7	2.7	0.3	1.9	2.3	22.655	23.135	23.644	0.366	0.645	0.868	2	166.03	1482	55.339

D4A0C3	Hid1	0.39	0.52	0.38	-0.974±0.341	0.497±0.279	-1.471±0.277	5	5	5	1.2	2	1.9	0.3	0.5	0.7	21.188	22.659	22.162	0.089	0.285	0.368	1	88.753	788	10.696
G3V9C1, Q99MG9-2	Kcnip4	0.39	0.52	0.37	-	0.188±0.302	-	2	5	3	1	1.7	1.6	0	0.7	0.1	-	22.82	22.632	-	0.419	0.052	3	24.589	213	6.5055
F1M5N7	Kif21b	0.37	0.56	0.31	-0.691±0.33	0.731±0.388	-1.422±0.231	5	5	5	2.1	4.6	3.6	0.6	1.4	2.3	23.214	24.636	23.905	0.127	0.402	0.646	1	182.56	1634	120.12
M0RC60, D3ZNV6	LOC100910164, Elm	0.39	0.51	0.38	-0.576±0.107	-0.134±0.154	-0.442±0.176	5	5	5	1.5	2.4	2.3	0.3	1.1	0.6	23.404	23.846	23.98	0.162	0.256	0.072	2	34.792	293	7.9191
P21708, P21708-2	Mapk3	0.38	0.54	0.34	-1.177±0.151	0.315±0.152	-1.492±0.177	5	5	5	2.3	4.5	3.8	1	0.4	0.6	23.506	24.999	24.684	0.212	0.216	0.142	4	43.08	380	10.306
Q6AY81	Npdc1	0.4	0.53	0.37	-2.517±0.637	-0.422±0.404	-2.096±0.261	3	5	3	1	1.7	1.5	0	0.5	0.5	21.61	23.706	24.127	0.193	0.252	0.82	1	35.516	331	7.9218
D3ZQD3	Ogdhl	0.39	0.51	0.38	-0.627±0.44	0.589±0.474	-1.217±0.284	4	5	4	1.6	2.7	2.6	0.7	1.9	1.8	21.929	23.146	22.556	0.089	0.465	0.83	1	116.71	1029	22.886
P53817	Pla2g16	0.36	0.48	0.38	-0.579±0.371	-0.405±0.263	-0.175±0.356	5	5	5	1	1.6	1.8	0	0.3	0.4	23.728	23.903	24.308	0.623	0.302	0.365	1	17.748	160	8.1993
M0RDL0, A0A096M	Prdm16	0	0.5	0	-	-	-	0	5	5	0	1	1	-	0	0	-	-	-	-	-	-	4	110.18	992	1.5268
P63319	Prkcg	0.31	0.44	0.37	-	0.478±0.917	-	2	4	3	1.6	2.8	3.6	0.2	2.3	2.8	-	24.472	23.994	-	0.634	2.222	1	78.357	697	31.548
D4A9C3, D4A404, D	Psd3	0.34	0.52	0.32	-0.975±0.572	0.4±0.569	-1.375±0.504	4	4	4	3.1	6.5	5.9	1.3	3.8	3.5	24.799	26.173	25.774	0.643	0.633	0.855	7	42.285	376	33.942
F1M6Y5, G3V953	Rbbp6	0.36	0.5	0.36	-0.511±0.492	0.102±0.555	-0.613±0.288	5	5	5	1.4	2.5	2.5	0.4	0.8	1.6	23.094	23.707	23.605	0.153	0.526	1.115	3	195.57	1755	152.27
F1M7Z9, Q8CIX1	Rims4	0.37	0.53	0.35	-0.61±0.426	0.663±0.317	-1.273±0.23	3	4	4	1.1	2	1.8	0.1	0.3	0.9	21.617	22.89	22.227	0.141	0.219	0.47	2	29.329	269	33.336
Q6RJR6	Rtn3	0.37	0.5	0.37	-1.109±0.509	0.154±0.553	-1.262±0.746	3	5	4	1.7	2.8	2.8	0.8	2.5	1.9	22.414	23.676	23.522	0.46	0.944	0.517	1	101.52	940	60.186
B2GUY8	Slc25a17	0.4	0.52	0.38	-1.207±0.226	0.408±0.275	-1.615±0.275	5	5	5	1.1	1.8	1.6	0.1	0.4	0.5	21.693	23.308	22.9	0.281	0.415	0.28	1	34.34	307	3.5625
F1LSL8	Sptbn4	0.37	0.61	0.27	-2.957±0.474	-0.408±0.454	-2.549±0.319	5	5	5	2.9	7.8	5	1.5	3.5	3.4	23.338	25.886	26.295	0.462	0.371	0.877	2	288.68	2561	126.18
Q99P36, Q99P34, Q9	SytVII, Syt7	0.39	0.54	0.35	-1.494±0.681	0.29±0.617	-1.784±0.744	4	5	4	1.3	2.3	2	0.5	1.2	1.4	21.497	23.281	22.991	0.418	0.948	0.815	8	58.161	523	26.873
Q3ZBA0	Tecpr1	0.34	0.45	0.38	-	-0.52±0.454	-	3	5	4	1.1	1.8	2.2	0.2	1.1	1.4	-	22.377	22.897	-	0.522	0.597	1	130.19	1166	27.439
Q9R1K2-4, Q9R1K2-	Tenm2	0.31	0.5	0.3	-0.035±0.478	0.595±0.526	-0.631±0.376	5	5	5	1.9	4.3	4.2	1.2	3.4	3.4	24.197	24.828	24.232	0.316	0.556	0.837	6	299.93	2703	75.038
F1LZ38	Tenm4	0.32	0.53	0.3	-	0.608±0.458	-	2	4	4	1	2.4	2.1	0	1.5	1.7	-	24.219	23.612	-	0.269	0.462	1	310.87	2794	53.361
F1LQ63, A0A096MJ	Tnr	0.37	0.48	0.38	-1.472±0.317	-0.428±0.218	-1.044±0.233	4	5	5	1.5	2.4	2.6	0.5	0.5	1.3	22.474	23.518	23.946	0.408	0.125	0.372	4	139.43	1266	146.03
D3ZF39	Uap1	0.38	0.49	0.4	-0.901±0.326	-0.289±0.306	-0.612±0.392	5	5	5	1.7	2.6	2.8	0.5	0.6	0.6	23.127	23.739	24.028	0.563	0.509	0.266	1	58.394	521	11.287

Scaled down (Bic) + Untd

Protein ID(s)	Gene symbol(s)	Peptide ratio Bic/Untd	Peptide ratio TTX/Untd	Peptide ratio Bic/TTX	Log2 fold change Bic-Untd (±pooled standard error)	Log2 fold change TTX-Untd (±pooled standard error)	Log2 fold change Bic-TTX (±pooled standard error)	Bic: number of biological replicates	TTX: number of biological replicates	Untd: number of biological replicates	Bic: mean unique peptides	TTX: mean unique peptides	Untd: mean unique peptides	Bic: SD unique peptides	TTX: SD unique peptides	Untd: SD unique peptides	Bic: mean Log2 LFQ	TTX: mean Log2 LFQ	Untd: mean Log2 LFQ	Bic: coefficient of variation	TTX: coefficient of variation	Untd: coefficient of variation	Number of proteins	Mol. weight [kDa]	Sequence length	Score
P06238, M0R9G2, Q	A2m, LOC10091154	0.51	0.39	0.61	0.456±0.641	-0.449±0.599	0.904±0.382	4	4	4	2.6	1.6	2.6	1.3	0.6	1.2	23.468	22.563	23.012	0.267	0.497	1.066	6	163.78	1472	17.943
Q9JK64, Q6PSM0, Q	Abcb1a, Mdr1a, Abcb	0.46	0.34	0.62	-0.44±0.359	-1.348±0.254	0.908±0.404	5	5	5	2	1.2	2.3	0.7	0.4	1.6	23.205	22.297	23.645	0.687	0.413	0.209	13	140.33	1272	36.294
P47853	Bgn	0.49	0.36	0.63	-0.25±0.662	-1.756±0.465	1.506±0.444	4	5	5	1.9	1.1	2	1.4	0.2	1.7	22.932	21.426	23.182	0.949	0.206	1.006	1	41.706	369	23.263
Q5U2R3	Frmd8	0.48	0.37	0.61	-0.298±0.253	-0.71±0.28	0.412±0.249	5	5	5	3.2	2	3.5	0.9	1.1	0.9	24.543	24.13	24.84	0.271	0.354	0.363	1	51.781	466	155.69
F1MAB8	Kif11	0.51	0.4	0.61	0.697±0.359	-1.203±0.379	1.9±0.316	5	3	4	1.9	1.2	1.8	1.2	0.3	0.7	22.916	21.016	22.219	0.329	0.144	0.413	2	118.29	1056	61.704
M0R6K0, P15800, Q	Lamb2	0.48	0.33	0.65	0.289±0.445	-2.214±0.458	2.503±0.479	5	5	5	4.6	2.4	5	1.4	1.1	1.7	25.394	22.891	25.105	0.668	0.713	0.589	3	196.53	1801	161.31
D3ZPN5	Mtpap	0.47	0	1	-0.032±0.519	-	-	4	0	3	1	0	1.1	0	-	0.2	21.998	-	22.03	0.361	-	0.691	1	37.284	336	8.2877
D4AAE6	Rab20	0.55	0.39	0.65	0.358±0.175	-	-	5	4	4	2.1	1.1	1.7	0.7	0.2	0.6	23.819	-	23.46	0.018	-	0.175	1	25.805	232	28.19
M0R8K1, D3Z8N2	Rnf187	0.5	0	1	0.739±0.618	-	-	3	0	3	1	0	1	0	-	0	22.386	-	21.647	0.794	-	0.581	2	26.295	236	2.3758
P29524	Serpinb2	0.54	0.35	0.68	0.782±0.636	-1.188±0.234	1.97±0.623	4	4	4	5.1	2.4	4.4	3.4	0.9	1.5	26.028	24.059	25.247	1.321	0.214	0.297	1	47.247	416	36.081
Q5D006, Q6IE71, F7	Usp11	0.5	0.37	0.62	-0.401±0.255	-0.835±0.242	0.434±0.189	5	5	5	1.7	1.1	1.8	0.4	0.1	0.4	22.342	21.908	22.742	0.252	0.207	0.383	4	105.24	921	12.154

Supplemental Table S8.

Mean log_2 fold change Bic/TTX	Median log_2 fold change Bic/TTX	Annotation name	Identifier	Annotation size	P value	Benj. Hoch. FDR	Annotation database
-1.01	-0.94	Ionotropic glutamate receptor complex	GO:0008328	4	1.20E-03	0.0456	Gene ontology (cellular component)
-0.682	-0.522	CAMK Ser/Thr protein kinase family	-	13	3.90E-05	0.0285	Protein families
-0.664	-0.566	Voltage-gated calcium channel complex	GO:0005891	5	1.60E-03	0.0145	GOCC slim
-0.608	-0.532	Dendritic shaft	GO:0043198	21	1.10E-05	0.0010	Gene ontology (cellular component)
-0.608	-0.532	Dendritic shaft	GO:0043198	21	1.10E-05	0.0074	Gene ontology (GO)
-0.553	-0.519	Laminin G domain	PF02210	9	6.90E-05	0.0273	Cross-reference (Pfam)
-0.513	-0.537	Calcium ion transport	GO:0006816	14	9.10E-05	0.0283	Gene ontology (GO)
-0.498	-0.513	Rho guanyl-nucleotide exchange factor activity	GO:0005089	10	1.50E-04	0.0391	Gene ontology (GO)
-0.498	-0.513	Rho guanyl-nucleotide exchange factor activity	GO:0005089	10	1.50E-04	0.0288	Gene ontology (molecular function)
-0.489	-0.378	AMPA selective glutamate receptor complex	GO:0032281	10	1.30E-03	0.0471	Gene ontology (cellular component)
-0.483	-0.355	Inflammatory response	-	8	2.80E-03	0.0353	Uniprot keywords
-0.47	-0.455	Dbl homology (DH) domain	IPR000219	12	5.80E-05	0.0195	Cross-reference (InterPro)
-0.47	-0.455	Dbl homology (DH) domain	PF00621	12	5.80E-05	0.0343	Cross-reference (Pfam)
-0.468	-0.351	Peptidyl-serine phosphorylation	GO:0018105	27	1.60E-05	0.0085	Gene ontology (GO)
-0.461	-0.338	Neurotransmitter transport	-	10	1.70E-03	0.0234	Uniprot keywords
-0.445	-0.474	Ligand-gated ion channel	-	12	1.90E-03	0.0264	Uniprot keywords
-0.44	-0.363	Guanine-nucleotide releasing factor	-	15	3.30E-05	0.0009	Uniprot keywords
-0.434	-0.376	Exocytosis	-	24	3.40E-05	0.0009	Uniprot keywords
-0.408	-0.415	Presynaptic membrane	GO:0042734	21	3.70E-04	0.0178	Gene ontology (cellular component)
-0.4	-0.369	Voltage-gated channel	-	15	2.40E-03	0.0309	Uniprot keywords
-0.394	-0.438	Postsynaptic cell membrane	-	42	6.70E-07	0.0000	Uniprot keywords
-0.391	-0.388	Protein-tyrosine phosphatase-like	IPR029021	31	1.70E-05	0.0074	Cross-reference (InterPro)
-0.373	-0.318	Neurotransmitter transport	GO:0006836	23	1.10E-03	0.0136	GOBP slim
-0.367	-0.319	Neuron migration	GO:0001764	35	1.80E-04	0.0445	Gene ontology (GO)
-0.365	-0.301	Exocytosis	GO:0006887	46	2.30E-06	0.0001	GOBP slim
-0.365	-0.365	Ion channel	-	39	2.60E-04	0.0051	Uniprot keywords
-0.362	-0.324	Exocytosis	GO:0006887	40	2.20E-05	0.0405	Gene ontology (biological process)
-0.362	-0.324	Exocytosis	GO:0006887	40	2.20E-05	0.0095	Gene ontology (GO)
-0.337	-0.26	Terminal bouton	GO:0043195	45	1.70E-05	0.0014	Gene ontology (cellular component)
-0.337	-0.26	Terminal bouton	GO:0043195	45	1.70E-05	0.0087	Gene ontology (GO)
-0.325	-0.351	Calmodulin-binding	-	24	1.50E-03	0.0220	Uniprot keywords
-0.322	-0.252	Protein kinase superfamily	-	96	3.30E-07	0.0005	Protein families
-0.302	-0.277	Perikaryon	GO:0043204	32	5.90E-04	0.0269	Gene ontology (cellular component)
-0.3	-0.335	C2 domain	PF00168	38	1.10E-05	0.0136	Cross-reference (Pfam)
-0.292	-0.25	Actin cytoskeleton organization	GO:0030036	41	1.90E-04	0.0445	Gene ontology (GO)
-0.29	-0.304	GTPase activator activity	GO:0005096	46	1.00E-04	0.0289	Gene ontology (GO)
-0.29	-0.304	GTPase activator activity	GO:0005096	46	1.00E-04	0.0247	Gene ontology (molecular function)
-0.286	-0.31	GTPase activation	-	20	3.00E-03	0.0366	Uniprot keywords
-0.239	-0.296	Site of polarized growth	GO:0030427	49	7.30E-05	0.0015	GOCC slim
0.044	0.195	Extracellular matrix	GO:0031012	28	1.50E-03	0.0145	GOCC slim
0.084	0.13	Ribosome	GO:0005840	39	5.30E-03	0.0342	GOCC slim
0.126	0.089	Translation	GO:0006412	44	2.00E-03	0.0226	GOBP slim
0.153	0.126	RNA polymerase II core promoter proximal region sequence-specific DNA b	GO:0000978	44	5.40E-05	0.0183	Gene ontology (GO)
0.153	0.126	RNA polymerase II core promoter proximal region sequence-specific DNA b	GO:0000978	44	5.40E-05	0.0157	Gene ontology (molecular function)
0.158	0.223	Proteinaceous extracellular matrix	GO:0005578	16	4.70E-03	0.0361	GOCC slim
0.178	0.113	SRP-dependent cotranslational protein targeting to membrane	REACT_345353	33	9.10E-05	0.0116	Cross-reference (Reactome)
0.211	0.151	GTP hydrolysis and joining of the 60S ribosomal subunit	REACT_338061	33	3.00E-05	0.0090	Cross-reference (Reactome)
0.215	0.151	L13a-mediated translational silencing of Ceruloplasmin expression	REACT_286215	31	5.60E-05	0.0084	Cross-reference (Reactome)
0.246	0.133	Zinc finger C2H2-type/integrase DNA-binding domain	IPR013087	47	8.60E-07	0.0007	Cross-reference (InterPro)
0.252	0.135	Zinc finger, C2H2	PF00096	28	6.50E-05	0.0308	Cross-reference (Pfam)
0.257	0.233	Formation of a pool of free 40S subunits	REACT_286803	23	3.20E-05	0.0072	Cross-reference (Reactome)
0.265	0.281	RNA polymerase II core promoter proximal region sequence-specific DNA b	GO:0001077	20	2.00E-04	0.0453	Gene ontology (GO)
0.265	0.281	RNA polymerase II core promoter proximal region sequence-specific DNA b	GO:0001077	20	2.00E-04	0.0308	Gene ontology (molecular function)
0.284	0.289	Double-stranded DNA binding	GO:0003690	30	1.10E-05	0.0069	Gene ontology (GO)
0.284	0.289	Double-stranded DNA binding	GO:0003690	30	1.10E-05	0.0064	Gene ontology (molecular function)
0.301	0.26	Nonsense Mediated Decay (NMD) independent of the Exon Junction Comple	REACT_285246	16	2.20E-04	0.0243	Cross-reference (Reactome)
0.305	0.287	Nonsense Mediated Decay (NMD) enhanced by the Exon Junction Complex	REACT_320469	25	9.10E-06	0.0081	Cross-reference (Reactome)
0.329	0.283	Citrullination	-	14	3.90E-04	0.0069	Uniprot keywords
0.365	0.282	Response to cAMP	GO:0051591	20	1.50E-04	0.0402	Gene ontology (GO)
0.369	0.209	Proteasome core complex	GO:0005839	9	8.30E-04	0.0344	Gene ontology (cellular component)
0.369	0.209	Threonine protease	-	9	8.30E-04	0.0132	Uniprot keywords
0.385	0.3	Eukaryotic Translation Termination	REACT_310475	13	3.60E-05	0.0065	Cross-reference (Reactome)
0.42	0.36	Peptide chain elongation	REACT_299036	12	1.80E-05	0.0081	Cross-reference (Reactome)
0.492	0.4	Basic-leucine zipper domain	IPR004827	17	5.10E-05	0.0184	Cross-reference (InterPro)
0.652	0.699	Basic-leucine zipper domain	PF00170	11	2.10E-05	0.0167	Cross-reference (Pfam)

Supplemental Experimental Procedures.
Bioinformatic Processing and Criteria.

Protein abundances and differential expression were evaluated using two approaches:
First, we compared protein abundances using a Label Free Quantitation (LFQ)-based approach (parameter set in Supplementary Information). We did not use “Match-between-runs” and increased the MQ-data point count to four data points. The data were processed and statistically evaluated using the Perseus software package (ver. 1.5.2.6). Protein localization for 5775 of 5940 newly-synthesized proteins were imported from the LocTree3 database (ver. 07-04-2016; Goldberg et al., 2014). Venn diagrams were generated using VennDIS (Ignatchenko *et al.*, 2015). After filtering for the presence in ≥ 2 biological replicates (4471 proteins remaining) and for a coefficient of variation < 1 (4186 proteins remaining), these proteins were annotated using UniprotKB (GOCC, GOMF, GOBP, Reactome, Pfam, InterPro, protein families) and a 1D annotation enrichment was performed (Perseus, Benjamini-Hochberg FDR = 0.05, both side test, Table S8; Cox et al., 2012) and FunRich 2.12 (Benjamini -Hochberg FDR = 0.05 for enrichment analysis; Pathan et al., 2016). Relative protein abundances analysis and statistical significance of differential regulation between control and Bicuculline, control and Tetrodotoxin or Bicuculline and Tetrodotoxin were calculated using an ANOVA test (S0=0.2, permutation-based FDR = 0.05, 250 randomizations) yielding 307 differentially regulated proteins. Proteins were mapped onto KEGG pathways (Kanehisa et al., 2016) using Incromap and bioDBnet Converter, and pathway schemes were adapted and modified (http://www.genome.jp/kegg/). Grey boxes in figures indicate detected proteins, asterisks show statistical significance in the Fisher LSD test (in Origin 2015G Sr1, OriginLab).
For visualization of the network between the 307 differentially regulated proteins, these proteins were analysed using String (v. 10.0, string-db.org; Szklarczyk et al., 2015) with experiments and databases as active interaction sources (textmining, co-expression, neighborhood, gene fusion, co-occurrence disabled; minimum required interaction score: medium, confidence 0.4, query proteins only). Proteins were plotted in custom pathway schemes (modified from KEGG) and LFQ data depicted in call-out boxes for fold changes > 0.5 (manual addition of selected candidates of interest). The 307 differentially regulated proteins were analyzed for correlation with diseases using GeneAnalytics (“MalaCards”, Lifemap Sciences), and association with diseases listed in brief in Figure 8 and in detail in Table S7.

Second, we set out to analyze proteins identified exclusively in one condition. As no high-abundance proteins with high peptide counts and intensity values were detected exclusively, we analyzed proteins with low peptide and intensity counts that displayed a trend in abundance/detection for a specific condition. Here, we first manually evaluated candidates detected exclusively in 3/5 biological replicates in one condition and not detected in any other condition and examined their normalized peptide intensities to exclude detection-limit-based false positive hits. Only proteins displaying similar peptide counts and intensity profiles (based on normalized peptide intensity values) were retained for further analysis. All candidates including peptide counts, intensity values and fold-changes are listed in Table S2, page 2. We also made use of unique peptide ratios and normalized peptide intensity values for manually selected low-abundance proteins. We again focused on proteins with < 5 peptides in a group without robust LFQ data. We approximated abundance by comparing unique peptide counts and extracted proteins with D > 0.6 or D < 0.4 (D= x / x+y, with x = peptides in condition A and y peptides in condition B) and only retained proteins displaying similar trends in normalized intensity ratios. Due to the low peptide counts and intensity values available, the analyses described in the first part do not quantitative values, but rather suggest a trend in abundance.

Supplemental references

Cox, J., and Mann, M. (2012). 1D and 2D annotation enrichment: a statistical method integrating quantitative proteomics with complementary high-throughput data. BMC Bioinformatics 13, S12.

Goldberg, T., et al. (2014). LocTree3 prediction of localization. Nucleic Acids Res 42, W350-W355.

Ignatchenko, V., Ignatchenko, A., Sinha, A., Boutros, P.C., and Kislinger, T. (2015). VennDIS: a JavaFX-based Venn and Euler diagram software to generate publication quality figures. Proteomics 15, 1239-1244.

Kanehisa, M., Sato, Y., Kawashima, M., Furumichi, M., and Tanabe, M. (2016). KEGG as a reference resource for gene and protein annotation. Nucleic Acids Res 44, D457-D462.

Pathan, M., Keerthikumar, S., Ang, C.S., Gangoda, L., Quek, C.M.J., Williamson, N.J., Mouradov, D., Sieber, O.M., Simpson, R.J., Salim, A., Bacic, A., Hill, A.F., Stroud, D.A., Ryan, M.T., Agbinya, J.A., Mariadasson, J.M., Burgess, A.W. and Mathivanan, S. (2015). FunRich: a standalone tool for functional enrichment analysis. Proteomics 15, 2597-2601.

Szklarczyk, D., Franceschini, A., Wyder, S., Forslund, K., Heller, D., Huerta-Cepas, J., Simonovic, M., Roth, A., Santos, A., Tsafou, K.P., Kuhn, M., Bork, P., Jensen, L.J., and von Mering, C. (2015). STRING v10: protein-protein interaction networks, integrated over the tree of life. Nucleic Acids Res 43, D447-D452.
