# Supplementary material for: Nascent Proteome Remodeling following Homeostatic Scaling at Hippocampal Synapses
Source: Neuron. 2016 Oct 19;92(2):358–71. doi: 10.1016/j.neuron.2016.09.058 (PMC5078608; doi:10.1016/j.neuron.2016.09.058)
Supplement: Document S2. Article plus Supplemental Information [file mmc10.pdf]

NeuroResource

Neuron

Nascent Proteome Remodeling following Homeostatic Scaling at Hippocampal Synapses

Highlights

- Newly synthesized proteins were metabolically labeled during homeostatic scaling
- Approximately 6,000 newly synthesized proteins were detected using mass spectrometry
- Over 300 proteins were differentially regulated by synaptic up- and down-scaling
- Important synaptic proteins and disease-related proteins were regulated

Authors

Christoph T. Schanzenbächer,
Sivakumar Sambandan,
Julian D. Langer, Erin M. Schuman

Correspondence

julian.langer@brain.mpg.de (J.D.L.),
erin.schuman@brain.mpg.de (E.M.S.)

In Brief

Schanzenbächer et al. discover the newly synthesized proteome in response to the global activity manipulations that bring about homeostatic synaptic scaling. The synthesis of over 300 proteins was significantly differentially regulated by the induction of scaling, including neurotransmitter receptors, scaffolding, and signaling molecules.

Accession Numbers

PXD004889

CrossMark
Schanzenbächer et al., 2016, Neuron *92*, 358–371
October 19, 2016 © 2016 The Author(s). Published by Elsevier Inc.
http://dx.doi.org/10.1016/j.neuron.2016.09.058

CellPress

Cell Press

Neuron
NeuroResource

Nascent Proteome Remodeling following Homeostatic Scaling at Hippocampal Synapses

Christoph T. Schanzenbächer,[1,2] Sivakumar Sambandan,[1] Julian D. Langer,[1,2,*] and Erin M. Schuman[1,2,3,*]
[1]Max Planck Institute for Brain Research, Max von Laue Strasse 4, 60438 Frankfurt am Main, Germany
[2]Max Planck Institute for Biophysics, Max von Laue Strasse 3, 60438 Frankfurt am Main, Germany
[3]Lead Contact
*Correspondence: julian.langer@brain.mpg.de (J.D.L.), erin.schuman@brain.mpg.de (E.M.S.)
http://dx.doi.org/10.1016/j.neuron.2016.09.058

SUMMARY

Homeostatic scaling adjusts the strength of synaptic connections up or down in response to large changes in input. To identify the landscape of proteomic changes that contribute to opposing forms of homeostatic plasticity, we examined the plasticity-induced changes in the newly synthesized proteome. Cultured rat hippocampal neurons underwent homeostatic up-scaling or down-scaling. We used BONCAT (bio-orthogonal non-canonical amino acid tagging) to metabolically label, capture, and identify newly synthesized proteins, detecting and analyzing 5,940 newly synthesized proteins using mass spectrometry and label-free quantitation. Neither up- nor down-scaling produced changes in the number of different proteins translated. Rather, up- and down-scaling elicited opposing translational regulation of several molecular pathways, producing targeted adjustments in the proteome. We discovered ~300 differentially regulated proteins involved in neurite outgrowth, axon guidance, filopodia assembly, excitatory synapses, and glutamate receptor complexes. We also identified differentially regulated proteins that are associated with multiple diseases, including schizophrenia, epilepsy, and Parkinson's disease.

INTRODUCTION

Neuronal networks are subject to fluctuations in both the magnitude and frequency of inputs, requiring plasticity mechanisms to stabilize network activity. Homeostatic synaptic scaling adjusts the strength of neuronal connections up or down in response to changes in input (Davis, 2013; Turrigiano, 2012). At the *Drosophila* neuromuscular junction, genetic manipulations that alter postsynaptic receptor function result in compensatory changes in presynaptic neurotransmitter release (see Davis, 2013 for review). At mammalian synapses, blocking action potential-mediated activity either pharmacologically (O'Brien et al., 1998; Turrigiano et al., 1998) by occluding sensory input (Desai et al., 2002) or by suppressing excitability (Burrone et al., 2002) can lead to a global up-scaling of synaptic responses. Conversely, a global stimulation of activity, effected by blocking $GABA_A$ receptor-mediated inhibition, leads to a significant down-scaling of synaptic responses (O'Brien et al., 1998; Turrigiano et al., 1998).

What is the nature of the molecular changes that bring about the synaptic scaling at mammalian synapses? Several groups have postulated that fluctuations in intracellular Ca^{2+} levels initially "sense" changes in activity (Ibata et al., 2008; Thiagarajan et al., 2005) that may be read out by changes in the activation of CaMKIV resulting in changes in gene expression (Ibata et al., 2008). In addition, the expression of scaling up is mediated by the accumulation of GluR2-containing receptors (Gainey et al., 2009, 2015), likely driven by alterations with the glutamate receptor-interacting protein-1, GRIP-1 (Gainey et al., 2015), and protein-interacting-with C Kinase 1, PICK1 (Anggono et al., 2011). Other molecules implicated in various aspects of synaptic scaling include the neurotrophin brain-derived neurotrophic factor (BDNF) (Rutherford et al., 1998), the immediate early gene Arc (Shepherd et al., 2006), the cytokine TNFα (Steinmetz and Turrigiano, 2010; Stellwagen and Malenka, 2006), the immune molecule MHC1 (Goddard et al., 2007), β3 integrins (Cingolani et al., 2008), and the scaffold proteins PSD-95 and PSD-93 (Sun and Turrigiano, 2011). All of the above molecules were discovered using a candidate-based approach, making good "guesses" about the players that might be important, leaving open the possibility that many important molecules have not yet been discovered.

The long time course (~24 hr) required for most forms of synaptic homeostasis to be established (O'Brien et al., 1998; Turrigiano et al., 1998) suggests a requirement for changes in gene expression, either by regulated transcription or translation, or both. Indeed, upscaling elicited by a 24 hr TTX treatment is blocked by a transcription inhibitor (Ibata et al., 2008). In a form of homeostatic depression elicited by ChR2-stimulation of individual neurons, a requirement for both transcription and translation has been reported (Goold and Nicoll, 2010). Whether the classic homeostatic plasticity, elicited pharmacologically, requires changes in new protein translation has, surprisingly, not been addressed.

What is known about the transcriptomic and proteomic alterations that underlie homeostatic plasticity? A recent study

 © 2016 The Author(s). Published by Elsevier Inc.
This is an open access article under the CC BY license (http://creativecommons.org/licenses/by/4.0/).

studied a few selected transcripts with qPCR and showed that homeostatic upscaling resulted in a significant increase in the *Gria1*, *Tet1*, and *Gadd 45b* transcripts (Meadows et al., 2015). While alterations in transcripts can lead to changes in protein abundance, several studies have shown that in complex systems the correlation between the transcriptome and the proteome can be low (Gygi et al., 1999; Komatsu and Hossain, 2013; Jovanovic et al., 2015). As proteins are the primary and most common effectors in all cellular processes, it is important to determine the proteome directly to understand how plasticity fundamentally alters the neuronal and synaptic landscape.

Recent advances in proteomic technology have led to unprecedented coverage and depth in the identification of proteins in cells and tissues. A recent study, for example, identified over 8,000 proteins in each of several different brain areas, including the hippocampus (Sharma et al., 2015). In addition, over 2,000 proteins reliably associated with the postsynaptic density have also been identified (Distler et al., 2014; Peng et al., 2004; Pielot et al., 2012; Cajigas et al., 2012). Quantitative proteomic analyses that focus on the entire (unlabeled) proteome, however, do not allow one to identify proteins synthesized in response to plasticity. For example, if one observes an increase in protein levels, it is impossible to determine whether the increase is due to enhanced protein synthesis or decreased protein degradation. In order to isolate the analysis of the newly synthesized proteome associated with plasticity, metabolic labeling is required. Bio-orthogonal non-canonical amino acid tagging (BONCAT) enables pulsed metabolic labeling of newly synthesized proteins using non-canonical amino acids like azidohomoalanine (AHA) together with click-chemistry followed by mass spectrometry (Dieterich et al., 2006, 2007). Using this method, alterations in newly synthesized proteins can be directly determined. This approach has been applied to identify newly synthesized proteins in many systems (Landgraf et al., 2015), including in the hippocampus following treatment with dopamine (Hodas et al., 2012) or BDNF (Bowling et al., 2016).

The opposite phenotypes elicited during synaptic up-scaling and down-scaling represent a unique opportunity to examine the underlying proteomic plasticity and identify pathways that are bi-directionally or coordinately regulated. Here we detect an unprecedented number of newly synthesized proteins and provide a comprehensive map of the newly synthesized proteome following both up- and down-scaling.

RESULTS

To ascertain, first, whether pharmacologically elicited synaptic scaling requires protein synthesis, we conducted experiments in which protein synthesis was inhibited during the scaling manipulation (Figure S1). In order to elicit up-scaling, cultured hippocampal neurons were treated with the Na^+ channel antagonist tetrodotoxin (1 μM) for 24 hr to elicit an increase in miniature excitatory postsynaptic current (mEPSC) amplitude (Figure S1A). The co-application of the protein synthesis inhibitor anisomycin (40 μM) prevented the mEPSC amplitude increase (Figure S1B). In order to elicit down-scaling, cultured hippocampal neurons (DIV 21) were treated with the $GABA_A$ receptor antagonist bicuculline (40 μM) for 24 hr to elicit a decrease in mEPSC amplitude (Figure S1A). The co-application of the protein synthesis inhibitor anisomycin (40 μM) prevented the mEPSC amplitude decrease, indicating a requirement for new protein synthesis (Figure S1C). Taken together, these data demonstrate that protein synthesis is required for both up- and down-scaling elicited pharmacologically in cultured neurons.

In order to identify the specific proteins synthesized following global manipulations of neuronal activity, we used BONCAT (Figures 1 and 2; Dieterich et al., 2006) together with the paradigm of homeostatic scaling. For the duration of the scaling induction (24 hr of either bicuculline or TTX, as above) cultured hippocampal neurons (DIV 21) were treated with the non-canonical amino acid azidohomoalanine (AHA; 4 mM) to label the newly synthesized proteins associated with homeostatic scaling. A control group was exposed to AHA for 24 hr in the absence of either bicuculline or TTX. An additional negative control group was treated with methionine, the amino acid from which AHA is derived (Met; 4 mM) (Figure 2A). Following scaling induction, neuronal lysates were prepared and BONCAT (see Experimental Procedures) was performed to capture, purify, and identify the newly synthesized proteins (Figure 2A). For each set of experiments, we conducted five independent biological replicates that were each injected in four technical replicate LC-MS/MS runs (see Experimental Procedures; Figure 1 and S2). To identify proteins, we required a minimum of one tryptic peptide per protein (see Experimental Procedures; Table S9). With a few exceptions (see Experimental Procedures), any protein detected in a methionine control experiment was subtracted from our experimental dataset, and only proteins present in more than two biological replicates were retained for subsequent analysis.

In the above experiments, we obtained an unprecedented coverage and depth of the newly synthesized proteome. Our master dataset, comprising the union of the untreated, scaled-up, and scaled-down groups, includes 5,940 unique protein groups representing all key cellular processes, including metabolic enzymes, cell signaling, and structural proteins (Figures 1 and 2B; Table S1). We were able to detect most of the important neuronal membrane proteins including ion channels and a majority of the subunits of the excitatory and inhibitory neurotransmitter receptors, including AMPA and NMDA receptor subunits as well as $GABA_A$ and $GABA_B$ receptors (Figures 5 and S5; Tables S1 and S3). In addition, we identified many membrane proteins associated with presynaptic membranes and vesicles, including both vesicular and plasma membrane transporters (Figure 6; Tables S1 and S3). These data are described in much greater detail below.

In order to assess the depth and breadth of our newly synthesized proteome, we compared the newly synthesized proteome of control (not subjected to scaling) AHA-treated neurons to a global hippocampal proteome dataset (Sharma et al., 2015) (see Experimental Procedures; Figure 1). We found that there was substantial overlap between the pulse (24 hr)-labeled nascent hippocampal proteome and the global hippocampal proteome (Figure 2C). The AHA-labeled proteome also captured many proteins not identified in the global proteome, likely owing to the specific enrichment and reduced sample complexity of the AHA-proteome and the resulting expanded dynamic range. We considered the possibility that some of the AHA-exclusive

protein filtering
transformation
manipulation
analysis

Treatment	Enzyme	Variable modifications (fixed)	Peptide / protein characteristics	Biological replicates	Technical replicates	LFQ group
negative control	trypsin/P, max. 2 missed cleavages	oxidation [M], deamidation [NQ], acetylation [K], carbamylation [N-term], (carboxyamidomethylation [C])	≥6 AAs, PSM FDR 0.01, peak length ≥4 data points, protein FDR 0.01, min. unique peptides ≥1, quantification: unique peptides	5	4	none
vehicle						0
scaled-down						1
scaled-up						2

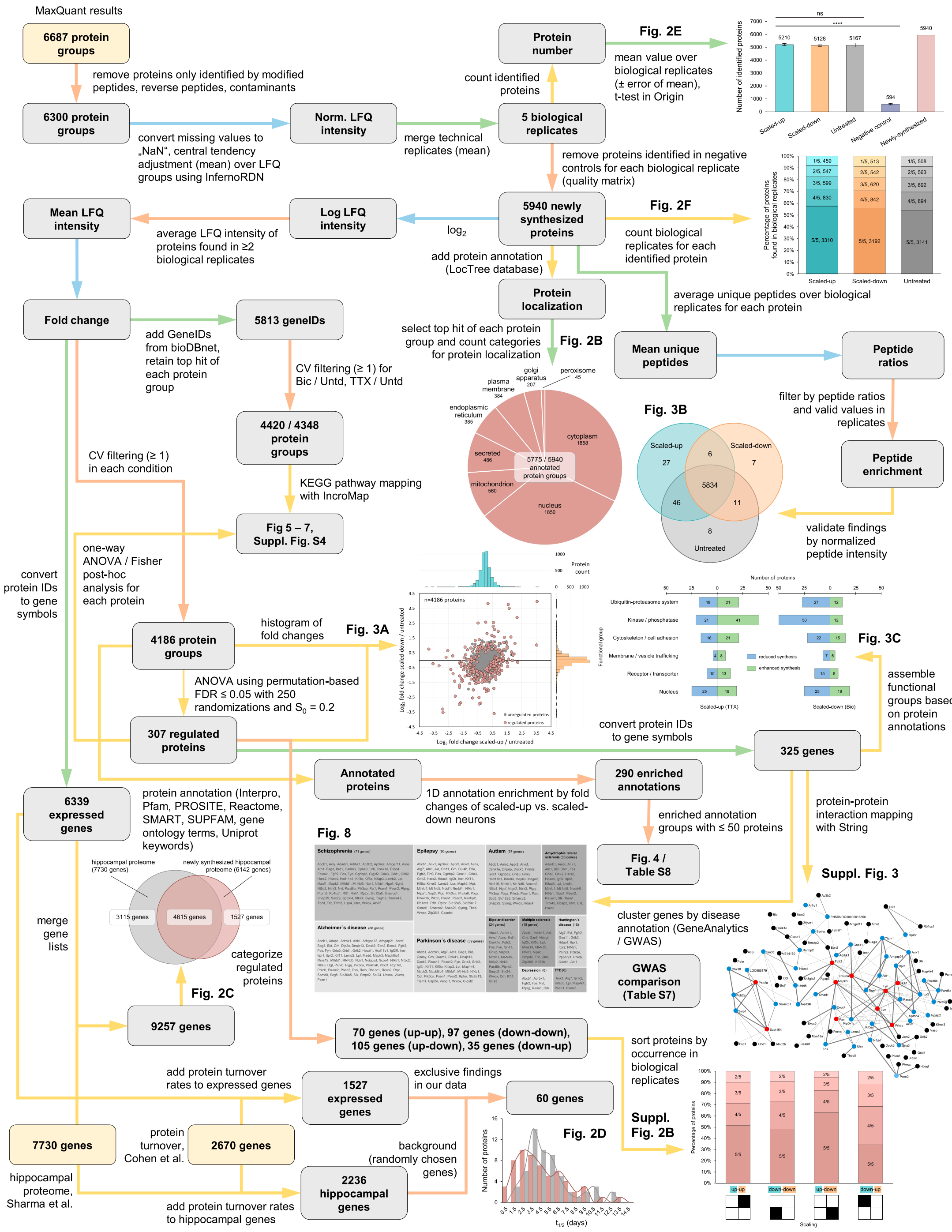

Figure 1. Overall Workflow Including Sample Structure, Preparation, Analyses and Data Processing, and Evaluation

Flow chart explaining the datasets used, how the data were filtered, and how we calculated the numbers of proteins in each figure as well as the conversion to genes, when appropriate.

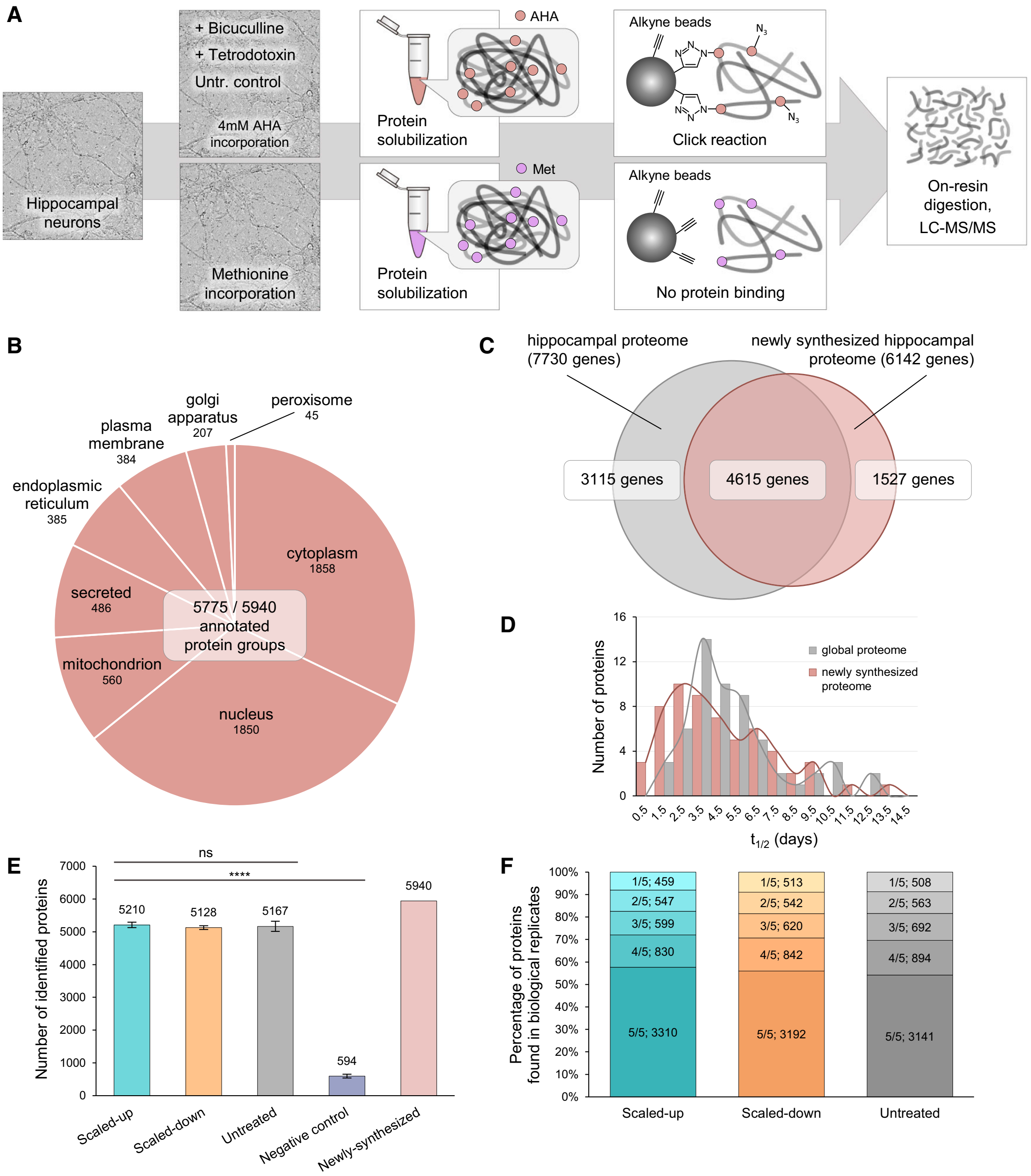

Figure 2. Workflow and Characterization of the Newly Synthesized Proteome

(A) Workflow of experiments. Cultured hippocampal neurons (DIV 21) were treated with AHA (4 mM) and either vehicle, TTX (1 μM), or bicucculine (20 μM) for 24 hr. Control cultured neurons were exposed to methionine (4 mM) for 24 hr. Neurons were then harvested, and proteins were solubilized and clicked to alkyne-bearing beads. An on-resin digestion was performed, and peptides were then identified via liquid chromatography-mass spectrometry (LC-MS/MS). Proteins were identified and quantified using MaxQuant and other tools (see Experimental Procedures).

(B) Representation of protein groups in different cellular compartments.

(C) Venn diagram of the global hippocampal proteome (identified in Sharma et al., 2015) compared to the newly synthesized proteome (identified by 24 hr of AHA labeling).

(D) Plot of protein half-lives for the global versus newly synthesized proteomes. Available data from Cohen et al. (2013) for 60 AHA-labeled and global proteome proteins are shown. There is a trend for shorter half-lives of the AHA-identified proteins.

(E) Graph indicating the number of identified proteins across biological replicates for each group. Homeostatic scaling does not change the size of the nascent proteome. A significantly smaller number of proteins were identified in the negative control (methionine-treated) group ($p < 0.0001$), but none of the other groups differed significantly from one another. Error bars represent ± SEM.

(F) Reproducibility of protein identifications across biological replicates within each group.

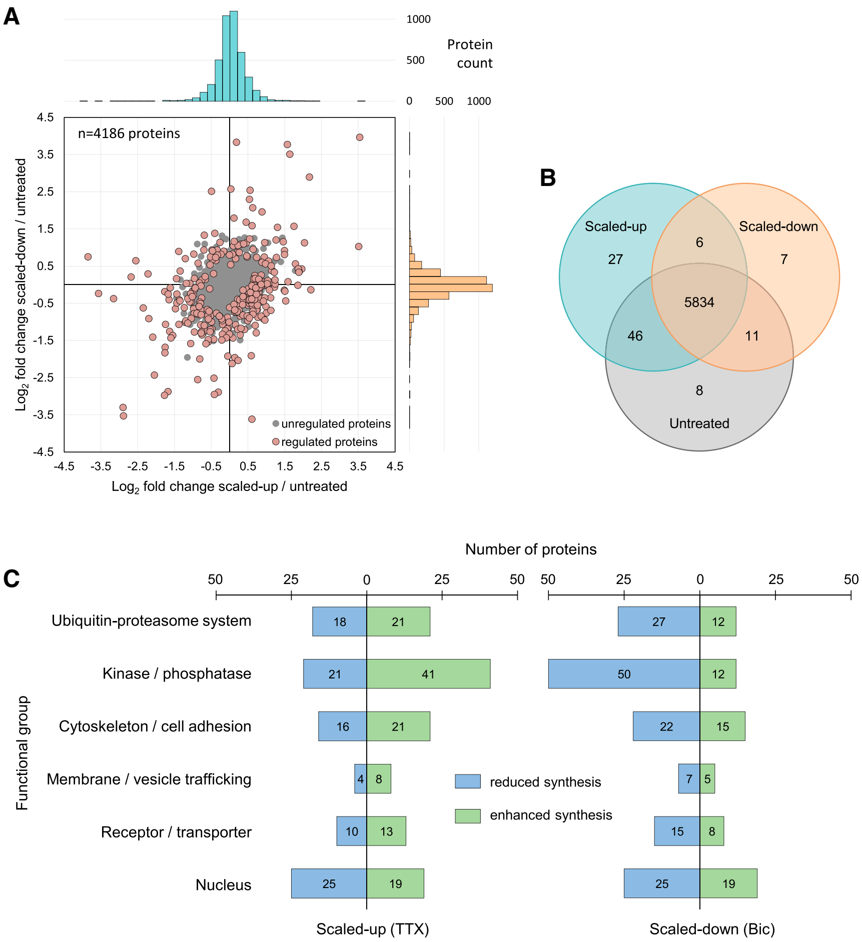

Figure 3. The Synthesis of ~300 Proteins Is Differentially Regulated by Homeostatic Scaling

(A) Scatterplot indicating every analyzed protein (n = 4,186). Proteins that were significantly regulated by homeostatic plasticity are indicated as mauve dots; all other proteins are gray. Histograms on the horizontal (teal) and vertical (orange) axes indicate the number of proteins that exhibited a given fold-change on the x (teal) and y (orange) axes.

(B) Venn diagram depicting the total number and low-abundant, newly synthesized proteins identified in each of the following groups: scaled-up (TTX-treated), scaled-down (bicuculline-treated), or untreated (control). Proteins were manually extracted as outlined in Experimental Procedures.

(C) Functional groups of significantly regulated proteins observed following up- or down-scaling.

proteins represent short-lived proteins that are less likely to be detected in the large global proteome sample. To examine this possibility, we compared the reported protein half-lives for the AHA-exclusive proteins to a comparable number of proteins from the global hippocampal proteome. Using data from Cohen et al. (2013), we extracted all of the half-life data for AHA-exclusive proteins analyzed in their dataset (n = 60) and compared the distribution of half-lives to the same number of proteins subsampled from the global hippocampal proteome. We observed a shift toward a shorter half-life in the distribution for AHA-labeled proteins (Figure 2D), suggesting that one feature of the AHA-exclusive proteins may be a faster turnover, making the proteins more difficult to capture and detect in global proteomic studies.

We next analyzed the newly synthesized proteome in control (untreated), scaled-up, and scaled-down samples. First we examined the total nascent proteome size and discovered that homeostatic scaling does not significantly alter the number of unique newly synthesized proteins in the total proteome. The total nascent proteome size in each group (control, scaled-up, and scaled-down) was between 5,100 and 5,200 proteins (Figures 1 and 2E; Table S1). In the negative control methionine-treated samples, there were ~594 proteins detected. Notably, in each AHA-treated group, over ~80% of the proteins were identified in 3/5 biological replicates and over ~90% of the proteins were identified in 2/5 biological replicates (Figure 2F). As there was no significant difference in the newly synthesized proteome size between groups, these data indicate that homeostatic scaling is associated with a balanced stimulation and inhibition of synthesis of protein groups.

We hypothesized that the dominant class of proteomic change during scaling would comprise changes in the abundance of proteins that perform essential cellular and synaptic functions. To analyze this, we compared protein abundances in the up-scaled or down-scaled nascent proteomes to control nascent proteomes using a label-free quantitation (LFQ)-based analysis (see Experimental Procedures; Figure 1). Altogether, we discovered over 300 proteins with significantly regulated expression levels following homeostatic plasticity (Figures 3A and S4; Table S2). This group comprises proteins that are co-regulated or singly regulated by either up- or down-scaling. Figure 3A depicts all proteins analyzed, and the four quadrants in the graph highlight four basic categories of significantly regulated newly synthesized proteins. Two quadrants depict proteins that exhibit coordinate regulation: both up- and down-scaling result in either an enhancement (upper right quadrant) or a reduction (lower left quadrant) in the synthesis of particular proteins. When compared to control samples, there were 70 proteins that exhibited enhanced protein synthesis associated with both up-scaling and down-scaling (Figure 3A, upper right quadrant). Some of the protein families that were overrepresented include the following: transcriptional activation, mRNA transport and splicing, and tyrosine phosphatases and kinases (Table S3). On the other hand, when compared to control samples, there were 97 proteins that exhibited reduced protein synthesis during both up-scaling and down-scaling (Figure 3A, lower left quadrant). Functional annotation analysis reveals a significant

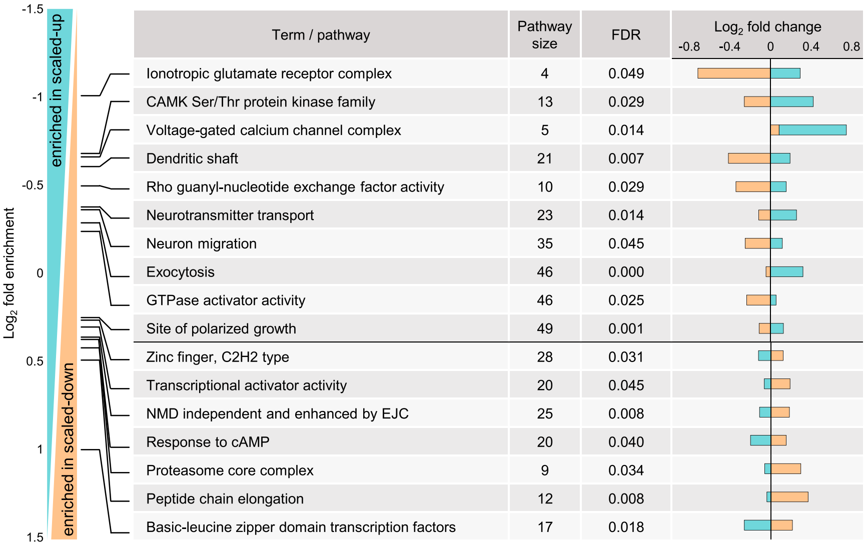

Figure 4. Differential Enrichment of Up- and Down-scaled Nascent Proteins

Selected significantly regulated protein groups or pathways that exhibit opposite or differential regulation by up- and down-scaling (teal and orange bars, respectively). FDR = false discovery rate.

enrichment of protein families mediating inositol-1,4,5-triphosphate-sensitive calcium release, sphingomyelin degradation, and cell-cell adhesion essential for dendritic spine morphogenesis as well as other cellular functions (Table S3). Taken together, these two groups of proteins exhibit consistent enhanced or reduced synthesis, regardless of the sign of the plasticity, and thus might be considered as "general" scaling proteins that respond to global changes in neuronal activity.

The remaining categories are represented by proteins significantly altered in a bi-directional manner: for these proteins, enhanced synthesis is associated with one form of plasticity (either up- or down-scaling) while reduced synthesis is associated with the other form (Figures 3A and 4; Table S2). When compared to control samples, there were 105 proteins that exhibited enhanced protein synthesis associated with up-scaling and reduced protein synthesis during down-scaling (Figure 3A, lower right quadrant). GO analysis of this group indicates a significant enrichment for proteins largely involved in synaptic function, including, for example, the family of Ca^{2+}-calmodulin-dependent protein kinases (CamK) and proteins involved in neurotransmitter transport and exocytosis (Figure 4; Table S3). Indeed, the most differentially regulated group was the ionotropic glutamate receptor complex, the target of many mechanistic studies of homeostasis (Table S5) (Turrigiano, 2012). When compared to control samples, there were 35 proteins that exhibited enhanced protein synthesis during down-scaling and reduced protein synthesis during up-scaling (Figure 3A, upper left quadrant; Figure 4). Functional annotation analysis of this group indicates a significant enrichment of calcium-dependent protein serine/threonine phosphatases, splicing factors, transcription factors, calcineurin regulators, and synaptotagmins. Additional enriched proteins in this group are implicated in mRNA deadenylation, ubiquitin activation, cell growth, and other categories (Table S3). The above proteins should thus be considered as "specific" scaling proteins that respond with a specific signature to either homeostatic up- or down-scaling.

We also considered the entire population (~307) of regulated nascent proteins as a single group and examined, using a String analysis, the networks of regulated proteins and the interactions between them (Figure S3). We identified one large network of regulated proteins containing 95 network members with the number of interaction partners ranging from 1 to greater than 7. Protein hubs in this network were represented by several protein kinases including the Fyn kinase, Lyn kinase, Map kinase 3, and Protein kinase C beta. In addition, other proteins such as the FGF receptor were linked to several other significantly regulated proteins. The remaining networks comprising significantly regulated proteins were small, ranging from 2 to 6 proteins, and the remaining regulated proteins were not linked to networks.

Finally, we considered the possibility that elements of the homeostatic proteomic response could be newly synthesized proteins that were uniquely and exclusively associated with either the control or scaled groups. We found no highly abundant (> 10 peptides) proteins exclusively in scaled-up or scaled-down neurons. As regulatory proteins may be low abundant or only yield a low number of detectable peptides, we manually inspected low-abundance proteins in our dataset based on reproducibility, spectral counting, and normalized peptide intensity ratios (for a detailed description, see Experimental Procedures). Acknowledging that this approach yields only trends rather than precise quantitative values, we identified 27 and 7 proteins with higher abundance in scaled-up neurons or scaled-down neurons, respectively (Figures 1 and 3B; Tables S1 and S2). Using these "relaxed" criteria, newly synthesized proteins enriched in scaled-up neurons were implicated in glutamatergic synapses and excitability (Adcy2, Grin2a), calcium transport (Atp2b3), nerve growth factor signaling (Lmtk2), and other cellular functions (Table S2). The newly synthesized proteins enriched in scaled-down neurons included proteins involved in synapse formation (Arhgap) and modulation of dendritic spines (Dgkz) (Table S2). An additional six proteins were found to be enriched in both scaled groups (Table S2), including proteins involved in modulation of dendritic spine shape (Synpo), regulation of the number of excitatory synapses (C1ql3), and axonal branching (Fgf2).

We next determined the changes that occurred in different cell types, compartments, and signaling pathways. Within the postsynaptic compartment (Figure 5; Table S4), we found several examples where the opposite phenotypic endpoints of homeostatic scaling are associated with opposing effects on glutamate receptors. For example, some subunits of AMPA-type (Gria3, Grid1) and a kainate-type (Grik2) receptor showed a pattern of

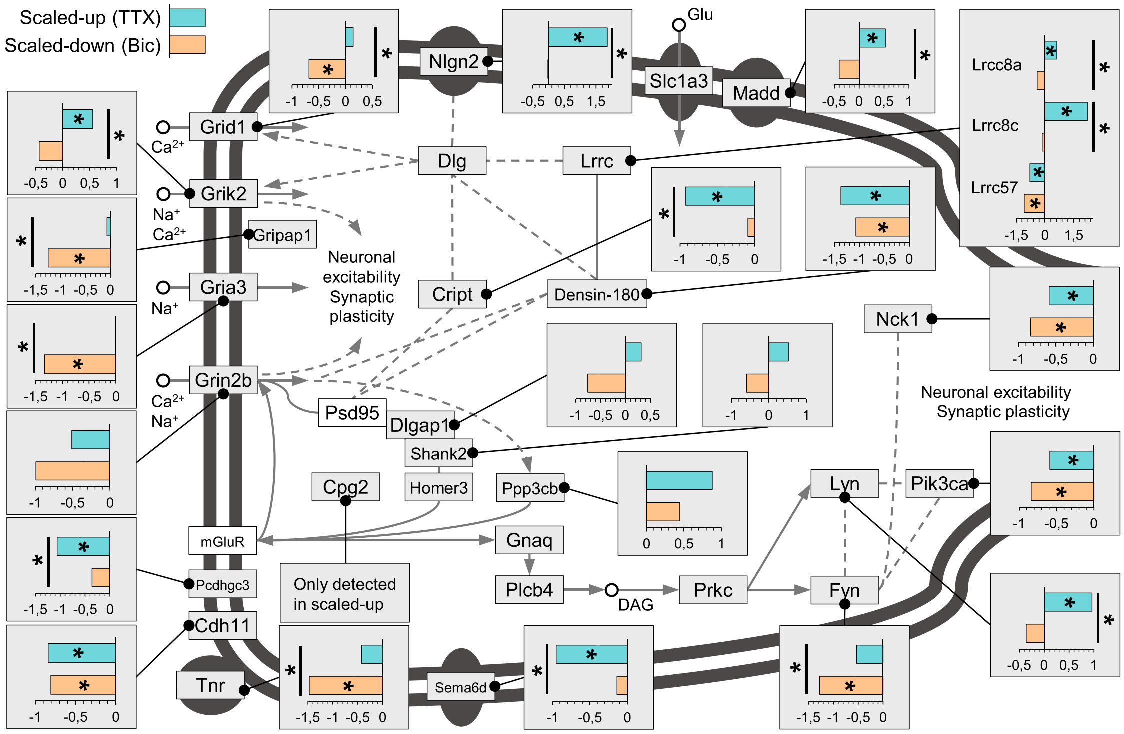

Figure 5. Proteins Associated with Postsynaptic Function at Excitatory Synapses that Are Regulated by Homeostatic Scaling

The glutamatergic synapse. Proteins in white or gray boxes were detected in this study. Call-out boxes display intensity values (see Experimental Procedures). The bars, teal and ochre, represent the regulation of the protein in up- and down-scaling, respectively. Asterisks within the colored bar indicate that the protein exhibited significant (ANOVA FDR < 0.05 and Fisher LSD post hoc p < 0.05) regulation relative to control; asterisks associated with the black lines indicate the protein exhibited significant (ANOVA FDR < 0.05 and Fisher LSD post hoc p < 0.05) regulation between up- and down-scaling.

upregulation during scaling-up accompanied in some cases (Grid1, Grik2) by downregulation during scaling-down, as expected if the receptors are modulated to increase or decrease sensitivity to neurotransmitter (Figure 5; Table S4). (Unfortunately, we did not obtain a sufficient number of unique peptides for Gria1 and 2 to conduct an analysis.) The NMDA receptor subunit Grin2b exhibited a trend for downregulated synthesis in both up- and down-scaling, although to a much greater extent during down-scaling (Figure 5). Other postsynaptic components that underwent regulation include Cript (Niethammer et al., 1998) and Lrcc7 (Densin-180). Map kinase 3 (Mapk3), a kinase implicated in long-term potentiation (LTP; Mazzucchelli et al., 2002) and implicated in autism spectrum disorders (Kumar et al., 2008), was significantly differentially regulated: enhanced in scaled-up conditions and reduced in scaled-down conditions (Tables S2 and S4). Several adhesion molecules, Tnr, Cadh11, Pcdhgc3, and Sema6d, were also significantly regulated with a clear trend for downregulation in both forms of scaling (Figure 5; Tables S2 and S4), reminiscent of earlier studies showing that downregulation of adhesion is required for structural plasticity at synapses (Mayford et al., 1992; Schuster et al., 1996). Nlgn2, in contrast, was significantly upregulated by homeostatic up-scaling (Figure 5; Tables S2 and S4).

Within the functional area of vesicular trafficking and neurotransmitter release, we observed many significantly regulated proteins (Figure 6; Table S4). Some vesicle-associated proteins like members of the syntaxin family (Syt6 and Esyt1) exhibited enhanced synthesis following down-scaling, whereas Snap29, a protein involved in multiple membrane trafficking steps, and Brsk1, a protein implicated in neurotransmitter release, were significantly differentially regulated by homeostatic scaling and reduced following down-scaling (Figures 4 and 6). Some proteins in the Ap-3 family (Ap3b2 and Ap3m2), a heterotetrameric vesicle-coat protein complex that is important for protein sorting and clathrin recruitment, exhibited significantly different expression with up- and down-scaling and were significantly upregulated with up-scaling (Figures 4 and 6; Tables S2 and S4). Related to the actin cytoskeleton, several proteins (Evl, Sptbn4, and Vasp) showed extremely robust and significant regulation consistently with down-scaling, but also with up-scaling (Figure 6; Tables S2 and S4).

As homeostatic plasticity is known to involve regulation of inhibitory transmission (Hartman et al., 2006; Kilman et al., 2002; Maffei et al., 2004), we next examined the protein components of GABAergic synaptic transmission that exhibit altered protein synthesis (Figure S5). The family of $GABA_A$ receptors was significantly regulated and exhibited, in general, opposite changes for up- and down-scaling (Figure S5). The GABA receptor scaffolding molecule Gephyrin showed a similar trend (Figure S5). Neuroligin 2 (Nlgn2), a protein implicated in the clustering of GABA receptors, and Kirrel3, a protein implicated in GABAergic synapse formation, both exhibited significant

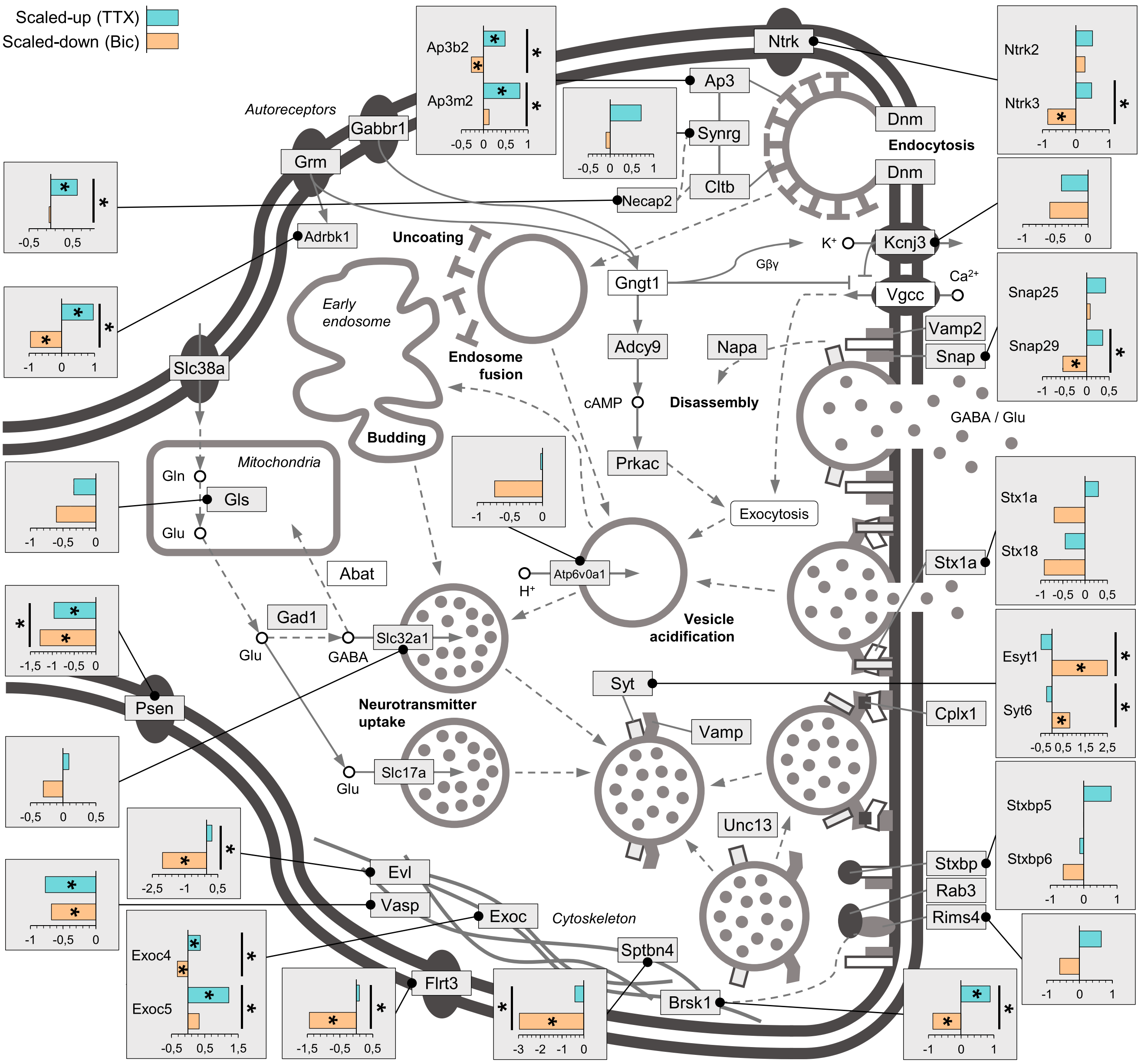

Figure 6. Proteins Associated with Presynaptic Function which Are Regulated by Homeostatic Scaling

The presynaptic terminal. Proteins in white or gray boxes were detected in our samples; call-out boxes display intensity values (see Experimental Procedures). The bars, teal and ochre, represent the regulation of the protein in up- and down-scaling, respectively. Asterisks within the colored bar indicate that the protein exhibited significant (ANOVA FDR < 0.05 and Fisher LSD post hoc p < 0.05) regulation relative to control; asterisks associated with the black lines indicate the protein exhibited significant (ANOVA FDR < 0.05 and Fisher LSD post hoc p < 0.05) regulation between up- and down-scaling.

upregulation following up-scaling (Figure S5). Synthesis of the vesicular GABA transporter, Slc32a1, was reduced in down-scaled neurons and slightly enhanced in up-scaled neurons (Figure S5). Interestingly, Kif5a, a microtubule-dependent motor essential for the transport of $GABA_A$ receptors (Nakajima et al., 2012) was significantly differentially regulated and upregulated in scaled-up neurons (Figure S5).

Since AHA-labeled newly synthesized proteins can originate from either the glial or neuronal cells present in our hippocampal cultures, we used bioinformatics (GeneAnalytics; see Experimental Procedures) to separately analyze the proteins associated with glial function. For the 5,940 proteins in the global AHA-labeled proteome, there are 6,339 associated genes; of these, 929 genes (14.7%) are associated with glial function (Table S6). For the 307 significantly regulated proteins in the AHA-proteome, there are 325 associated genes; of these, 41 genes (12.6%) are associated with glial function (Table S6). It is important to note that the GeneAnalytics tool we used identifies

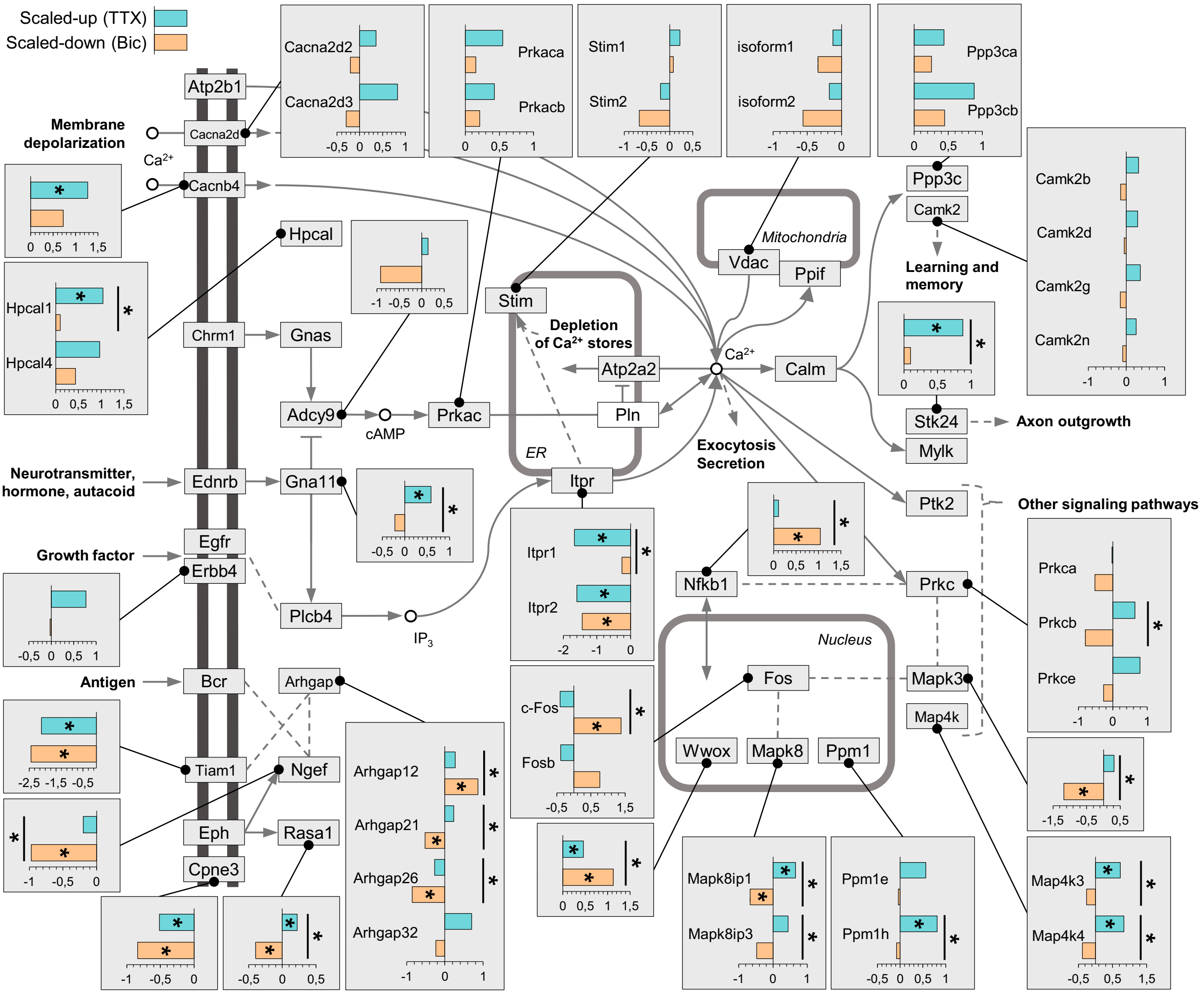

Figure 7. Ca^{2+} Signaling Pathway Proteins that Are Regulated by Homeostatic Scaling

Intracellular signaling pathways. Proteins in white or gray boxes were detected in our samples; call-out boxes display intensity values (see Experimental Procedures). The bars, teal and ochre, represent the regulation of the protein in up- and down-scaling, respectively. Asterisks within the colored bar indicate that the protein exhibited significant (ANOVA FDR < 0.05 and Fisher LSD post hoc $p < 0.05$) regulation relative to control; asterisks associated with the black lines indicate the protein exhibited significant (ANOVA FDR < 0.05 and Fisher LSD post hoc $p < 0.05$) regulation between up- and down-scaling.

glial-associated genes, but does not identify glial-exclusive genes. As such, some of the regulated genes are shared with neurons, and the cell type where the protein is significantly altered—glia, neurons, or both—is not certain. Cell-type-specific labeling techniques (see Discussion) will certainly be useful to address this issue in the future.

As Ca^{2+}-regulated and synapse-to-nucleus signaling play a prominent role in all forms of plasticity, including homeostatic plasticity, we also searched for altered protein synthesis in these pathways (Figure 7; Table S4). The CamKII family of subunits, for example, showed a consistent trend of upregulated synthesis following scaling-up and downregulated synthesis following scaling-down (Figure 7; Table S2). A similar pattern was observed for the beta subunit of Protein kinase C (Prkcb) and several of the Map kinases (Mapk3, Map4k, and Mapk8) (Figure 7). The inositol trisphosphate receptor 2 (ITPR2) was also potently and significantly downregulated with both forms of plasticity, whereas the ITPR1 molecule was significantly downregulated exclusively with scaling-up (Figure 7). The family of Arhgaps, negative regulators of Rho GTPases, together with the positive regulator of Rho GTPases and dendritic morphology regulator Tiam1 (Um et al., 2014), represent a hotspot for significant regulation during homeostatic scaling.

In the above sections, we highlighted some proteins that are altered during synaptic scaling. As defects in synaptic scaling have been proposed to contribute to diseases such as

Schizophrenia (71 genes)

Abcb1, Acly, Adarb1, Adrbk1, Ap3b2, Ap3m2, Arhgef11, Arvcf, Asns, Atn1, Bag3, Brd1, Cadm3, Cpne3, Crh, Csnk1e, Exoc4, Fbxo41, Fgfr2, Fos, Fyn, Ggnbp2, Gigyf2, Gria3, Grid1, Grik2, Hars2, Hdac4, Hsd11b1, Kif3c, Kif5a, Kifap3, Lamb2, Lpl, Macf1, Mapk3, Mthfd1, Mt-Nd5, Nck1, Nfkb1, Ngef, Nlgn2, Nt5c2, Ntrk3, Nvl, Pard6a, Pik3ca, Pja1, Psen1, Psen2, Ptprg, Ptprn2, Rb1cc1, Rft1, Rnh1, Rptor, Slc12a5, Smarcc1, Snap29, Snx29, Sptbn4, Stk24, Synrg, Tagln3, Tamm41, Tbcd, Tnr, Trim3, Usp4, Utrn, Wwox

Alzheimer´s disease (66 genes)

Abcb1, Adap1, Adrbk1, Ank1, Arhgap12, Arhgap21, Arvcf, Bag3, Bid, Crh, Dip2c, Dnajc13, Dock3, Epn2, Exoc4, Fgfr2, Fos, Fyn, Gria3, Grid1, Grik2, Hpcal1, Hsd11b1, Igf2r, Insr, Itpr1, Itpr2, Kif11, Lemd2, Lpl, Madd, Mapk3, Mapk8ip1, Mms19, Mthfd1, Mt-Nd5, Nck1, Nckipsd, Ncoa4, Nfkb1, Nt5c2, Ntrk3, Ogt, Parvb, Pigq, Pik3ca, Plekha6, Plod1, Ppp1r8, Prkcb, Prune2, Psen1, Psen2, Pxn, Ralb, Rb1cc1, Rcan2, Rrp1, Samd8, Scg5, Slc30a5, Slk, Smpd2, Stk24, Ubxn4, Wwox

Epilepsy (60 genes)

Abcb1, Ank1, Ap3m2, Appl2, Arvcf, Asns, Atg7, Atn1, Axl, Cacnb4, Chd1, Crh, Cul4b, Etl4, Fgfr2, Flrt3, Fos, Ggnbp2, Gna11, Gria3, Grik2, Hars2, Hdac4, Igf2r, Insr, Kif11, Kif5a, Kirrel3, Lamb2, Lss, Mapk3, Mpi, Mthfd1, Mt-Nd5, Ndst1, Nedd4l, Nfkb1, Nipa1, Nop2, Pigq, Pik3ca, Pnpla6, Pogz, Prkar1b, Prkcb, Psen1, Psen2, Ranbp2, Rb1cc1, Rft1, Rptor, Slc12a5, Slc25a17, Smad1, Smarcc2, Snap29, Synrg, Tbcd, Wwox, Zfp36l1

Parkinson´s disease (39 genes)

Abcb1, Adrbk1, Atg7, Atn1, Bag3, Bid, Coasy, Crh, Daam1, Ddx41, Dnajc13, Dock3, Fbxo41, Fbxo42, Fyn, Gigyf2, Gria3, Grik2, Igf2r, Kif11, Kif5a, Kifap3, Lpl, Map4k4, Mapk3, Mapk8ip1, Mthfd1, Mt-Nd5, Nfkb1, Ogt, Pik3ca, Psen1, Psen2, Rptor, Slc2a13, Tiam1, Usp24, Vangl1, Wwox

Autism (37 genes)

Abcb1, Amot, Appl2, Arvcf, Csnk1e, Dnpep, Dock3, Fmnl2, Gcc1, Ggnbp2, Gria3, Grik2, Hdac4, Hsd11b1, Kirrel3, Mapk3, Mdga2, Mrpl19, Mthfd1, Mt-Nd5, Necab2, Nfkb1, Ngef, Nlgn2, Ntrk3, Pigq, Pik3ca, Pogz, Prkcb, Psen1, Pxn, Scg5, Slc12a5, Smarcc2, Snap29, Synrg, Wwox

Amyotrophic lateral sclerosis (30 genes)

Adarb1, Amot, Ank1, Atn1, Bid, Dbr1, Fos, Gria3, Grik2, Hars2, Hdac4, Igf2r, Itpr2, Kifap3, Lpl, Lrrc8c, Mthfd1, Mt-Nd5, Nedd4l, Nfkb1, Nipa1, Psen1, Psen2, Raver1, Slk, Tiam1, Txnl4b, Ubap2, Utrn, Uxt

Bipolar disorder (24 genes)

Abcb1, Adrbk1, Arvcf, Asns, Brd1, Csnk1e, Fgfr2, Fos, Fyn, Gria3, Grid1, Grik2, Mapk3, Mthfd1, Mt-Nd5, Nt5c2, Ntrk3, Pard6b, Ptprn2, Smpd2, Stk24, Wwox, Crh, Rft1

Multiple sclerosis (18 genes)

Abcb1, Adrbk1, Axl, Crh, Gas6, Hbegf, Igf2r, Kif5a, Kif21b, Lpl, Mms19, Mt-Nd5, Nfkb1, Nipa1, Smpd2, Tnr, Utrn, Zfp36l1

Huntington´s disease (15)

Atg7, Atn1, Evl, Fgfr2, Gna11, Grik2, Hdac4, Itpr1, Itpr2, Nfkb1, Polr2a, Polr2e, Ppp1r21, Prkcb, Qrich1

Depression (8)

Abcb1, Adrbk1, Crh, Fgfr2, Fos, Nvl, Ptprg, Rasa1

FTD (8)

Ank1, Atg7, Grik2, Kifap3, Lpl, Map4k4, Psen1, Psen2

Figure 8. Neurological Disease-Associated Proteins Regulated by Homeostatic Scaling

Shown are genes of significantly regulated proteins associated with each of the indicated diseases. Of the 325 genes regulated by scaling induction, 166 are associated with one of the diseases shown. Some genes are listed for multiple diseases.

Alzheimer's disease (Pratt et al., 2011) and epilepsy (Trasande and Ramirez, 2007), we asked whether any of the regulated proteins identified in this study are associated with neurodevelopmental, psychiatric, or neurodegenerative disorders. Forty-five percent of the proteins regulated during homeostatic scaling had an association with a neural disease. As shown in Figure 8, we queried regulated proteins associated with Alzheimer's disease, Parkinson's disease, Huntington's disease, schizophrenia, epilepsy, autism, amyotrophic lateral sclerosis, multiple sclerosis, bipolar disorder, depression, and frontotemporal dementia (see also Table S7) and found over 166 proteins that were associated with at least one of the above diseases. Within this set of disease-related regulated proteins, we identified that, for example, Presenilin-1 (Psen), previously reported to be essential for homeostatic scaling (Pratt et al., 2011), is significantly regulated in both up- and down-scaling (Figures 6 and 8; Table S2). We additionally examined the association of our regulated proteins with disease-related single-nucleotide polymorphisms identified in genome-wide association studies (GWAS) (Table S7). Here we again found that a large number (~95) of the proteins regulated by synaptic homeostasis are associated with neurological dysfunction.

DISCUSSION

In this study, we examined the proteomic changes that underlie homeostatic scaling in primary hippocampal neurons. We demonstrate for the first time that both up- and down-scaling elicited by pharmacological manipulation of neuronal activity require new protein synthesis. Using BONCAT (Dieterich et al., 2006), we selectively labeled, purified, and analyzed the newly synthesized neuronal proteomes. Here, BONCAT enabled us to track and visualize proteome adaptations during homeostatic scaling that are usually extremely challenging to examine in tissue samples due to both the dynamic range of the proteome and the relatively small degree of differential regulation. We identified over 5,000 proteins in each experimental group as well as the control group. Homeostatic scaling did not alter the absolute number of distinct newly synthesized proteins, but rather resulted in differences in which proteins were synthesized and to what degree. We detected ~300 proteins that exhibited significantly altered protein synthesis as a result of the global activity manipulations that induce homeostatic scaling. Recently, other groups have combined BONCAT with SILAC in order to include an additional validation step for new protein synthesis and to enable isotope-based, robust quantification (Bagert et al., 2014; Eichelbaum and Krijgsveld, 2014). Here we circumvented the need for SILAC, applying stringent control experiments and analyses and a highly stable LC-MS/MS setup, producing the largest dataset for the newly synthesized neuronal proteomes to date. We also investigated "low-abundance" proteins that are notoriously challenging to quantify due to the limited number of detectable peptides or low peptide intensities. We employed a combination of reproducibility, spectral counting, and normalized peptide intensities to manually evaluate a subset of low-abundance proteins, and as such these evaluations are "semi-quantitative"; the derived lists represent "trends" that may be of interest.

The significantly regulated newly synthesized proteins we identified span many functional categories and reside in different cellular compartments. Because of the time course of the plasticity and the duration of the metabolic labeling (24 hr), these proteins can represent regulated "sensors," which respond to the activity manipulation, or "effectors," which drive the

implementation of the homeostatic responses. In addition to these proteins, we explicitly evaluated the previously published data documenting roles for various proteins in homeostatic scaling (Table S5). Of the 29 scaling-related proteins we identified in the literature, we detected 20 as newly synthesized in our dataset (~69%). Of these 20 proteins, we detected either a trend for regulation or significant regulation associated with scaling for 65% percent. We also queried the extent to which our significantly regulated proteins have been associated with various neurological diseases and found that a large fraction (166 genes, ~51%) have been implicated in one or more neurological diseases. An emerging view that incorporates the profound cumulative understanding that many neurological diseases are associated with mutations in hundreds to thousands of proteins is the notion that a loss of homeostasis at the level of neural circuits is causal (Ramocki and Zoghbi, 2008). The number of proteins identified and regulated by homeostatic scaling here provides a starting point to examine how dysregulation of these "homeostatic" proteins contributes to a variety of neuronal disorders.

Are the two forms of plasticity, homeostatic up-scaling and down-scaling, on a single mechanistic continuum, or are orthogonal mechanisms used? Most mechanistic studies have focused on up-scaling, examining the role of molecules that are associated with AMPA receptor trafficking or stabilization. A general question is whether the same mechanisms run in reverse for down-scaling. We observed multiple protein families and regulatory pathways that exhibited opposite regulation depending on the sign of the scaling (Figures 4 and S3), suggesting that these two forms of plasticity can be mediated by differential regulation of a common molecular-protein continuum. These protein families include, for example, the ionotropic glutamate receptor complex, proteins associated with neurotransmitter transport and exocytosis, and transcription factors. These data support the idea that at least some of the opposite phenotypes elicited by up- and down-scaling are driven mechanistically by an overlapping cast of molecular players, regulated in different directions. If the global manipulations of activity used here and in other studies possess a common sensor (e.g., Ca^{2+}), then the downstream mechanisms that bring about scaling-up or scaling-down could activate Ca^{2+} sensors with different sensitivities (e.g., Sutton et al., 2007) to bring about a concerted protein synthesis response.

A central question for all forms of cellular plasticity, of course, is the degree to which phenotypic changes are driven by changes in transcription and/or translation. A previous study indicated a requirement for transcription in homeostatic up-scaling (Ibata et al., 2008). Here we show for the first time that inhibition of protein synthesis during the initial 12 hr of scaling induction blocks both scaling-up and scaling-down. The period of protein synthesis inhibition was brief relative to the half-life of most neuronal/synaptic proteins (median = ~3.5 days; Cohen et al., 2013), and there was no effect on baseline mEPSC frequency or amplitude. We thus conclude that the observed inhibition of scaling reflects a specific requirement for newly synthesized proteins in the expression of homeostatic scaling. Whether the observed changes in protein synthesis are due to changes in translation specifically or accompanied by changes in transcript level is an interesting question. In our data, there are several examples where multiple elements of a common signaling cascade are regulated (e.g., Figure 7). An open issue concerns the transcriptional or translational regulatory mechanisms that coordinate the changes in neuronal compartments so that elements in the same signaling pathway are consistently regulated to bring about change at the end-point, e.g., a positive or negative modulation of synaptic strength. In addition, although it has not been explicitly addressed here, it is obvious that the regulated degradation of proteins must also play a role in sculpting the proteome during homeostatic scaling. Indeed, we observed many proteins in the ubiquitin proteasome pathway that were regulated by scaling (Figure 3C; Table S3).

In addition to the proteomic changes described here, protein degradation, protein modifications, and/or transient protein complex formation play critical roles in adjusting synaptic strength. Indeed, Ehlers (2003) identified a cohort of synaptic proteins that were differentially regulated by activity manipulations and noted that inhibition of the ubiquitin proteasome pathway blocked the differential regulation. In addition, fast adaptions, such as short-term potentiation or depression, often rely heavily on modifications of existing proteins. In support of this idea, "kinases/phosphatases" represent the largest differentially regulated group in our dataset (Figure 3C; Table S3). For example, we found multiple MAP kinases and associated proteins (Table S3) that play a central role in multiple cellular processes. Also, our String analysis revealed that the tyrosine-protein kinases Lyn and Fyn represent central interaction "hubs" of the differentially regulated proteins in our dataset. Accordingly, future studies have to focus on obtaining complementary data on post-translational modifications and transient complex formation during homeostatic scaling.

We used BONCAT in hippocampal cultures to discover the nascent proteomic response to both up- and down-scaling. As the non-canonical amino acid AHA can be charged by the wild-type MetRS, all cells present in the culture can, in theory, contribute to the newly synthesized proteins we analyzed. Using bioinformatics, we were able to approximate attributions of some proteins to glia or interneurons, but this type of analysis is clearly limited. For example, it has been shown that homeostatic scaling can also be elicited at inhibitory synapses (Hartman et al., 2006). As such, common proteins in excitatory and inhibitory neurons that exhibit regulation in opposite directions might "cancel each other out" and hence not be detected as significantly regulated in our study. To gain selective access to particular cell classes, a recent development in which a mutant MetRS enables cell-type-specific labeling could be used in future experiments. This has already been employed in both *C. elegans* (Yuet et al., 2015) and *Drosophila* (Erdmann et al., 2015) to monitor protein synthesis in specific cell types. Applying this technology to homeostatic scaling and other forms of plasticity will allow us to discern the coordinated proteomic response in the different cell types that make up neuronal circuits.

EXPERIMENTAL PROCEDURES

Preparation and Maintenance of Cultured Neurons

Dissociated hippocampal neurons were prepared and maintained as previously described (Aakalu et al., 2001). Briefly, hippocampi from postnatal day

0–2 rat pups (strain Sprague-Dawley) were dissected out and dissociated by either trypsin or papain and plated at a density of 40,000 cells/cm^2 onto poly-D-lysine-coated glass-bottom Petri dishes (MatTek). Cultures were maintained in Neurobasal A medium containing B-27 and Glutamax supplements (Invitrogen) at 37°C for 21 days before experiments commenced. All experiments were carried out with the approval of the German animal experiment authorities.

Electrophysiology

Whole-cell recordings were performed in hippocampal culture neurons (DIV 21–27, 40 K cell density) held at −70 mV in voltage clamp mode. To induce homeostatic plasticity, neurons were pretreated with either bicuculline (40 μM) or TTX (2 μM) for 24 hr. To block protein synthesis, neurons were incubated in anisomycin (40 mM) for the first 12 hr of the treatment. mEPSCs were recorded for at least 10 min in extracellular solution containing 140 mM NaCl, 3 mM KCl, 10 mM HEPES, 2 mM $CaCl_2$, 1 mM $MgSO_4$, 15 mM glucose, 2 μM TTX, and 40 μM bicuculline (pH 7.4). Recording pipettes (resistances 3–8 MΩ) contained 120 mM potassium gluconate, 20 mM KCl, 10 mM HEPES, 2 mM $MgCl_2$, 0.1 mM EGTA, 2 mM Na_2-ATP, and 0.4 mM Na_2-GTP (300 mOsm/L, pH 7.2). Data were analyzed offline in MATLAB using a custom-made template-matching algorithm. Selected mEPSC events were individually screened with an amplitude threshold of > 5 pA and an exponential decay. Statistics were conducted using unpaired t tests.

Sample Preparation and MS Analyses

Dissociated hippocampal neurons (DIV 21, two dishes per condition, ~800,000 cells in total) were incubated with 4 mM AHA (or methionine for the control group) and treated with bicuculline (20 μM) or tetrodotoxin (1 μM) for 24 hr. After incubation, the cells were washed with cold DPBS containing protease inhibitor cocktails (cOmplete EDTA-free, Roche), harvested, snap-frozen in liquid nitrogen, and stored at −80°C until purification.

For cell lysis, the samples (cell pellets) were centrifuged briefly, the pellets resuspended in lysis buffer (100 μL, 8 M urea, 200 mM Tris [pH 8.4], 4% CHAPS, 1 M NaCl, cOmplete EDTA-free protease inhibitor), and lysed using a pistil. After sonication (4 × 30 s at 4°C), benzonase (1 μL of a ≥ 250 units/μL stock solution) was added to the solubilized samples, incubated for 5 min, and centrifuged for 5 min at 10,000× *g*. The supernatants (~200 μL) were subsequently incubated with freshly prepared catalyst solution (250 μL) and Alkyne-Sepharose slurry (50 μL; see manufacturer's protocol, Thermo Fisher Scientific) and incubated for 19 hr in the dark under gentle agitation.

After a short centrifugation (2 min, 1,000× *g*), the beads were washed twice (900 μL each) and incubated with SDS wash buffer (100 mM Tris [pH 8], 1% SDS, 250 mM NaCl, 5 mM EDTA) containing 10 mM TCEP (250 μL). After incubation for 45 min at 55°C under gentle agitation, the samples were centrifuged for 5 min at 1,000× *g* and the supernatants discarded. Each sample was then incubated with 250 μL SDS wash buffer containing a final concentration of 95 mM iodoacetamide for 30 min at room temperature in the dark under gentle agitation.

The samples were then transferred into pre-washed (400 μL H_2O, LC-MS/MS-ChromaSolv; 400 μL SDS wash buffer) Pierce Spin columns and washed with 20 mL SDS wash buffer, 20 mL 8 M urea in 100 mM Tris (pH 8), and 20 mL 20% (v/v) acetonitrile/water.

After washing, 250 μL of digestion buffer (100 mM Tris, 2 mM $CaCl_2$, 10% acetonitrile) was added to each column and the resin transferred to an Eppendorf tube. After one washing step with digestion buffer, ~0.65 μg of EndoLysC was added to each tube and incubated overnight (~22 hr) at 37°C with constant agitation. For the second digestion step, each sample was incubated with 0.65 μg trypsin at 37°C overnight (~22 hr) with constant agitation.

The proteolytic peptides were extracted by incubating the resin with 500 μL H_2O (0.1% TFA), centrifuging for 5 min at 1,000× *g*, and collecting the supernatant. This step was repeated, and the combined supernatants were desalted using c_{18}-SepPak columns (Waters Corp.). The SepPak resin was washed with 2 mL of 50% acetonitrile/water (5% acetic acid) and equilibrated using 2 mL water (0.1% TFA). Each sample (~1 mL) was loaded onto a column and washed with 2 mL of water (0.1% TFA) and 200 μL of water (0.5% acetic acid). Peptides were eluted using 0.5 mL of 50% acetonitrile/water (0.5% acetic acid), dried using a Speed-Vac, and stored at −80°C until LC-MS/MS analysis.

For LC-MS/MS injection, the dried peptide fractions were dissolved in 5% acetonitrile with 0.1% formic acid, and subsequently loaded using a nano-HPLC (Dionex U3000 RSLCnano) on reverse-phase columns (trapping column: particle size 3 μm, C18, L = 20 mm; analytical column: particle size < 2 μm, C18, L = 50 cm; PepMap, Dionex/Thermo Fisher Scientific). Peptides were eluted in gradients of water (buffer A: water with 5% [v/v] acetonitrile and 0.1% formic acid) and acetonitrile (buffer B: 20% [v/v] water, 80% [v/v] acetonitrile, and 0.1% formic acid). All LC-MS/MS-grade solvents were purchased from Fluka. Gradients were ramped from 4% to 48% B in 178 min at flowrates of 300 nL/min. Peptides eluting from the column were ionized online using a Thermo nanoFlex ESI source and analyzed in a Thermo "Q Exactive Plus" mass spectrometer. Mass spectra were acquired over the mass range 350–1,400 m/z, and sequence information was acquired by computer-controlled, data-dependent automated switching to MS/MS mode using collision energies based on mass and charge state of the candidate ions (TOP12, MS resolution 70 k, MS^2 resolution 35 k, injection time: 120 ms, full parameter set in Table S9). All samples were measured in quadruplicate LC-MS/MS runs.

Bioinformatic Processing and Criteria

Raw data were loaded into MaxQuant (version 1.5.3.8; Cox and Mann, 2008) and analyzed with a customized Andromeda LFQ parameter set (see Table S9). In brief, spectra were matched to a *Rattus norvegicus* database downloaded from uniprot.org (34,170 entries, reviewed and non-reviewed) and a contaminant and decoy database. Precursor mass tolerance was set to 20 ppm, fragment ion tolerance to 4.5 ppm, with fixed modification of Cys residues (carboxyamidomethylation +57.021) and variable modifications of Met residues (Ox +15.995), Lys residues (Acetyl +42.011), Asn and Gln residues (Deamidation +0.984), and N termini (carbamylation +43.006). Peptide identifications were calculated with FDR = 0.01, and proteins with one peptide per protein were included for subsequent analyses.

Five independent biological replicates were processed for each group (methionine control, untreated cells, bicuculline-treated cells, tetrodotoxin-treated cells). After subtraction of background proteins, protein lists for each group were compiled containing proteins identified in at least 2 out of 5 replicates. The methionine content of our AHA-labeled proteins was not different compared to a global proteome (*Rattus norvegicus*, Uniprot database) as, on average, 2.38 and 2.40 met residues are present per 100 amino acids, respectively.

For full details on the data processing, please see Supplemental Experimental Procedures.

ACCESSION NUMBERS

The proteomics data associated with this manuscript have been uploaded to the PRIDE online repository: PXD004889 (Vizcaíno et al., 2016).

SUPPLEMENTAL INFORMATION

Supplemental Information includes Supplemental Experimental Procedures, five figures, and nine tables and can be found with this article online at http://dx.doi.org/10.1016/j.neuron.2016.09.058.

AUTHOR CONTRIBUTIONS

C.T.S., J.D.L., and E.M.S. designed experiments; C.T.S., S.S., and J.D.L. conducted and analyzed experiments. E.M.S. wrote the manuscript; all authors edited and revised the manuscript.

ACKNOWLEDGMENTS

We thank I. Bartnik, N. Fuerst, A. Staab, and C. Thum for the preparation of cultured hippocampal neurons and Imke Wüllenweber for excellent technical assistance. E.M.S. is funded by the Max Planck Society, an Advanced

Investigator award from the European Research Council, and DFG CRC 1080: Molecular and Cellular Mechanisms of Neural Homeostasis, DFG CRC 902: Molecular Principles of RNA-based Regulation, and the DFG Cluster of Excellence for Macromolecular Complexes, Goethe University.

Received: August 2, 2016
Revised: September 16, 2016
Accepted: September 27, 2016
Published: October 19, 2016

REFERENCES

Aakalu, G., Smith, W.B., Nguyen, N., Jiang, C., and Schuman, E.M. (2001). Dynamic visualization of local protein synthesis in hippocampal neurons. Neuron *30*, 489–502.

Anggono, V., Clem, R.L., and Huganir, R.L. (2011). PICK1 loss of function occludes homeostatic synaptic scaling. J. Neurosci. *31*, 2188–2196.

Bagert, J.D., Xie, Y.J., Sweredoski, M.J., Qi, Y., Hess, S., Schuman, E.M., and Tirrell, D.A. (2014). Quantitative, time-resolved proteomic analysis by combining bioorthogonal noncanonical amino acid tagging and pulsed stable isotope labeling by amino acids in cell culture. Mol. Cell. Proteomics *13*, 1352–1358.

Bowling, H., Bhattacharya, A., Zhang, G., Lebowitz, J.Z., Alam, D., Smith, P.T., Kirshenbaum, K., Neubert, T.A., Vogel, C., Chao, M.V., and Klann, E. (2016). BONLAC: A combinatorial proteomic technique to measure stimulus-induced translational profiles in brain slices. Neuropharmacology *100*, 76–89.

Burrone, J., O'Byrne, M., and Murthy, V.N. (2002). Multiple forms of synaptic plasticity triggered by selective suppression of activity in individual neurons. Nature *420*, 414–418.

Cajigas, I.J., Tushev, G., Will, T.J., tom Dieck, S., Fuerst, N., and Schuman, E.M. (2012). The local transcriptome in the synaptic neuropil revealed by deep sequencing and high-resolution imaging. Neuron *74*, 453–466.

Cingolani, L.A., Thalhammer, A., Yu, L.M., Catalano, M., Ramos, T., Colicos, M.A., and Goda, Y. (2008). Activity-dependent regulation of synaptic AMPA receptor composition and abundance by beta3 integrins. Neuron *58*, 749–762.

Cohen, L.D., Zuchman, R., Sorokina, O., Müller, A., Dieterich, D.C., Armstrong, J.D., Ziv, T., and Ziv, N.E. (2013). Metabolic turnover of synaptic proteins: kinetics, interdependencies and implications for synaptic maintenance. PLoS ONE *8*, e63191.

Cox, J., and Mann, M. (2008). MaxQuant enables high peptide identification rates, individualized p.p.b.-range mass accuracies and proteome-wide protein quantification. Nat. Biotechnol. *26*, 1367–1372.

Davis, G.W. (2013). Homeostatic signaling and the stabilization of neural function. Neuron *80*, 718–728.

Desai, N.S., Cudmore, R.H., Nelson, S.B., and Turrigiano, G.G. (2002). Critical periods for experience-dependent synaptic scaling in visual cortex. Nat. Neurosci. *5*, 783–789.

Dieterich, D.C., Link, A.J., Graumann, J., Tirrell, D.A., and Schuman, E.M. (2006). Selective identification of newly synthesized proteins in mammalian cells using bioorthogonal noncanonical amino acid tagging (BONCAT). Proc. Natl. Acad. Sci. USA *103*, 9482–9487.

Dieterich, D.C., Lee, J.J., Link, A.J., Graumann, J., Tirrell, D.A., and Schuman, E.M. (2007). Labeling, detection and identification of newly synthesized proteomes with bioorthogonal non-canonical amino-acid tagging. Nat. Protoc. *2*, 532–540.

Distler, U., Schmeisser, M.J., Pelosi, A., Reim, D., Kuharev, J., Weiczner, R., Baumgart, J., Boeckers, T.M., Nitsch, R., Vogt, J., and Tenzer, S. (2014). In-depth protein profiling of the postsynaptic density from mouse hippocampus using data-independent acquisition proteomics. Proteomics *14*, 2607–2613.

Ehlers, M.D. (2003). Activity level controls postsynaptic composition and signaling via the ubiquitin-proteasome system. Nat. Neurosci. *6*, 231–242.

Eichelbaum, K., and Krijgsveld, J. (2014). Rapid temporal dynamics of transcription, protein synthesis, and secretion during macrophage activation. Mol. Cell. Proteomics *13*, 792–810.

Erdmann, I., Marter, K., Kobler, O., Niehues, S., Abele, J., Müller, A., Bussmann, J., Storkebaum, E., Ziv, T., Thomas, U., and Dieterich, D.C. (2015). Cell-selective labelling of proteomes in Drosophila melanogaster. Nat. Commun. *6*, 7521.

Gainey, M.A., Hurvitz-Wolff, J.R., Lambo, M.E., and Turrigiano, G.G. (2009). Synaptic scaling requires the GluR2 subunit of the AMPA receptor. J. Neurosci. *29*, 6479–6489.

Gainey, M.A., Tatavarty, V., Nahmani, M., Lin, H., and Turrigiano, G.G. (2015). Activity-dependent synaptic GRIP1 accumulation drives synaptic scaling up in response to action potential blockade. Proc. Natl. Acad. Sci. USA *112*, E3590–E3599.

Goddard, C.A., Butts, D.A., and Shatz, C.J. (2007). Regulation of CNS synapses by neuronal MHC class I. Proc. Natl. Acad. Sci. USA *104*, 6828–6833.

Goold, C.P., and Nicoll, R.A. (2010). Single-cell optogenetic excitation drives homeostatic synaptic depression. Neuron *68*, 512–528.

Gygi, S.P., Rochon, Y., Franza, B.R., and Aebersold, R. (1999). Correlation between protein and mRNA abundance in yeast. Mol. Cell. Biol. *19*, 1720–1730.

Hartman, K.N., Pal, S.K., Burrone, J., and Murthy, V.N. (2006). Activity-dependent regulation of inhibitory synaptic transmission in hippocampal neurons. Nat. Neurosci. *9*, 642–649.

Hodas, J.J., Nehring, A., Höche, N., Sweredoski, M.J., Pielot, R., Hess, S., Tirrell, D.A., Dieterich, D.C., and Schuman, E.M. (2012). Dopaminergic modulation of the hippocampal neuropil proteome identified by bioorthogonal noncanonical amino acid tagging (BONCAT). Proteomics *12*, 2464–2476.

Ibata, K., Sun, Q., and Turrigiano, G.G. (2008). Rapid synaptic scaling induced by changes in postsynaptic firing. Neuron *57*, 819–826.

Jovanovic, M., Rooney, M.S., Mertins, P., Przybylski, D., Chevrier, N., Satija, R., Rodriguez, E.H., Fields, A.P., Schwartz, S., Raychowdhury, R., et al. (2015). Immunogenetics. Dynamic profiling of the protein life cycle in response to pathogens. Science *347*, 1259038.

Kilman, V., van Rossum, M.C., and Turrigiano, G.G. (2002). Activity deprivation reduces miniature IPSC amplitude by decreasing the number of postsynaptic GABA(A) receptors clustered at neocortical synapses. J. Neurosci. *22*, 1328–1337.

Komatsu, S., and Hossain, Z. (2013). Organ-specific proteome analysis for identification of abiotic stress response mechanism in crop. Front. Plant Sci. *4*, 71.

Kumar, R.A., KaraMohamed, S., Sudi, J., Conrad, D.F., Brune, C., Badner, J.A., Gilliam, T.C., Nowak, N.J., Cook, E.H., Jr., Dobyns, W.B., and Christian, S.L. (2008). Recurrent 16p11.2 microdeletions in autism. Hum. Mol. Genet. *17*, 628–638.

Landgraf, P., Antileo, E.R., Schuman, E.M., and Dieterich, D.C. (2015). BONCAT: metabolic labeling, click chemistry, and affinity purification of newly synthesized proteomes. Methods Mol. Biol. *1266*, 199–215.

Maffei, A., Nelson, S.B., and Turrigiano, G.G. (2004). Selective reconfiguration of layer 4 visual cortical circuitry by visual deprivation. Nat. Neurosci. *7*, 1353–1359.

Mayford, M., Barzilai, A., Keller, F., Schacher, S., and Kandel, E.R. (1992). Modulation of an NCAM-related adhesion molecule with long-term synaptic plasticity in Aplysia. Science *256*, 638–644.

Mazzucchelli, C., Vantaggiato, C., Ciamei, A., Fasano, S., Pakhotin, P., Krezel, W., Welzl, H., Wolfer, D.P., Pagès, G., Valverde, O., et al. (2002). Knockout of ERK1 MAP kinase enhances synaptic plasticity in the striatum and facilitates striatal-mediated learning and memory. Neuron *34*, 807–820.

Meadows, J.P., Guzman-Karlsson, M.C., Phillips, S., Holleman, C., Posey, J.L., Day, J.J., Hablitz, J.J., and Sweatt, J.D. (2015). DNA methylation regulates neuronal glutamatergic synaptic scaling. Sci. Signal. *8*, ra61.

Nakajima, K., Yin, X., Takei, Y., Seog, D.H., Homma, N., and Hirokawa, N. (2012). Molecular motor KIF5A is essential for GABA(A) receptor transport, and KIF5A deletion causes epilepsy. Neuron *76*, 945–961.

Niethammer, M., Valtschanoff, J.G., Kapoor, T.M., Allison, D.W., Weinberg, R.J., Craig, A.M., and Sheng, M. (1998). CRIPT, a novel postsynaptic protein that binds to the third PDZ domain of PSD-95/SAP90. Neuron *20*, 693–707.

O'Brien, R.J., Kamboj, S., Ehlers, M.D., Rosen, K.R., Fischbach, G.D., and Huganir, R.L. (1998). Activity-dependent modulation of synaptic AMPA receptor accumulation. Neuron *21*, 1067–1078.

Peng, J., Kim, M.J., Cheng, D., Duong, D.M., Gygi, S.P., and Sheng, M. (2004). Semiquantitative proteomic analysis of rat forebrain postsynaptic density fractions by mass spectrometry. J. Biol. Chem. *279*, 21003–21011.

Pielot, R., Smalla, K.H., Müller, A., Landgraf, P., Lehmann, A.C., Eisenschmidt, E., Haus, U.U., Weismantel, R., Gundelfinger, E.D., and Dieterich, D.C. (2012). SynProt: A Database for Proteins of Detergent-Resistant Synaptic Protein Preparations. Front. Synaptic Neurosci. *4*, 1.

Pratt, K.G., Zimmerman, E.C., Cook, D.G., and Sullivan, J.M. (2011). Presenilin 1 regulates homeostatic synaptic scaling through Akt signaling. Nat. Neurosci. *14*, 1112–1114.

Ramocki, M.B., and Zoghbi, H.Y. (2008). Failure of neuronal homeostasis results in common neuropsychiatric phenotypes. Nature *455*, 912–918.

Rutherford, L.C., Nelson, S.B., and Turrigiano, G.G. (1998). BDNF has opposite effects on the quantal amplitude of pyramidal neuron and interneuron excitatory synapses. Neuron *21*, 521–530.

Schuster, C.M., Davis, G.W., Fetter, R.D., and Goodman, C.S. (1996). Genetic dissection of structural and functional components of synaptic plasticity. II. Fasciclin II controls presynaptic structural plasticity. Neuron *17*, 655–667.

Sharma, K., Schmitt, S., Bergner, C.G., Tyanova, S., Kannaiyan, N., Manrique-Hoyos, N., Kongi, K., Cantuti, L., Hanisch, U.K., Philips, M.A., et al. (2015). Cell type- and brain region-resolved mouse brain proteome. Nat. Neurosci. *18*, 1819–1831.

Shepherd, J.D., Rumbaugh, G., Wu, J., Chowdhury, S., Plath, N., Kuhl, D., Huganir, R.L., and Worley, P.F. (2006). Arc/Arg3.1 mediates homeostatic synaptic scaling of AMPA receptors. Neuron *52*, 475–484.

Steinmetz, C.C., and Turrigiano, G.G. (2010). Tumor necrosis factor-α signaling maintains the ability of cortical synapses to express synaptic scaling. J. Neurosci. *30*, 14685–14690.

Stellwagen, D., and Malenka, R.C. (2006). Synaptic scaling mediated by glial TNF-alpha. Nature *440*, 1054–1059.

Sun, Q., and Turrigiano, G.G. (2011). PSD-95 and PSD-93 play critical but distinct roles in synaptic scaling up and down. J. Neurosci. *31*, 6800–6808.

Sutton, M.A., Taylor, A.M., Ito, H.T., Pham, A., and Schuman, E.M. (2007). Postsynaptic decoding of neural activity: eEF2 as a biochemical sensor coupling miniature synaptic transmission to local protein synthesis. Neuron *55*, 648–661.

Thiagarajan, T.C., Lindskog, M., and Tsien, R.W. (2005). Adaptation to synaptic inactivity in hippocampal neurons. Neuron *47*, 725–737.

Trasande, C.A., and Ramirez, J.M. (2007). Activity deprivation leads to seizures in hippocampal slice cultures: is epilepsy the consequence of homeostatic plasticity? J. Clin. Neurophysiol. *24*, 154–164.

Turrigiano, G. (2012). Homeostatic synaptic plasticity: local and global mechanisms for stabilizing neuronal function. Cold Spring Harb. Perspect. Biol. *4*, a005736.

Turrigiano, G.G., Leslie, K.R., Desai, N.S., Rutherford, L.C., and Nelson, S.B. (1998). Activity-dependent scaling of quantal amplitude in neocortical neurons. Nature *391*, 892–896.

Um, K., Niu, S., Duman, J.G., Cheng, J.X., Tu, Y.K., Schwechter, B., Liu, F., Hiles, L., Narayanan, A.S., Ash, R.T., et al. (2014). Dynamic control of excitatory synapse development by a Rac1 GEF/GAP regulatory complex. Dev. Cell *29*, 701–715.

Vizcaíno, J.A., Csordas, A., del-Toro, N., Dianes, J.A., Griss, J., Lavidas, I., Mayer, G., Perez-Riverol, Y., Reisinger, F., Ternent, T., et al. (2016). 2016 update of the PRIDE database and its related tools. Nucleic Acids Res. *44* (D1), D447–D456.

Yuet, K.P., Doma, M.K., Ngo, J.T., Sweredoski, M.J., Graham, R.L., Moradian, A., Hess, S., Schuman, E.M., Sternberg, P.W., and Tirrell, D.A. (2015). Cell-specific proteomic analysis in Caenorhabditis elegans. Proc. Natl. Acad. Sci. USA *112*, 2705–2710.

Neuron, Volume *92*

Supplemental Information

Nascent Proteome Remodeling following Homeostatic Scaling at Hippocampal Synapses

Christoph T. Schanzenbächer, Sivakumar Sambandan, Julian D. Langer, and Erin M. Schuman

Schanzenbaecher et al., Supplemental Materials.

Contents:

Supplemental Figures 1-5.
Supplemental Figures 1-5 legends.
Supplemental Table legends.
Supplemental Table 2b.
Supplemental Table 8.
Supplemental Experimental Procedures.

Additional separate files:

Supplemental Table 1.
Supplemental Table 2a.
Supplemental Table 3.
Supplemental Table 4.
Supplemental Table 5.
Supplemental Table 6.
Supplemental Table 7.
Supplemental Table 9.

Supplemental Fig. 1

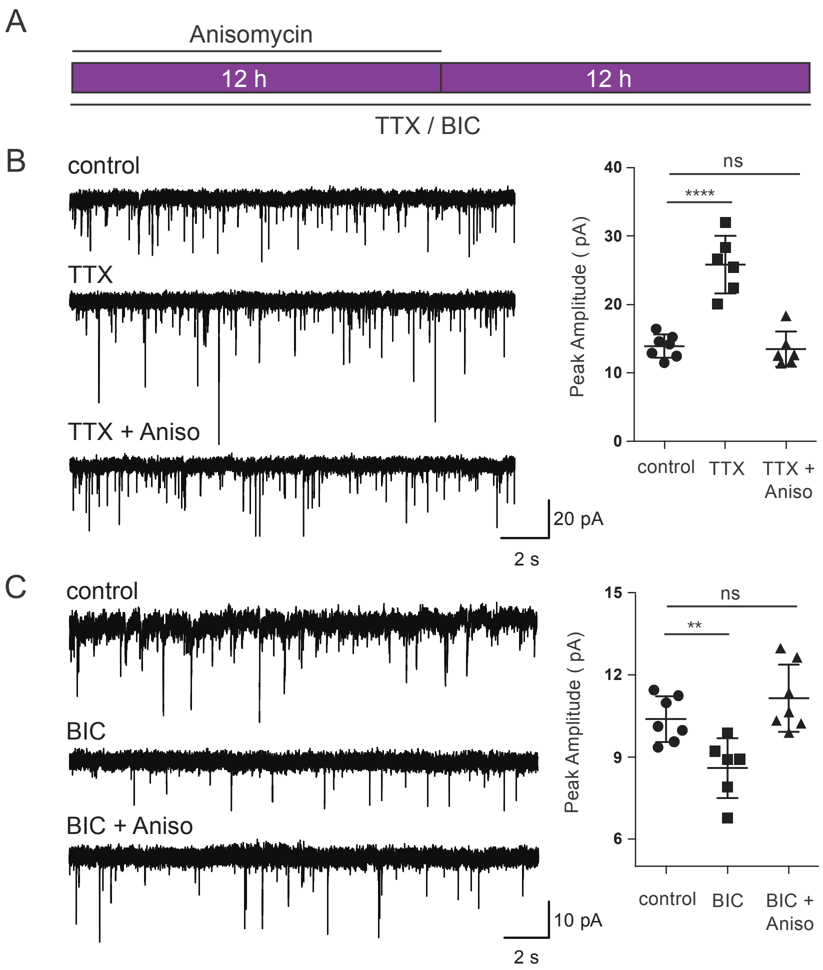

Supplemental Fig. 2

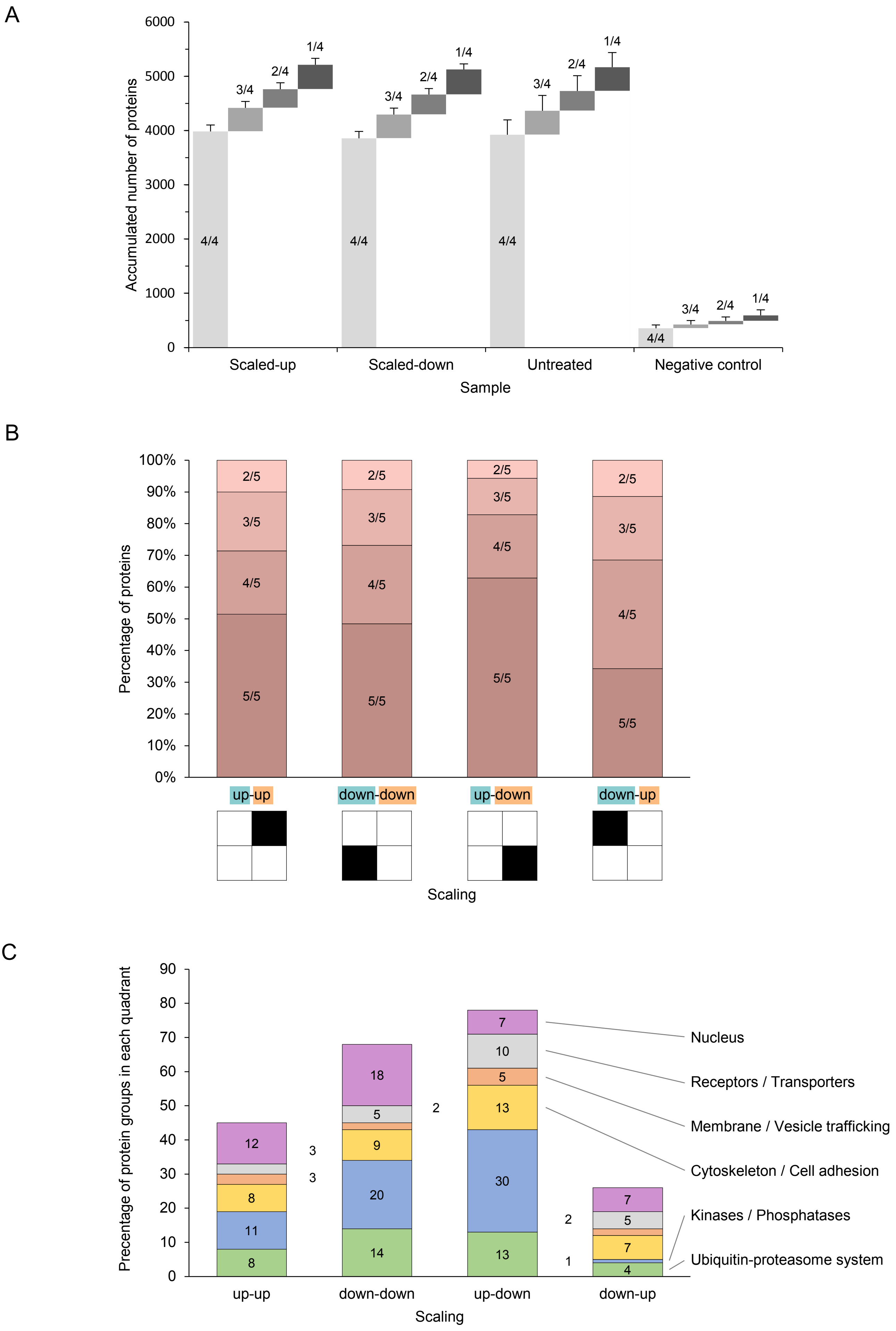

Supplemental Fig. 3

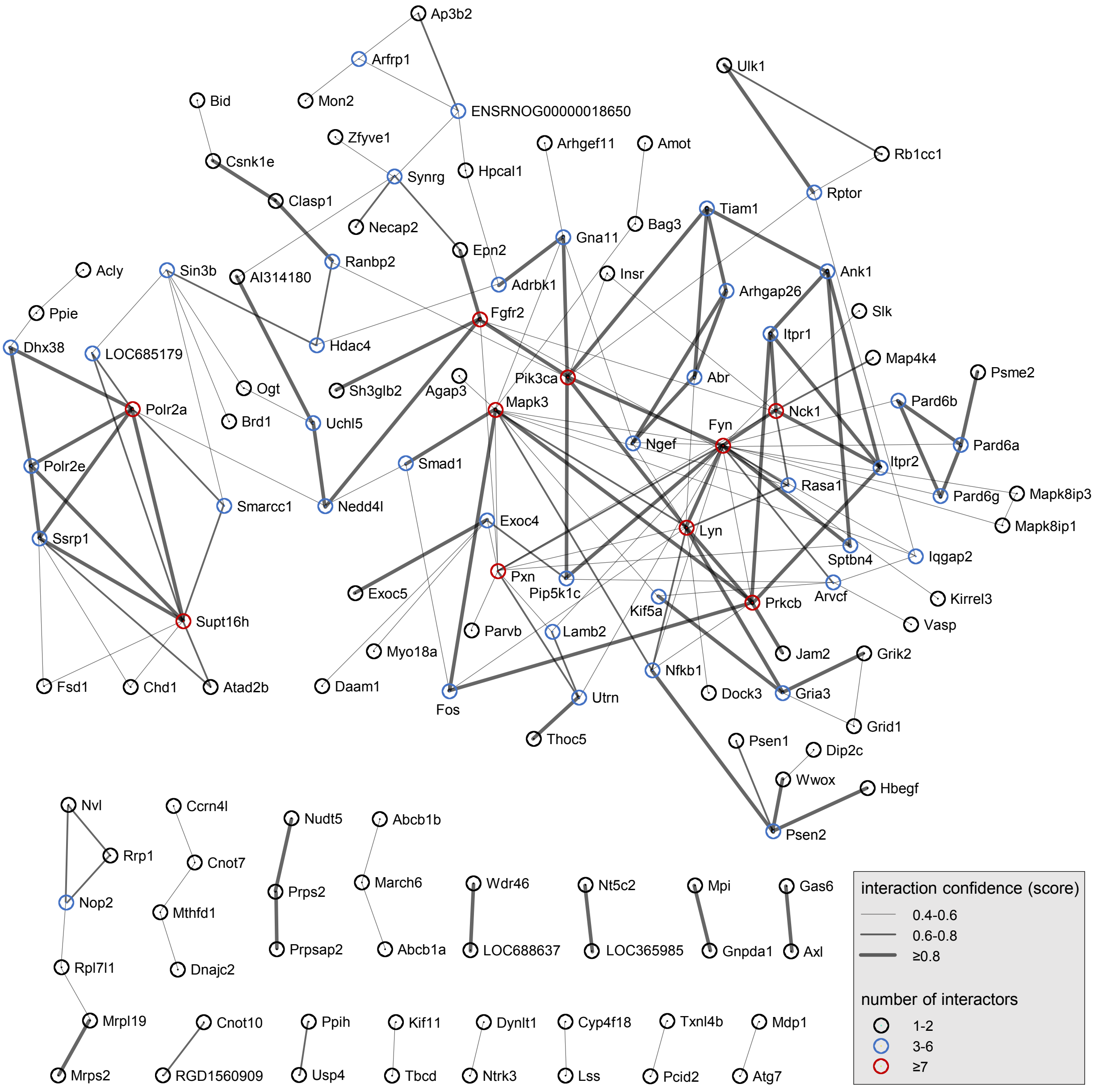

Adap1, Adarb1, Appl2, Arhgap12, Arhgap21, Asns, Atg2b, Atn1, Atpif1, Bcl7a, Birc6, Brsk1, C1ql3, Cacnb4, Cadm3, Caskin1, Ccdc51, Cdh11, Clasrp, Clcn4, Coasy, Commd3, Coro7, Cpne3, Crh, Cript, Ctbs, Cul4b, Dbr1, Ddhd2, Ddx41, Dnajc11, Dnajc13, Dnpep, Dos, Elfn2, ENSRNOG00000049462, Esrra, Esyt1, Etl4, Evl, Fam102a, Fam136a, Fam21c, Fbxl16, Fbxo41, Fbxo42, Fem1a, Flrt3, Fv1, Galnt1, Gcc1, Gdpd1, Ggnbp2, Gripap1, Hars2, Hid1, Hmgb3, Hsd11b1, Igf2r, Kif21b, Kif3c, Kifap3, Lactb, Lemd2, Lpl, Lrig1, Lrrc57, Lrrc7, Lrrc8a, Lrrc8c, Lyplal1, Macf1, Madd, Map4k3, Map7d2, Mdga2, Mms19l, Mospd1, Mtmr1, Nanp, Nckipsd, Ncoa4, ND5, Ndst1, Necab2, Nipa1, Nlgn2, Nol9, Npdc1, Phrf1, Phyhip, Pibf1, Pigq, Pinx1, Pja1, Plekha6, Plod1, Pnpla6, Pogz, Ppm1h, Ppp1r8, Prkar1b, Prune2, Ptpn9, Ptprg, Ptprn2, Qrich1, R3hdm1, Ralb, Rapgef2, Raver1, Rcan2, Rft1, RGD1559896, RGD1560248, Rnh1, Sall2, Samd8, Sbf1, Scai, Scg5, Secisbp2l, Sema6d, Setd3, Slc12a5, Slc25a17, Slc2a13, Slc30a5, Slc39a14, Smpd2, Snap29, Snx29, Sowahc, Srpk1, Stk24, Sugp2, Sympk, Syt6, Taf9b, Tagln3, Tamm41, Tanc2, Tbcel, Tmed5, Tnks1bp1, Tnr, Trabd, Trappc8, Trim3, Tsr2, Tsta3, U2surp, Ubap2, Ubxn4, Ufc1, Urgcp, Usp24, Uxt, Vangl1, Vezt, Wdr47, Wwc3, Wwp2, Zcchc12, Zer1, Zfp131, Zfp36l1, Zfp498, Zfp655

Supplemental Fig. 4

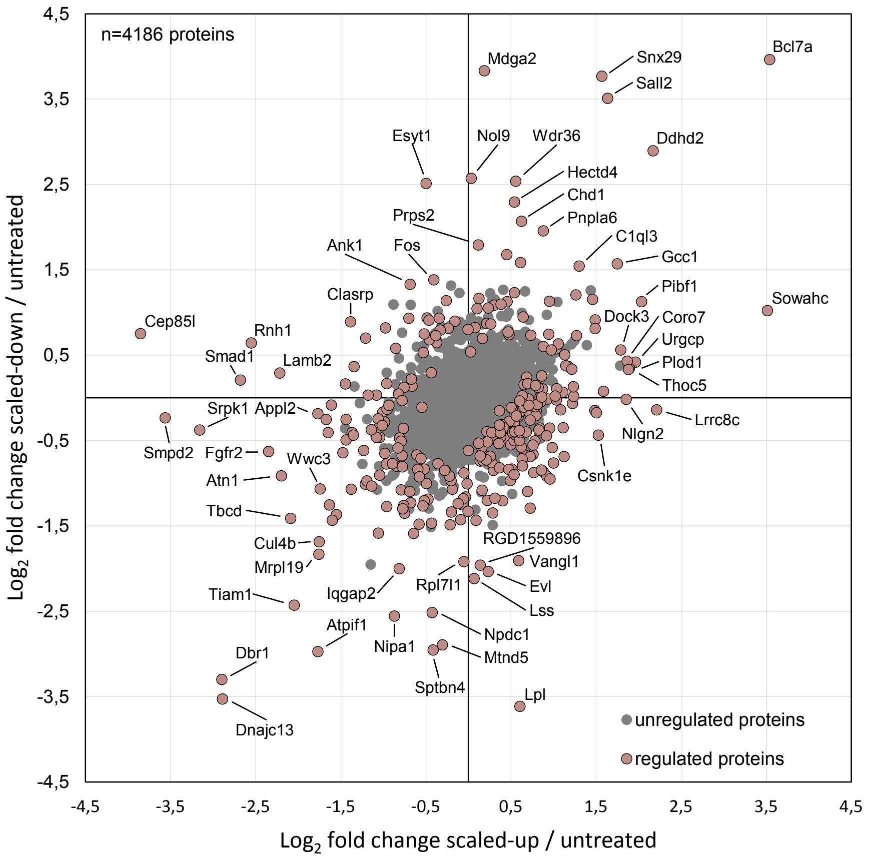

Supplemental Fig. 5

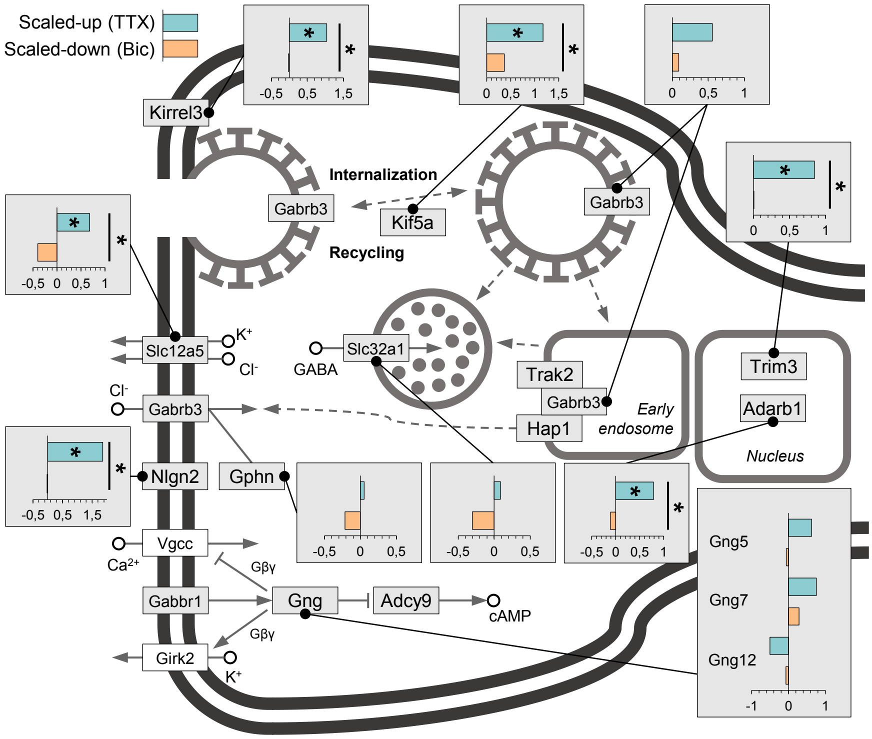

Schanzebaecher et al.,

Supplemental Figures.

Supplemental Figure 1, related to Figure 2. Protein synthesis is required for homeostatic up- and down-scaling.

(A) Scheme indicating experimental work-flow. The protein synthesis inhibitor anisomycin (40 μM) was applied for the first 12 hrs of scaling induction (24 hrs) by either TTX (1 μM) or bicuculline (40 μM).

(B) Representative electrophysiological recordings of miniature excitatory postsynaptic currents (mEPSCs) from control, TTX and TTX + anisomycin experiments. Analysis of mEPSC amplitude for groups, as indicated. TTX-treated neurons exhibited significantly larger mEPSCs than either control ($p < 0.0001$) or TTX + aniso-treated neurons ($p < 0.001$), which did not differ significantly from one another. There was no change in mEPSC frequency produced by TTX treatment (data not shown).

(C) Representative electrophysiological recordings of miniature excitatory postsynaptic currents (mEPSCs) from control, bicuculline and bicuculline + anisomycin experiments.Analysis of mEPSC amplitude for groups, as indicated. Bicuculline-treated neurons exhibited significantly smaller mEPSCs than either control ($p < 0.0066$) or Bicuculline + aniso-treated neurons ($p < 0.0023$), which did not differ significantly from one another. There was no change in mEPSC frequency produced by Bicuculline treatment (data not shown).

Supplemental Figure 2, related to Figures 2 and 3. Reproducibility of the dataset.

(A) Technical replicates for the global sample showing the average reproducibility of the 4 technical replicates that make up each of the 5 biological replicates.

(B) Biological replicate reproducibility of significantly regulated proteins in each of the quadrants of Figure 4A. Quadrants are as indicated and colored labels indicate the condition, scaling-up (teal), scaling-down (yellow) and whether the quadrant represents enhanced (“up”) or reduced (“down”) synthesis.

(C) Protein groups and numbers of proteins represented in the 4 quadrants of Figure 4A.

Supplemental Figure 3, related to Figure 4. Network analysis of all differentially regulated proteins. String pathway analysis showing that many (95) of the regulated proteins can be assembled into a single, large interactive network.

Supplemental Figure 4, related to Figure 4. Scatterplot of significantly regulated proteins showing quadrant localization with labels on the proteins that exhibit high levels of regulation.

Supplemental Figure 5, related to Figures 5 and 6. Proteins associated with inhibitory synaptic transmission regulated by homeostatic scaling. Scheme of an inhibitory synapse. Proteins in grey boxes were detected in our samples, call-out boxes are included for all proteins which fulfilled the criteria for the display of intensity values (see methods). The bars, teal and ochre, represent the regulation of the indicate protein in up- and down-scaling, respectively. Asterisks within the colored bar indicate that the protein exhibited significant (ANOVA FDR < 0.05 and Fisher LSD post-hoc $p < 0.05$) regulation relative to control; asterisks associated with the black lines indicate the protein exhibited significant (ANOVA FDR < 0.05 and Fisher LSD post-hoc $p < 0.05$) regulation between up- and down-scaling.

Supplemental Tables.

Supplemental Table 1 (separate file), related to Figure 2. Table of all proteins identified in each group.

Supplemental Table 2a, (separate file), related to Figure 3. Contains all 307 significantly regulated proteins.

Supplementary Table 2b, related to Figure 3. Low-abundance proteins with trends for enrichment conditions.

Supplemental Table 3, (separate file), related to Figure 3. Identified and significantly regulated proteins according to functional groups.

On different pages (tabs) we list the following protein groups:

a) Ion channels and ion transport

b) Cytoskeletal, cell adhesion and motor proteins

c) Synapse and cell junction

d) Kinases and phosphatases

e) Neurotransmitters and Exocytosis

f) Gene transcription

g) mRNA regulation, protein translation and protein degradation

Supplemental Table 4, (separate file), related to Figures 5-7. All proteins shown in Figures 5-7 with corresponding analysis.

Supplemental Table 5, (separate file), related to Figures 3 and 4. List of previously implicated homeostasis molecules and their detection and regulation in this study.

Supplemental Table 6, (separate file), related to Figures 5-7. Newly synthesized significantly regulated proteins of potential glial origin.

Supplemental Table 7, (separate file), related to Figure 8. Newly synthesized significantly regulated proteins associated with some neurodegenerative, neuropsychiatric and neurodevelopmental disorders.

Supplemental Table 8, related to Figure 3. Full list of protein groups (<50 proteins) found using 1D annotation enrichment (see Materials and Methods). Proteins are sorted by first column (mean log_2 fold change Bic/TTX), table also includes Median log2 fold change (Bic/TTX), group annotation name, group identifier, the number of proteins in the group, P value for 1D enrichment, Benjamini-Hochberg FDR and annotation database the group term derives from (GOCC = Gene Ontology Cellular Component, GOMF = Gene Ontology Molecular Function, GOBP = Gene Ontology Biological Processes, EMBL-EBI databases Pfam (Protein Families) and InterPro).

Supplemental Table 9, (separate file), related to Figure 1. Full list of LC-MS and MaxQuant parameters used in this study.

Supplemental Table S2b

Scaled up (TTX)

Protein ID(s)	Gene symbol(s)	Peptide ratio Bic/Untd	Peptide ratio TTX/Untd	Peptide ratio Bic/TTX	Log2 fold change Bic-Untd (±pooled standard error)	Log2 fold change TTX-Untd (±pooled standard error)	Log2 fold change Bic-TTX (±pooled standard error)	Bic: number of biological replicates	TTX: number of biological replicates	Untd: number of biological replicates	Bic: mean unique peptides	TTX: mean unique peptides	Untd: mean unique peptides	Bic: SD unique peptides	TTX: SD unique peptides	Untd: SD unique peptides	Bic: mean Log2 LFQ	TTX: mean Log2 LFQ	Untd: mean Log2 LFQ	Bic: coefficient of variation	TTX: coefficient of variation	Untd: coefficient of variation	Number of proteins	Mol. weight [kDa]	Sequence length	Score
P51400, G3V649	Adarb1	0.52	0.63	0.39	-0.106±0.252	0.786±0.204	-0.892±0.206	5	5	5	2.6	4.1	2.4	1.1	1.5	0.9	23.483	24.375	23.589	0.32	0.171	0.316	2	77.924	711	47.953
F1LV12, P26769	Adcy2	0.36	0.39	0.47	-	-	-	1	3	1	1	1.1	1.8	-	0.2	-	-	-	-	-	-	-	9	96.385	852	5.1394
P26817, F1LMA4	Adrbk1	0.42	0.64	0.29	-0.955±0.119	0.964±0.138	-1.919±0.175	5	5	5	1.2	3	1.7	0.4	1.1	0.8	21.923	23.842	22.878	0.193	0.229	0.058	3	79.784	689	6.9782
M0R7U1	Ak5	0.44	0.61	0.34	-0.848±0.369	0.861±0.455	-1.709±0.352	5	5	5	1.3	2.6	1.7	0.4	1.2	1.1	22.281	23.989	23.128	0.181	0.583	0.621	1	63.155	562	9.2167
F1MAK3, B5DEI5	Arhgap32, Rics	0.52	0.63	0.39	-0.237±0.494	0.699±0.463	-0.936±0.425	5	5	5	3.7	5.7	3.4	1.4	1	1.4	24.087	25.023	24.324	0.653	0.53	0.783	2	229.58	2086	56.028
B4F7F3	Arvcf	0.49	0.62	0.37	0.631±0.162	1.062±0.177	-0.43±0.16	4	5	4	1.5	2.6	1.6	0.4	1.4	0.4	21.801	22.231	21.169	0.146	0.203	0.196	1	105.49	973	22.025
Q64568-10, Q64568-	Atp2b3	0.5	0.5	0.5	-	-	-	1	3	1	1	1	1	-	0	-	-	22.662	-	-	0.265	-	6	126.82	1154	275.64
F1LXF1	Bcr	0.5	0.63	0.38	-	-	-	1	3	3	1	1.7	1	-	0.6	0	-	-	-	-	-	-	1	95.699	842	3.191
F1LSR8, Q8CFG5	Cacna2d3	0.47	0.6	0.38	-0.3±0.626	0.827±0.532	-1.127±0.51	2	4	3	1.1	1.9	1.3	0.2	1	0.4	21.558	22.685	21.858	0.35	0.55	0.72	2	121.94	1082	4.3118
F1LQD2, P97756	Camkk1	0.34	0.61	0.24	-	1.363±0.512	-	4	5	4	1.1	3.4	2.1	0.2	2	0.9	-	24.205	22.842	-	0.988	0.167	2	55.897	505	16.501
M0R7N7, M0R577, I	Caprin2	0.5	0.63	0.38	-	-	-	1	3	2	1	1.7	1	-	1.2	0	-	24.466	-	-	0.241	-	3	34.922	300	3.2209
D3ZG85, E2E1S0	Cdkl5	0.5	0.61	0.39	-0.361±0.572	0.43±0.674	-0.791±0.65	5	5	5	2.5	3.8	2.5	1.6	2.7	1.6	23.221	24.012	23.582	0.866	1.207	0.721	2	105.21	934	35.472
P97836, G3V849	Dlgap1	0.57	0.67	0.39	-0.757±0.566	0.316±0.421	-1.073±0.497	5	5	5	2.1	3.3	1.6	0.5	1.4	0.5	24.298	25.371	25.055	0.927	0.498	0.606	2	110.18	992	91.774
F1M3D2, M0RDT6	Dmwd	-	1	0	-	-	-	0	3	0	0	1.1	0	-	0.2	-	-	-	-	-	-	-	2	55.868	522	9.6649
F1M4N6	Dock3	0.48	0.64	0.34	0.557±0.639	1.793±0.432	-1.236±0.381	4	5	3	1	2	1.1	0	0.6	0.1	21.22	22.456	20.663	0.459	0.318	0.649	1	233.35	2030	22.554
G3V7D4, A5HTT2	Efnb3	0.48	0.6	0.38	0.038±0.597	0.854±0.554	-0.816±0.383	5	5	5	4.3	7.1	4.7	2.2	1.9	2.6	25.622	26.438	25.584	0.626	0.406	1.198	2	35.792	340	55.294
D3ZH36	Elfn2	0.46	0.62	0.35	-0.299±0.159	0.546±0.224	-0.845±0.148	5	4	4	1.6	3.1	1.9	0.4	1	0.5	23.125	23.969	23.423	0.093	0.233	0.258	1	89.846	820	38.94
F1MAJ0, B2B9B0	Ephb2	0.45	0.62	0.34	0.668±0.577	0.871±0.644	-0.203±0.639	5	5	5	1.5	3	1.9	0.5	2.3	1.2	23.828	24.031	23.16	0.082	1.097	0.952	2	107.47	963	31.288
D4A3K7	Frmpd4	0.47	0.61	0.36	-	-	-0.108±0.773	2	3	2	1.3	2.3	1.5	0.5	0.9	0.2	22.427	22.535	-	0.344	0.989	-	1	187.09	1705	18.053
G3V9C5, Q00959	Grin2a	0	0.42	0	-	-	-	0	4	1	0	1.3	1.8	-	0.4	-	-	22.826	-	-	0.279	-	7	165.48	1464	5.0079
E9PTD7, Q09GS6	Kirrel3	0.55	0.67	0.38	-0.026±0.244	1.037±0.288	-1.064±0.308	5	5	5	2	3.3	1.6	1	1.9	1.3	22.883	23.947	22.91	0.339	0.458	0.271	2	79.355	725	30.003
D3ZBH5	Lmtk2	0.5	0.55	0.45	-	-	-	1	3	1	1	1.2	1	-	0.4	-	-	21.9	-	-	0.144	-	1	165.33	1531	5.1098
D4A1J8, D4A4L4, W	Map7d2	0.47	0.61	0.37	-0.88±0.176	0.773±0.181	-1.653±0.171	5	5	5	1.5	2.5	1.6	0.4	1	0.8	23.012	24.664	23.892	0.201	0.212	0.197	3	66.185	594	17.317
D3ZN55	Ncor1	0.5	0.63	0.38	-	-	-	1	3	3	1	1.7	1	-	0.3	0	-	21.856	-	-	0.218	-	3	144.96	1336	2.3076
P70600-3, P70600	Ptk2b	0.4	0.64	0.27	-0.259±0.58	1.283±0.796	-1.542±0.77	5	5	5	2.1	5.6	3.2	1.7	3.5	2.4	23.02	24.562	23.279	0.815	1.814	0.96	3	111.09	967	49.921
Q9WV48-3, Q9WV4	Shank1	0.48	0.63	0.35	0.642±0.667	1.77±0.686	-1.128±0.623	4	5	4	2	3.7	2.2	2	3	2.1	23.152	24.281	22.511	0.525	0.903	1.016	6	225.47	2158	79.274
G3V8N8, Q8CIV0	Slc7a6os	0.52	0.63	0.39	-	-	-	5	3	3	1.1	1.7	1	0.2	0.9	0	-	22.923	-	-	0.305	-	2	35.032	305	19.073
F1LTE0	Tanc2	0.42	0.64	0.3	-0.587±0.499	0.989±0.467	-1.576±0.445	5	5	5	3.2	7.6	4.3	1.4	3	2.4	24.176	25.752	24.763	0.689	0.565	0.767	1	219.81	1992	36.474
D3ZTN1	Tesc	0.54	0.66	0.37	-0.44±0.353	0.834±0.375	-1.274±0.337	2	3	3	1.5	2.6	1.3	0.3	1.1	0.6	22.919	24.193	23.359	0.064	0.364	0.385	1	24.599	214	30.564
A0A096MK46, Q9R	Timm10b	0.5	0.6	0.39	-0.337±0.311	0.52±0.292	-0.856±0.302	5	4	5	1.1	1.6	1.1	0.1	0.3	0.1	23.146	24.002	23.483	0.331	0.249	0.35	3	11.657	100	6.4449
F1M0V0	Zfp280d	0.55	0.67	0.37	-	-	-	4	3	2	1.4	2.3	1.1	0.5	0.6	0.2	22.857	-	-	0.269	-	-	2	107.28	969	23.902

Scaled down (Bic)

Protein ID(s)	Gene symbol(s)	Peptide ratio Bic/Untd	Peptide ratio TTX/Untd	Peptide ratio Bic/TTX	Log2 fold change Bic-Untd (±pooled standard error)	Log2 fold change TTX-Untd (±pooled standard error)	Log2 fold change Bic-TTX (±pooled standard error)	Bic: number of biological replicates	TTX: number of biological replicates	Untd: number of biological replicates	Bic: mean unique peptides	TTX: mean unique peptides	Untd: mean unique peptides	Bic: SD unique peptides	TTX: SD unique peptides	Untd: SD unique peptides	Bic: mean Log2 LFQ	TTX: mean Log2 LFQ	Untd: mean Log2 LFQ	Bic: coefficient of variation	TTX: coefficient of variation	Untd: coefficient of variation	Number of proteins	Mol. weight [kDa]	Sequence length	Score
F1M400	Ankrd28	1	-	1	-	-	-	3	0	0	1	0	0	0	-	-	-	-	-	-	-	-	1	112.96	1053	3.3067
D4A9G6	Arhgap33	0.56	0.5	0.56	-	-	-	4	1	1	1.3	1	1	0.5	-	-	22.994	-	-	0.204	-	-	1	137.39	1284	3.1981
O08560	Dgkz	0.57	0.6	0.47	-	-	-	3	1	1	1.3	1.5	1	0.6	-	-	22.667	-	-	0.199	-	-	1	104.01	929	2.0479
P12841, Q63752, Q5	Fos, c-fos	0.63	0.45	0.67	1.378±0.365	-0.401±0.319	1.779±0.348	5	5	5	2.6	1.2	1.5	1.6	0.3	0.6	24.387	22.608	23.009	0.535	0.389	0.447	4	40.926	380	109.69
Q9EQH1, F1LSR2	Gab2	0.6	0.5	0.6	0.288±0.287	0.201±0.28	0.087±0.302	4	4	5	1.5	1	1	0.4	0	0	21.722	21.634	21.434	0.31	0.132	0.277	2	73.327	665	3.4289
D3ZKF5, A0A0A0M	Htra4	0.68	0.46	0.71	1.628±1.264	-0.316±0.667	1.944±0.996	5	4	3	5.1	2.1	2.4	3.3	0.9	1.8	24.947	23.003	23.319	2.791	0.377	1.395	2	52.192	488	151.84
D3ZVG3, P70581-4,	Nupl1	0.63	0.52	0.62	0.179±0.228	-0.083±0.184	0.262±0.22	5	5	5	2.3	1.4	1.3	1.7	0.3	0.2	23.558	23.297	23.38	0.302	0.183	0.211	5	51.317	507	19.44
D3ZK25	RGD1309748	1	-	1	-	-	-	3	0	0	1.1	0	0	0.1	-	-	-	-	-	-	-	-	2	30.939	275	1.6071
Q6AZ64, Q8VIB1, E	Xrcc6	0.63	0.5	0.63	0.281±0.255	0.04±0.395	0.241±0.347	4	4	5	1.7	1	1	1.2	0	0	24.01	23.769	23.728	0.157	0.477	0.31	4	69.486	608	5.286

Scaled down (Bic) + Scaled up (TTX)

Protein ID(s)	Gene symbol(s)	Peptide ratio Bic/Untd	Peptide ratio TTX/Untd	Peptide ratio Bic/TTX	Log2 fold change Bic-Untd (±pooled standard error)	Log2 fold change TTX-Untd (±pooled standard error)	Log2 fold change Bic-TTX (±pooled standard error)	Bic: number of biological replicates	TTX: number of biological replicates	Untd: number of biological replicates	Bic: mean unique peptides	TTX: mean unique peptides	Untd: mean unique peptides	Bic: SD unique peptides	TTX: SD unique peptides	Untd: SD unique peptides	Bic: mean Log2 LFQ	TTX: mean Log2 LFQ	Untd: mean Log2 LFQ	Bic: coefficient of variation	TTX: coefficient of variation	Untd: coefficient of variation	Number of proteins	Mol. weight [kDa]	Sequence length	Score
O88881-2, O88881	Begain	0.63	0.6	0.53	0.254±0.282	-0.046±0.313	0.301±0.326	4	4	4	2.5	2.3	1.5	1.8	1.8	1	24.744	24.443	24.49	0.299	0.338	0.024	2	66.161	605	46.057
Q9ET45	Bnip3	0.6	0.63	0.47	0.489±0.549	0.595±0.467	-0.106±0.607	5	5	4	1.6	1.8	1.1	0.6	1.1	0.1	22.887	22.993	22.398	0.993	0.777	0.379	2	20.978	187	9.5344
D4A3T5	C1ql3	0.64	0.66	0.48	1.54±0.439	1.306±0.316	0.233±0.402	4	3	3	2.4	2.6	1.3	1.2	0.9	0.6	24.698	24.464	23.158	0.5	0.34	0.039	1	26.687	255	62.802
P13109, A0A2U1	Fgf2	0.5	0.5	0.5	-	-	-	3	4	1	1	1	1	0	0	-	-	-	-	-	-	-	2	17.138	154	1.8796
Q80YG1, E9PSV5, Q	Psat1	0.65	0.6	0.55	0.49±1.019	0.572±0.199	-0.082±0.91	5	5	5	3.2	2.6	1.7	3.2	1.1	0.5	23.287	23.369	22.797	3.008	0.284	0.132	3	40.588	370	23.396
B1VKB4, Q9Z327-3,	Synpo	0.6	0.65	0.45	1.218±0.686	1.146±0.734	0.073±0.651	5	5	5	4.6	5.7	3.1	3.3	3.3	2.2	25.475	25.402	24.256	0.648	1.176	1.296	4	96.717	905	109.71

Untd

Protein ID(s)	Gene symbol(s)	Peptide ratio Bic/Untd	Peptide ratio TTX/Untd	Peptide ratio Bic/TTX	Log2 fold change Bic-Untd (±pooled standard error)	Log2 fold change TTX-Untd (±pooled standard error)	Log2 fold change Bic-TTX (±pooled standard error)	Bic: number of biological replicates	TTX: number of biological replicates	Untd: number of biological replicates	Bic: mean unique peptides	TTX: mean unique peptides	Untd: mean unique peptides	Bic: SD unique peptides	TTX: SD unique peptides	Untd: SD unique peptides	Bic: mean Log2 LFQ	TTX: mean Log2 LFQ	Untd: mean Log2 LFQ	Bic: coefficient of variation	TTX: coefficient of variation	Untd: coefficient of variation	Number of proteins	Mol. weight [kDa]	Sequence length	Score
Q5FVP8	Dgat2	0	0	-	-	-	-	0	0	3	0	0	1.2	-	-	0.4	-	-	-	-	-	-	1	43.794	388	4.6894
Q4V890	Fem1a	0.35	0.37	0.48	-0.772±0.228	-0.887±0.188	0.115±0.191	5	5	4	1.1	1.1	1.9	0.1	0.3	1.1	21.077	20.962	21.85	0.253	0.147	0.247	1	72.117	654	18.05
B2GV98	Lrch1	0.36	0.37	0.5	-	-	-	5	3	4	1.1	1.1	1.9	0.1	0.1	0.6	-	-	23.534	-	-	0.158	1	75.211	677	6.3225
F1LPM4, P19643	Maob	0.39	0.36	0.54	-0.547±0.5	-	-	4	3	5	1.3	1.1	2	0.4	0.1	1.5	23.877	-	24.424	0.313	-	0.545	3	56.94	504	18.22
F1M587, F1M1F1	March6	0.38	0.39	0.49	-0.908±0.332	-1.04±0.181	0.132±0.283	5	5	4	1.1	1.1	1.8	0.1	0.2	1.2	22.165	22.033	23.073	0.501	0.174	0.243	2	99.256	883	8.5965
F1M5N4	Me3	0.35	0.4	0.44	-	-	-	2	3	3	1	1.3	1.9	0	0.3	0.8	-	-	23.143	-	-	0.117	1	67.208	605	20.174
C6JUM5	Pcdhgc5	0.4	0.37	0.53	-	-0.661±0.431	-	5	5	5	1.9	1.7	2.8	1	0.8	2	-	22.887	23.548	-	0.477	0.481	1	102.1	944	112.73
F1LQM9	Xpo7	0.39	0.36	0.53	-0.374±0.207	-0.622±0.188	0.248±0.19	5	4	3	1.2	1.1	1.9	0.3	0.1	0.2	21.032	20.784	21.407	0.194	0.005	0.191	2	124.18	1090	15.854

Scaled up (TTX) + Untd

Protein ID(s)	Gene symbol(s)	Peptide ratio Bic/Untd	Peptide ratio TTX/Untd	Peptide ratio Bic/TTX	Log2 fold change Bic-Untd (±pooled standard error)	Log2 fold change TTX-Untd (±pooled standard error)	Log2 fold change Bic-TTX (±pooled standard error)	Bic: number of biological replicates	TTX: number of biological replicates	Untd: number of biological replicates	Bic: mean unique peptides	TTX: mean unique peptides	Untd: mean unique peptides	Bic: SD unique peptides	TTX: SD unique peptides	Untd: SD unique peptides	Bic: mean Log2 LFQ	TTX: mean Log2 LFQ	Untd: mean Log2 LFQ	Bic: coefficient of variation	TTX: coefficient of variation	Untd: coefficient of variation	Number of proteins	Mol. weight [kDa]	Sequence length	Score
Q8CF82, M0RA83, C	Abca5	0	0.5	0	-	0.627±0.231	-	0	3	3	0	1.3	1.3	-	0.6	0.6	-	21.852	21.225	-	0.238	0.036	3	185.81	1642	7.6809
D3ZRJ0	Adck1	0	0.5	0	-	-0.153±0.284	-	0	4	3	0	1	1	-	0	0	-	20.29	20.444	-	0.24	0.194	1	51.71	455	1.5998
Q5BJQ0	Adck3	0.38	0.58	0.31	-0.562±0.363	0.448±0.406	-1.01±0.516	5	5	4	1.1	2.4	1.8	0.2	1.1	0.4	22.525	23.535	23.087	0.109	0.608	0.391	1	72.225	649	19.662
A0A096MJB0	Adcy9	0.38	0.54	0.34	-0.916±0.401	0.139±0.45	-1.055±0.467	4	4	3	1.2	2.3	1.9	0.2	1.3	0.6	22.255	23.31	23.171	0.53	0.63	0.275	2	151	1354	16.431
Q8CGU4	Agap2	0.33	0.54	0.29	-0.193±0.553	0.301±0.524	-0.493±0.593	2	4	3	1.2	2.9	2.4	0.2	2.5	2.5	23.085	23.578	23.278	0.473	0.65	0.546	1	124.44	1186	27.334
F1M446	AI314180	0.4	0.53	0.38	-0.709±0.126	0.227±0.101	-0.936±0.122	5	5	5	3.9	6.5	5.9	0.6	1.3	2.7	22.847	23.784	23.556	0.169	0.11	0.124	2	203.66	1838	39.529
Q9WUS0	Ak4	0.4	0.52	0.38	-0.59±0.475	0.592±0.443	-1.181±0.274	5	5	5	2	3.3	3.1	1.3	1.1	1.5	24.214	25.396	24.804	0.425	0.265	0.905	1	25.203	223	64.079
D4A205	Apc2	0.35	0.47	0.38	-	-0.09±0.885	-	2	5	3	1.2	1.9	2.2	0.2	1.6	2	-	23.396	23.486	-	0.982	1.47	1	243.93	2281	14.403
B2DD29	Brsk1	0.4	0.54	0.36	-0.866±0.248	0.886±0.172	-1.753±0.208	4	4	3	1.7	3	2.6	0.7	0.9	1.1	21.723	23.475	22.589	0.272	0.154	0.19	1	85.182	778	80.707
Q9ERS3, F7F134, D3	Cacna2d1	0.35	0.54	0.31	-0.59±0.523	-0.004±0.857	-0.586±0.739	5	5	4	2	4.4	3.6	1.4	3	3.2	23.701	24.287	24.29	0.19	1.717	1.022	6	123.47	1091	77.115
D3ZLT0, P55215-2,	Casp2	0	0.5	0	-	-	-	0	3	3	0	1	1	-	0	0	-	-	-	-	-	-	3	38.043	343	1.5126
G3V8P3, Q9QYP2	Celsr2	0.4	0.53	0.37	-0.719±0.272	0.178±0.308	-0.897±0.205	5	5	5	2.5	4.2	3.7	1.6	2.7	2.1	23.282	24.179	24.001	0.172	0.317	0.478	2	317.12	2919	78.803
Q9QX19, E9PSL7	Cit	0.36	0.54	0.32	-0.637±0.618	0.41±0.62	-1.047±0.334	4	4	3	2.8	5.9	5	1.5	2.4	3.7	23.579	24.626	24.215	0.383	0.391	1.21	2	185.92	1618	67.634
H1UBM7, F1M1L9	Cpne2	0.4	0.52	0.38	-0.145±0.303	0.479±0.215	-0.624±0.184	4	4	4	1.1	1.9	1.7	0.3	0.2	0.6	22.821	23.444	22.966	0.286	0.07	0.336	2	61.121	548	18.708
F1LN46	Cpt1c	0.31	0.44	0.37	-1.135±0.844	0.106±0.634	-1.24±0.409	4	5	4	1.3	2.2	2.8	0.4	1.4	2.2	21.579	22.819	22.714	0.556	0.431	1.487	2	90.169	801	64.585
Q45NC2, E9PT92	Csmd1	0.36	0.47	0.38	-	0.217±0.235	-	4	5	3	1.1	1.8	2	0.3	0.9	0.6	-	22.366	22.149	-	0.228	0.289	4	387.61	3564	14.824
F1LQE1, Q6P730	Dab2ip	0.39	0.54	0.35	-0.241±0.211	0.04±0.152	-0.281±0.158	5	5	5	1.1	2.1	1.8	0.1	0.6	0.3	23.429	23.71	23.67	0.266	0.069	0.254	2	126.76	1148	36.718
D3ZT20	Fbxo41	0.39	0.53	0.36	-1.101±0.564	0.7±0.469	-1.801±0.317	4	4	3	1.1	1.9	1.7	0.1	0.9	1.2	20.887	22.688	21.988	0.015	0.34	0.738	1	93.854	870	20.498
B1H234	Flrt3	0.31	0.51	0.3	-1.435±0.17	0.094±0.134	-1.529±0.192	5	4	4	1.2	2.6	2.6	0.2	1.3	0.8	22.705	24.235	24.141	0.239	0.171	0.104	2	72.91	649	5.8944
F1LPQ7, Q5U4E6	Golga4	0.29	0.41	0.37	-1.536±0.729	-0.806±0.458	-0.73±0.644	5	5	5	1.3	2.2	3.2	0.3	0.8	2.2	22.185	22.916	23.721	1.318	0.347	0.753	3	260.27	2259	56.563
G3V6A8, Q63714	Golgb1	0.24	0.46	0.27	-1.66±1.043	0.171±1.149	-1.83±0.792	5	5	5	1.8	4.9	5.7	0.6	6.1	6.5	22.053	23.884	23.713	0.15	1.931	3.138	2	364.25	3187	165.31
D3ZAP9	Gpd1l	0.35	0.52	0.32	-0.484±0.292	0.465±0.23	-0.949±0.253	4	4	4	2.3	4.7	4.3	0.7	0.8	0.4	25.245	26.194	25.729	0.355	0.191	0.306	1	38.066	351	69.156
A0A0A0MY13, D4A	Gpr158	0.24	0.43	0.3	-1.47±0.772	-0.29±0.915	-1.18±0.457	4	5	3	1.3	3.2	4.1	0.5	2.2	3.8	23.204	24.384	24.674	0.206	0.831	1.956	2	134.83	1206	76.474
G3V746, Q00960	Grin2b	0.33	0.5	0.33	-0.989±0.582	-0.509±0.572	-0.48±0.468	4	5	3	1.3	2.7	2.7	0.3	1.9	2.3	22.655	23.135	23.644	0.366	0.645	0.868	2	166.03	1482	55.339

D4A0C3	Hid1	0.39	0.52	0.38	-0.974±0.341	0.497±0.279	-1.471±0.277	5	5	5	1.2	2	1.9	0.3	0.5	0.7	21.188	22.659	22.162	0.089	0.285	0.368	1	88.753	788	10.696
G3V9C1, Q99MG9-2	Kcnip4	0.39	0.52	0.37	-	0.188±0.302	-	2	5	3	1	1.7	1.6	0	0.7	0.1	-	22.82	22.632	-	0.419	0.052	3	24.589	213	6.5055
F1M5N7	Kif21b	0.37	0.56	0.31	-0.691±0.33	0.731±0.388	-1.422±0.231	5	5	5	2.1	4.6	3.6	0.6	1.4	2.3	23.214	24.636	23.905	0.127	0.402	0.646	1	182.56	1634	120.12
M0RC60, D3ZNV6	LOC100910164, Elm	0.39	0.51	0.38	-0.576±0.107	-0.134±0.154	-0.442±0.176	5	5	5	1.5	2.4	2.3	0.3	1.1	0.6	23.404	23.846	23.98	0.162	0.256	0.072	2	34.792	293	7.9191
P21708, P21708-2	Mapk3	0.38	0.54	0.34	-1.177±0.151	0.315±0.152	-1.492±0.177	5	5	5	2.3	4.5	3.8	1	0.4	0.6	23.506	24.999	24.684	0.212	0.216	0.142	4	43.08	380	10.306
Q6AY81	Npdc1	0.4	0.53	0.37	-2.517±0.637	-0.422±0.404	-2.096±0.261	3	5	3	1	1.7	1.5	0	0.5	0.5	21.61	23.706	24.127	0.193	0.252	0.82	1	35.516	331	7.9218
D3ZQD3	Ogdhl	0.39	0.51	0.38	-0.627±0.44	0.589±0.474	-1.217±0.284	4	5	4	1.6	2.7	2.6	0.7	1.9	1.8	21.929	23.146	22.556	0.089	0.465	0.83	1	116.71	1029	22.886
P53817	Pla2g16	0.36	0.48	0.38	-0.579±0.371	-0.405±0.263	-0.175±0.356	5	5	5	1	1.6	1.8	0	0.3	0.4	23.728	23.903	24.308	0.623	0.302	0.365	1	17.748	160	8.1993
M0RDL0, A0A096M	Prdm16	0	0.5	0	-	-	-	0	5	5	0	1	1	-	0	0	-	-	-	-	-	-	4	110.18	992	1.5268
P63319	Prkcg	0.31	0.44	0.37	-	0.478±0.917	-	2	4	3	1.6	2.8	3.6	0.2	2.3	2.8	-	24.472	23.994	-	0.634	2.222	1	78.357	697	31.548
D4A9C3, D4A404, D	Psd3	0.34	0.52	0.32	-0.975±0.572	0.4±0.569	-1.375±0.504	4	4	4	3.1	6.5	5.9	1.3	3.8	3.5	24.799	26.173	25.774	0.643	0.633	0.855	7	42.285	376	33.942
F1M6Y5, G3V953	Rbbp6	0.36	0.5	0.36	-0.511±0.492	0.102±0.555	-0.613±0.288	5	5	5	1.4	2.5	2.5	0.4	0.8	1.6	23.094	23.707	23.605	0.153	0.526	1.115	3	195.57	1755	152.27
F1M7Z9, Q8CIX1	Rims4	0.37	0.53	0.35	-0.61±0.426	0.663±0.317	-1.273±0.23	3	4	4	1.1	2	1.8	0.1	0.3	0.9	21.617	22.89	22.227	0.141	0.219	0.47	2	29.329	269	33.336
Q6RJR6	Rtn3	0.37	0.5	0.37	-1.109±0.509	0.154±0.553	-1.262±0.746	3	5	4	1.7	2.8	2.8	0.8	2.5	1.9	22.414	23.676	23.522	0.46	0.944	0.517	1	101.52	940	60.186
B2GUY8	Slc25a17	0.4	0.52	0.38	-1.207±0.226	0.408±0.275	-1.615±0.275	5	5	5	1.1	1.8	1.6	0.1	0.4	0.5	21.693	23.308	22.9	0.281	0.415	0.28	1	34.34	307	3.5625
F1LSL8	Sptbn4	0.37	0.61	0.27	-2.957±0.474	-0.408±0.454	-2.549±0.319	5	5	5	2.9	7.8	5	1.5	3.5	3.4	23.338	25.886	26.295	0.462	0.371	0.877	2	288.68	2561	126.18
Q99P36, Q99P34, Q9	SytVII, Syt7	0.39	0.54	0.35	-1.494±0.681	0.29±0.617	-1.784±0.744	4	5	4	1.3	2.3	2	0.5	1.2	1.4	21.497	23.281	22.991	0.418	0.948	0.815	8	58.161	523	26.873
Q3ZBA0	Tecpr1	0.34	0.45	0.38	-	-0.52±0.454	-	3	5	4	1.1	1.8	2.2	0.2	1.1	1.4	-	22.377	22.897	-	0.522	0.597	1	130.19	1166	27.439
Q9R1K2-4, Q9R1K2	Tenm2	0.31	0.5	0.3	-0.035±0.478	0.595±0.526	-0.631±0.376	5	5	5	1.9	4.3	4.2	1.2	3.4	3.4	24.197	24.828	24.232	0.316	0.556	0.837	6	299.93	2703	75.038
F1LZ38	Tenm4	0.32	0.53	0.3	-	0.608±0.458	-	2	4	4	1	2.4	2.1	0	1.5	1.7	-	24.219	23.612	-	0.269	0.462	1	310.87	2794	53.361
F1LQ63, A0A096MJ	Tnr	0.37	0.48	0.38	-1.472±0.317	-0.428±0.218	-1.044±0.233	4	5	5	1.5	2.4	2.6	0.5	0.5	1.3	22.474	23.518	23.946	0.408	0.125	0.372	4	139.43	1266	146.03
D3ZF39	Uap1	0.38	0.49	0.4	-0.901±0.326	-0.289±0.306	-0.612±0.392	5	5	5	1.7	2.6	2.8	0.5	0.6	0.6	23.127	23.739	24.028	0.563	0.509	0.266	1	58.394	521	11.287

Scaled down (Bic) + Untd

Protein ID(s)	Gene symbol(s)	Peptide ratio Bic/Untd	Peptide ratio TTX/Untd	Peptide ratio Bic/TTX	Log2 fold change Bic-Untd (±pooled standard error)	Log2 fold change TTX-Untd (±pooled standard error)	Log2 fold change Bic-TTX (±pooled standard error)	Bic: number of biological replicates	TTX: number of biological replicates	Untd: number of biological replicates	Bic: mean unique peptides	TTX: mean unique peptides	Untd: mean unique peptides	Bic: SD unique peptides	TTX: SD unique peptides	Untd: SD unique peptides	Bic: mean Log2 LFQ	TTX: mean Log2 LFQ	Untd: mean Log2 LFQ	Bic: coefficient of variation	TTX: coefficient of variation	Untd: coefficient of variation	Number of proteins	Mol. weight [kDa]	Sequence length	Score
P06238, M0R9G2, Q	A2m, LOC10091154	0.51	0.39	0.61	0.456±0.641	-0.449±0.599	0.904±0.382	4	4	4	2.6	1.6	2.6	1.3	0.6	1.2	23.468	22.563	23.012	0.267	0.497	1.066	6	163.78	1472	17.943
Q9JK64, Q6PSM0, Q	Abcb1a, Mdr1a, Abcb	0.46	0.34	0.62	-0.44±0.359	-1.348±0.254	0.908±0.404	5	5	5	2	1.2	2.3	0.7	0.4	1.6	23.205	22.297	23.645	0.687	0.413	0.209	13	140.33	1272	36.294
P47853	Bgn	0.49	0.36	0.63	-0.25±0.662	-1.756±0.465	1.506±0.444	4	5	5	1.9	1.1	2	1.4	0.2	1.7	22.932	21.426	23.182	0.949	0.206	1.006	1	41.706	369	23.263
Q5U2R3	Frmd8	0.48	0.37	0.61	-0.298±0.253	-0.71±0.28	0.412±0.249	5	5	5	3.2	2	3.5	0.9	1.1	0.9	24.543	24.13	24.84	0.271	0.354	0.363	1	51.781	466	155.69
F1MAB8	Kif11	0.51	0.4	0.61	0.697±0.359	-1.203±0.379	1.9±0.316	5	3	4	1.9	1.2	1.8	1.2	0.3	0.7	22.916	21.016	22.219	0.329	0.144	0.413	2	118.29	1056	61.704
M0R6K0, P15800, Q	Lamb2	0.48	0.33	0.65	0.289±0.445	-2.214±0.458	2.503±0.479	5	5	5	4.6	2.4	5	1.4	1.1	1.7	25.394	22.891	25.105	0.668	0.713	0.589	3	196.53	1801	161.31
D3ZPN5	Mtpap	0.47	0	1	-0.032±0.519	-	-	4	0	3	1	0	1.1	0	-	0.2	21.998	-	22.03	0.361	-	0.691	1	37.284	336	8.2877
D4AAE6	Rab20	0.55	0.39	0.65	0.358±0.175	-	-	5	4	4	2.1	1.1	1.7	0.7	0.2	0.6	23.819	-	23.46	0.018	-	0.175	1	25.805	232	28.19
M0R8K1, D3Z8N2	Rnf187	0.5	0	1	0.739±0.618	-	-	3	0	3	1	0	1	0	-	0	22.386	-	21.647	0.794	-	0.581	2	26.295	236	2.3758
P29524	Serpinb2	0.54	0.35	0.68	0.782±0.636	-1.188±0.234	1.97±0.623	4	4	4	5.1	2.4	4.4	3.4	0.9	1.5	26.028	24.059	25.247	1.321	0.214	0.297	1	47.247	416	36.081
Q5D006, Q6IE71, F7	Usp11	0.5	0.37	0.62	-0.401±0.255	-0.835±0.242	0.434±0.189	5	5	5	1.7	1.1	1.8	0.4	0.1	0.4	22.342	21.908	22.742	0.252	0.207	0.383	4	105.24	921	12.154

Supplemental Table S8.

Mean log_2 fold change Bic/TTX	Median log_2 fold change Bic/TTX	Annotation name	Identifier	Annotation size	P value	Benj. Hoch. FDR	Annotation database
-1.01	-0.94	Ionotropic glutamate receptor complex	GO:0008328	4	1.20E-03	0.0456	Gene ontology (cellular component)
-0.682	-0.522	CAMK Ser/Thr protein kinase family	-	13	3.90E-05	0.0285	Protein families
-0.664	-0.566	Voltage-gated calcium channel complex	GO:0005891	5	1.60E-03	0.0145	GOCC slim
-0.608	-0.532	Dendritic shaft	GO:0043198	21	1.10E-05	0.0010	Gene ontology (cellular component)
-0.608	-0.532	Dendritic shaft	GO:0043198	21	1.10E-05	0.0074	Gene ontology (GO)
-0.553	-0.519	Laminin G domain	PF02210	9	6.90E-05	0.0273	Cross-reference (Pfam)
-0.513	-0.537	Calcium ion transport	GO:0006816	14	9.10E-05	0.0283	Gene ontology (GO)
-0.498	-0.513	Rho guanyl-nucleotide exchange factor activity	GO:0005089	10	1.50E-04	0.0391	Gene ontology (GO)
-0.498	-0.513	Rho guanyl-nucleotide exchange factor activity	GO:0005089	10	1.50E-04	0.0288	Gene ontology (molecular function)
-0.489	-0.378	AMPA selective glutamate receptor complex	GO:0032281	10	1.30E-03	0.0471	Gene ontology (cellular component)
-0.483	-0.355	Inflammatory response	-	8	2.80E-03	0.0353	Uniprot keywords
-0.47	-0.455	Dbl homology (DH) domain	IPR000219	12	5.80E-05	0.0195	Cross-reference (InterPro)
-0.47	-0.455	Dbl homology (DH) domain	PF00621	12	5.80E-05	0.0343	Cross-reference (Pfam)
-0.468	-0.351	Peptidyl-serine phosphorylation	GO:0018105	27	1.60E-05	0.0085	Gene ontology (GO)
-0.461	-0.338	Neurotransmitter transport	-	10	1.70E-03	0.0234	Uniprot keywords
-0.445	-0.474	Ligand-gated ion channel	-	12	1.90E-03	0.0264	Uniprot keywords
-0.44	-0.363	Guanine-nucleotide releasing factor	-	15	3.30E-05	0.0009	Uniprot keywords
-0.434	-0.376	Exocytosis	-	24	3.40E-05	0.0009	Uniprot keywords
-0.408	-0.415	Presynaptic membrane	GO:0042734	21	3.70E-04	0.0178	Gene ontology (cellular component)
-0.4	-0.369	Voltage-gated channel	-	15	2.40E-03	0.0309	Uniprot keywords
-0.394	-0.438	Postsynaptic cell membrane	-	42	6.70E-07	0.0000	Uniprot keywords
-0.391	-0.388	Protein-tyrosine phosphatase-like	IPR029021	31	1.70E-05	0.0074	Cross-reference (InterPro)
-0.373	-0.318	Neurotransmitter transport	GO:0006836	23	1.10E-03	0.0136	GOBP slim
-0.367	-0.319	Neuron migration	GO:0001764	35	1.80E-04	0.0445	Gene ontology (GO)
-0.365	-0.301	Exocytosis	GO:0006887	46	2.30E-06	0.0001	GOBP slim
-0.365	-0.365	Ion channel	-	39	2.60E-04	0.0051	Uniprot keywords
-0.362	-0.324	Exocytosis	GO:0006887	40	2.20E-05	0.0405	Gene ontology (biological process)
-0.362	-0.324	Exocytosis	GO:0006887	40	2.20E-05	0.0095	Gene ontology (GO)
-0.337	-0.26	Terminal bouton	GO:0043195	45	1.70E-05	0.0014	Gene ontology (cellular component)
-0.337	-0.26	Terminal bouton	GO:0043195	45	1.70E-05	0.0087	Gene ontology (GO)
-0.325	-0.351	Calmodulin-binding	-	24	1.50E-03	0.0220	Uniprot keywords
-0.322	-0.252	Protein kinase superfamily	-	96	3.30E-07	0.0005	Protein families
-0.302	-0.277	Perikaryon	GO:0043204	32	5.90E-04	0.0269	Gene ontology (cellular component)
-0.3	-0.335	C2 domain	PF00168	38	1.10E-05	0.0136	Cross-reference (Pfam)
-0.292	-0.25	Actin cytoskeleton organization	GO:0030036	41	1.90E-04	0.0445	Gene ontology (GO)
-0.29	-0.304	GTPase activator activity	GO:0005096	46	1.00E-04	0.0289	Gene ontology (GO)
-0.29	-0.304	GTPase activator activity	GO:0005096	46	1.00E-04	0.0247	Gene ontology (molecular function)
-0.286	-0.31	GTPase activation	-	20	3.00E-03	0.0366	Uniprot keywords
-0.239	-0.296	Site of polarized growth	GO:0030427	49	7.30E-05	0.0015	GOCC slim
0.044	0.195	Extracellular matrix	GO:0031012	28	1.50E-03	0.0145	GOCC slim
0.084	0.13	Ribosome	GO:0005840	39	5.30E-03	0.0342	GOCC slim
0.126	0.089	Translation	GO:0006412	44	2.00E-03	0.0226	GOBP slim
0.153	0.126	RNA polymerase II core promoter proximal region sequence-specific DNA b	GO:0000978	44	5.40E-05	0.0183	Gene ontology (GO)
0.153	0.126	RNA polymerase II core promoter proximal region sequence-specific DNA b	GO:0000978	44	5.40E-05	0.0157	Gene ontology (molecular function)
0.158	0.223	Proteinaceous extracellular matrix	GO:0005578	16	4.70E-03	0.0361	GOCC slim
0.178	0.113	SRP-dependent cotranslational protein targeting to membrane	REACT_345353	33	9.10E-05	0.0116	Cross-reference (Reactome)
0.211	0.151	GTP hydrolysis and joining of the 60S ribosomal subunit	REACT_338061	33	3.00E-05	0.0090	Cross-reference (Reactome)
0.215	0.151	L13a-mediated translational silencing of Ceruloplasmin expression	REACT_286215	31	5.60E-05	0.0084	Cross-reference (Reactome)
0.246	0.133	Zinc finger C2H2-type/integrase DNA-binding domain	IPR013087	47	8.60E-07	0.0007	Cross-reference (InterPro)
0.252	0.135	Zinc finger, C2H2	PF00096	28	6.50E-05	0.0308	Cross-reference (Pfam)
0.257	0.233	Formation of a pool of free 40S subunits	REACT_286803	23	3.20E-05	0.0072	Cross-reference (Reactome)
0.265	0.281	RNA polymerase II core promoter proximal region sequence-specific DNA b	GO:0001077	20	2.00E-04	0.0453	Gene ontology (GO)
0.265	0.281	RNA polymerase II core promoter proximal region sequence-specific DNA b	GO:0001077	20	2.00E-04	0.0308	Gene ontology (molecular function)
0.284	0.289	Double-stranded DNA binding	GO:0003690	30	1.10E-05	0.0069	Gene ontology (GO)
0.284	0.289	Double-stranded DNA binding	GO:0003690	30	1.10E-05	0.0064	Gene ontology (molecular function)
0.301	0.26	Nonsense Mediated Decay (NMD) independent of the Exon Junction Comple	REACT_285246	16	2.20E-04	0.0243	Cross-reference (Reactome)
0.305	0.287	Nonsense Mediated Decay (NMD) enhanced by the Exon Junction Complex	REACT_320469	25	9.10E-06	0.0081	Cross-reference (Reactome)
0.329	0.283	Citrullination	-	14	3.90E-04	0.0069	Uniprot keywords
0.365	0.282	Response to cAMP	GO:0051591	20	1.50E-04	0.0402	Gene ontology (GO)
0.369	0.209	Proteasome core complex	GO:0005839	9	8.30E-04	0.0344	Gene ontology (cellular component)
0.369	0.209	Threonine protease	-	9	8.30E-04	0.0132	Uniprot keywords
0.385	0.3	Eukaryotic Translation Termination	REACT_310475	13	3.60E-05	0.0065	Cross-reference (Reactome)
0.42	0.36	Peptide chain elongation	REACT_299036	12	1.80E-05	0.0081	Cross-reference (Reactome)
0.492	0.4	Basic-leucine zipper domain	IPR004827	17	5.10E-05	0.0184	Cross-reference (InterPro)
0.652	0.699	Basic-leucine zipper domain	PF00170	11	2.10E-05	0.0167	Cross-reference (Pfam)

Supplemental Experimental Procedures.
Bioinformatic Processing and Criteria.

Protein abundances and differential expression were evaluated using two approaches: First, we compared protein abundances using a Label Free Quantitation (LFQ)-based approach (parameter set in Supplementary Information). We did not use “Match-between-runs” and increased the MQ-data point count to four data points. The data were processed and statistically evaluated using the Perseus software package (ver. 1.5.2.6). Protein localization for 5775 of 5940 newly-synthesized proteins were imported from the LocTree3 database (ver. 07-04-2016; Goldberg et al., 2014). Venn diagrams were generated using VennDIS (Ignatchenko *et al.*, 2015). After filtering for the presence in ≥ 2 biological replicates (4471 proteins remaining) and for a coefficient of variation < 1 (4186 proteins remaining), these proteins were annotated using UniprotKB (GOCC, GOMF, GOBP, Reactome, Pfam, InterPro, protein families) and a 1D annotation enrichment was performed (Perseus, Benjamini-Hochberg FDR = 0.05, both side test, Table S8; Cox et al., 2012) and FunRich 2.12 (Benjamini -Hochberg FDR = 0.05 for enrichment analysis; Pathan et al., 2016). Relative protein abundances analysis and statistical significance of differential regulation between control and Bicuculline, control and Tetrodotoxin or Bicuculline and Tetrodotoxin were calculated using an ANOVA test (S0=0.2, permutation-based FDR = 0.05, 250 randomizations) yielding 307 differentially regulated proteins. Proteins were mapped onto KEGG pathways (Kanehisa et al., 2016) using Incromap and bioDBnet Converter, and pathway schemes were adapted and modified (http://www.genome.jp/kegg/). Grey boxes in figures indicate detected proteins, asterisks show statistical significance in the Fisher LSD test (in Origin 2015G Sr1, OriginLab).

For visualization of the network between the 307 differentially regulated proteins, these proteins were analysed using String (v. 10.0, string-db.org; Szklarczyk et al., 2015) with experiments and databases as active interaction sources (textmining, co-expression, neighborhood, gene fusion, co-occurrence disabled; minimum required interaction score: medium, confidence 0.4, query proteins only). Proteins were plotted in custom pathway schemes (modified from KEGG) and LFQ data depicted in call-out boxes for fold changes > 0.5 (manual addition of selected candidates of interest). The 307 differentially regulated proteins were analyzed for correlation with diseases using GeneAnalytics (“MalaCards”, Lifemap Sciences), and association with diseases listed in brief in Figure 8 and in detail in Table S7.

Second, we set out to analyze proteins identified exclusively in one condition. As no high-abundance proteins with high peptide counts and intensity values were detected exclusively, we analyzed proteins with low peptide and intensity counts that displayed a trend in abundance/detection for a specific condition. Here, we first manually evaluated candidates detected exclusively in 3/5 biological replicates in one condition and not detected in any other condition and examined their normalized peptide intensities to exclude detection-limit-based false positive hits. Only proteins displaying similar peptide counts and intensity profiles (based on normalized peptide intensity values) were retained for further analysis. All candidates including peptide counts, intensity values and fold-changes are listed in Table S2, page 2. We also made use of unique peptide ratios and normalized peptide intensity values for manually selected low-abundance proteins. We again focused on proteins with < 5 peptides in a group without robust LFQ data. We approximated abundance by comparing unique peptide counts and extracted proteins with D > 0.6 or D < 0.4 (D= x / x+y, with x = peptides in condition A and y peptides in condition B) and only retained proteins displaying similar trends in normalized intensity ratios. Due to the low peptide counts and intensity values available, the analyses described in the first part do not quantitative values, but rather suggest a trend in abundance.

Supplemental references

Cox, J., and Mann, M. (2012). 1D and 2D annotation enrichment: a statistical method integrating quantitative proteomics with complementary high-throughput data. BMC Bioinformatics 13, S12.

Goldberg, T., et al. (2014). LocTree3 prediction of localization. Nucleic Acids Res 42, W350-W355.

Ignatchenko, V., Ignatchenko, A., Sinha, A., Boutros, P.C., and Kislinger, T. (2015). VennDIS: a JavaFX-based Venn and Euler diagram software to generate publication quality figures. Proteomics 15, 1239-1244.

Kanehisa, M., Sato, Y., Kawashima, M., Furumichi, M., and Tanabe, M. (2016). KEGG as a reference resource for gene and protein annotation. Nucleic Acids Res 44, D457-D462.

Pathan, M., Keerthikumar, S., Ang, C.S., Gangoda, L., Quek, C.M.J., Williamson, N.J., Mouradov, D., Sieber, O.M., Simpson, R.J., Salim, A., Bacic, A., Hill, A.F., Stroud, D.A., Ryan, M.T., Agbinya, J.A., Mariadasson, J.M., Burgess, A.W. and Mathivanan, S. (2015). FunRich: a standalone tool for functional enrichment analysis. Proteomics 15, 2597-2601.

Szklarczyk, D., Franceschini, A., Wyder, S., Forslund, K., Heller, D., Huerta-Cepas, J., Simonovic, M., Roth, A., Santos, A., Tsafou, K.P., Kuhn, M., Bork, P., Jensen, L.J., and von Mering, C. (2015). STRING v10: protein-protein interaction networks, integrated over the tree of life. Nucleic Acids Res 43, D447-D452.
